# Comprehensive evaluation of RNA-seq analysis pipelines in diploid and polyploid species

**DOI:** 10.1093/gigascience/giy132

**Published:** 2018-11-10

**Authors:** Miriam Payá-Milans, James W Olmstead, Gerardo Nunez, Timothy A Rinehart, Margaret Staton

**Affiliations:** 1 Department of Entomology and Plant Pathology, University of Tennessee, 370 PBB, 2505 EJ Chapman Blvd, Knoxville, TN, 37996, United States; 2 Horticultural Sciences Department, University of Florida, 2550 Hull Rd, PO Box 110690, Gainesville, FL, 32611, United States; 3 Thad Cochran Southern Horticultural Laboratory, USDA-Agricultural Research Service, PO Box 287, Poplarville, MS, 39470, United States; 4 Crop Production and Protection, USDA-Agricultural Research Service, 5601 Sunnyside Ave, Beltsville, MD, 20705, United States

**Keywords:** RNA-seq, pipeline, polyploid, correction, trimming, assembly, clustering, reference genome, mapping

## Abstract

**Background:**

The usual analysis of RNA sequencing (RNA-seq) reads is based on an existing reference genome and annotated gene models. However, when a reference for the sequenced species is not available, alternatives include using a reference genome from a related species or reconstructing transcript sequences with *de novo* assembly. In addition, researchers are faced with many options for RNA-seq data processing and limited information on how their decisions will impact the final outcome. Using both a diploid and polyploid species with a distant reference genome, we have tested the influence of different tools at various steps of a typical RNA-seq analysis workflow on the recovery of useful processed data available for downstream analysis.

**Findings:**

At the preprocessing step, we found error correction has a strong influence on *de novo* assembly but not on mapping results. After trimming, a greater percentage of reads could be used in downstream analysis by selecting gentle quality trimming performed with Skewer instead of strict quality trimming with Trimmomatic. This availability of reads correlated with size, quality, and completeness of *de novo* assemblies and with number of mapped reads. When selecting a reference genome from a related species to map reads, outcome was significantly improved when using mapping software tolerant of greater sequence divergence, such as Stampy or GSNAP.

**Conclusions:**

The selection of bioinformatic software tools for RNA-seq data analysis can maximize quality parameters on *de novo* assemblies and availability of reads in downstream analysis.

## Background

**Figure 1:**
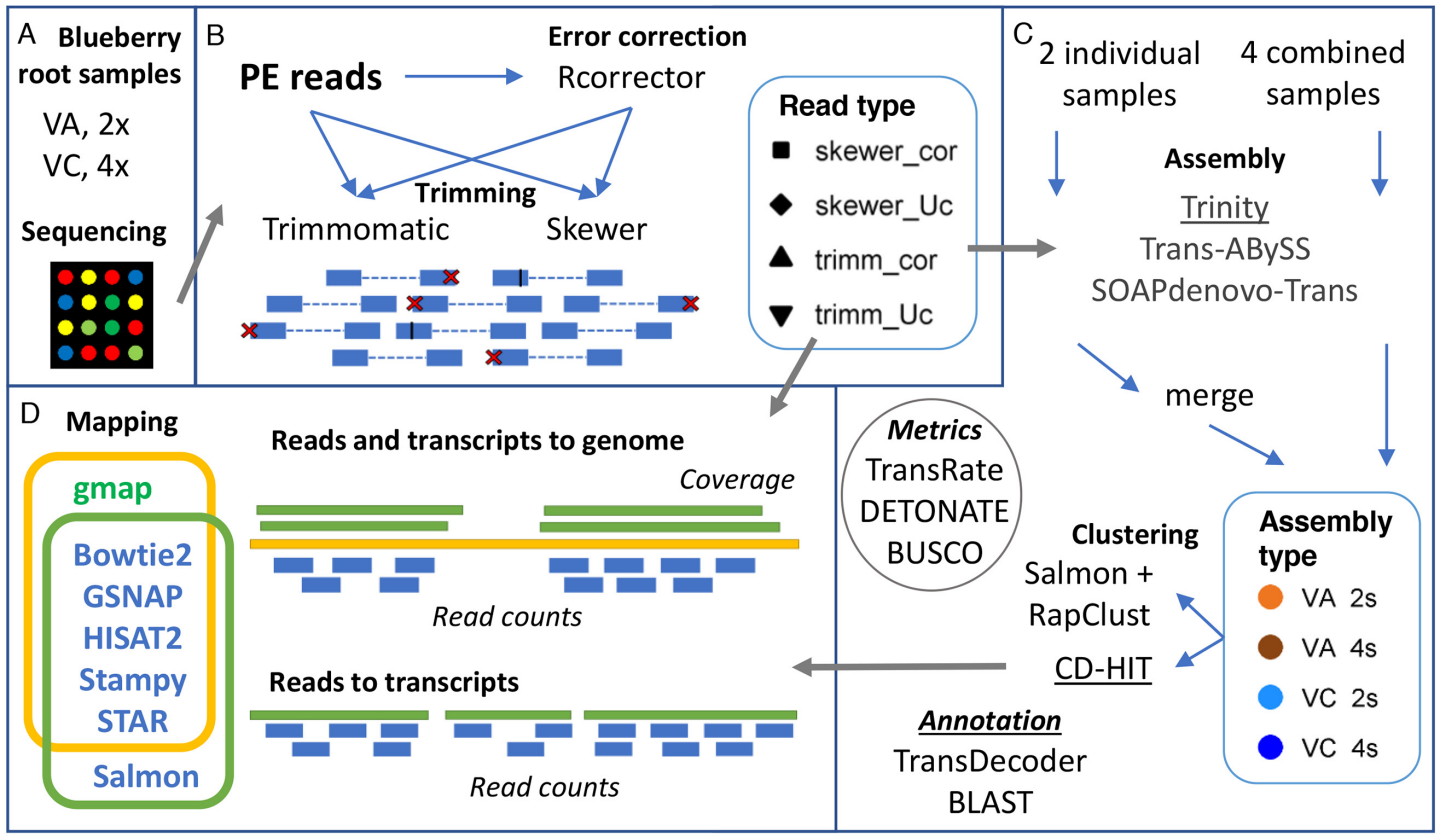
Schematic view of the RNA-seq pipeline followed on this work. **(A)** Samples were obtained from roots of the diploid *Vaccinium arboreum* (VA) and tetraploid *Vaccinium corymbosum* (VC) grown at either pH 4.5 or 6.5 and sequenced. **(B)** Paired-end (PE) Illumina reads were either error corrected (cor; black lines) or not (Uc) and trimmed for removal of adapters and either low-quality bases (trimm; red crosses) or not (skewer). **(C)** Each set of reads was subjected to two *de novo* transcriptome assembly methods (two individual samples and merge results, or four combined samples) with three assemblers, followed by redundancy reduction by CD-HIT and RapClust clustering methods. Metrics were conducted on all steps. Trinity transcriptomes were further annotated, and their CD-HIT clusters used for mapping (underlined). **(D)** Transcripts were mapped to a diploid VC genome with gmap for mapping metrics, while short reads were mapped to either the genome or a transcriptome using multiple read aligners to obtain read counts.

Bioinformatics is a field under constant expansion with regular advances in the development of software and algorithms. This requires researchers to continuously evaluate available software tools and approaches to maximize accuracy of experimental outcomes [1]. However, the majority of the relevant studies comparing bioinformatic tools for RNA sequencing (RNA-seq) data focus on straightforward scenarios with diploid eukaryotes with an available reference genome [2–5]. The implications of data analysis decisions are less clearly understood in situations where, e.g., the species of interest is a polyploid or the species of interest does not have a reference genome, but a reference genome is available from a sister clade. This study aims to explore RNA-seq data analysis from this scenario, where the main steps are read trimming, either mapping to a related species reference genome (from here on referred to as a “distant reference”) or to a *de novo* transcriptome assembly, and read quantification by gene or transcript (Fig. 1). Moreover, this study compares decisions along the RNA-seq analysis steps of a workflow, examining all permutations of those decisions from the beginning to the end of the pipeline.

From the many next-generation sequencing platforms that generate RNA-seq data, Illumina has had the greatest success, yielding high-quality reads at a reasonable price and read length increasing with new generations of instruments [6]. An alternative to RNA-seq for the study of transcriptomes is isoform sequencing, a method developed by Pacific Biosciences to analyze molecules 1–6 Kb long. This method has the advantage of capturing full transcripts but is significantly more expensive per base and thus currently less commonly used than RNA-seq [7]. From raw RNA-seq reads, numerous informatic analysis decisions must be made to derive meaningful biological data, starting with any preprocessing of the reads. Despite the usually high accuracy of Illumina reads (0.1% error rate), error correction is a method with potential to improve the quality of read alignment and *de novo* assembly [8]. Before sequencing, adapters are incorporated to both ends of each sequence. Trimming of bases originating from these adapters is required, but the merit of aggressive vs gentle trimming of lower-quality bases, which modifies the final amount of data, is still being explored [9].

After preprocessing, if a reference genome is available, RNA-seq reads may be used to call variants or determine differentially expressed genes; on the contrary, *de novo* assembly may be used to reconstruct transcripts to do such analyses [10]. *De novo* transcriptome assembly in plants is complex due to the sequence similarity of transcripts that are isoforms, paralogs, orthologs, and, in the case of polyploids, homoeologs. Moreover, in transcriptomes of plants under environmental stress, alternative splicing is even more prevalent [11]. During *de novo* assembly, this complexity is reflected in the form of bubbles or extra branches in de Bruijn graphs that may lead to imperfect assemblies, with a portion of assembled transcripts affected by artifacts such as hybrid assembly of gene families, transcript fusion (chimerism), insertions in contigs, and structural abnormalities such as incompleteness, fragmentation, and local misassembly of contigs [12, 13].

From the many assemblers developed to use with short reads, Trinity [14] is often selected and usually produces good-quality assemblies at single *k*-mer [4, 15]. Trans-ABySS [16], which has good recovery of full transcripts, and SOAPdenovo-Trans [17], designed to handle difficulties of plant genes assembly, are also prevalent. A next step to refine *de novo* assemblies is often to further reduce transcript redundancy. One popular tool is CD-HIT [18], which removes shorter redundant sequences based on sequence similarity. A more recently released clustering tool, RapClust [19], generates clusters based on the relationships exposed by multimapping sequencing fragments and is considerably faster than previous approaches. Several methods are available to assess the overall quality, accuracy, contiguity, and completeness of a *de novo* assembled transcriptome, including basic metrics for assemblies, contig-level metrics, reference-free evaluation methods that include read support, and comparison to protein datasets from related species [10, 12, 20–22].

Read mapping is a crucial step to estimate gene expression for further analysis but is made difficult by sequencing errors and is dependent on characteristics of the reference (e.g., quality of gene annotation, relatedness to sequenced individuals, size, repetitive regions, ploidy) [23]. Mapping transcript reads to a reference genome has the additional challenge of crossing splice junctions, some of which may not be accurately annotated [3]. Multiple metrics can be used to determine performance of read aligners. Precision and recall are the usual metrics with simulated data, while evaluations without *a priori* known outcomes utilize mapping rate, base mismatch rate, detected transcripts, or correlation of gene expression estimates to quantify performance [2, 24]. These outcomes are dependent on the individual implementations of each alignment software package. Many short-read aligners are based on hash tables, with quick seeding of alignment candidates and alignment extension with precise algorithms. These are more sensitive but usually slower than those based on the ultrafast FM-index (Full-text index in Minute space) and extension by dynamic programming, which are fast though less flexible with handling errors [2, 10]. When using a distant genome, sequence divergence between reads and the reference genome may compromise results; nucleotide mismatches are more likely to decrease the number of mapped reads, while indels are usually better tolerated with gapped alignments [2]. One benefit from the utilization of a distant genome is a direct comparison of gene expression results from multiple related species [25]. On the other hand, utilization of *de novo* assemblies avoids the mapping issues to a distant genome and also captures divergent and novel genes useful for species-specific discovery of new functions. Selecting between a *de novo* transcriptome or a reference genome has been shown to produce comparable gene expression profiles at over 87% correlation in other systems but has not been examined in plants [5, 24].

Most prior research that examined the choice of informatics software for RNA-seq data analysis worked with straightforward datasets, either performing a single type of analysis on the data or working with data from diploid organisms with well-developed reference genomes. However, much less research has been done into genomics of complex species and, especially in the case of plants, polyploids. Many polyploid crops now have available reference genomes, such as strawberry [26], cotton [27], wheat [28], and sweet potato [29], while others continue to rely on genomic resources from diploid relatives, such as potato [30], kiwifruit [31], peanut [32], and blueberry [33]. Here, we have selected blueberry datasets as an example. A number of different species of blueberries are used in agricultural production and breeding, with autotetraploid *Vaccinium corymbosum* (VC; highbush blueberry) as the most economically important [34]. A diploid accession of *V. corymbosum* was used for genome sequencing and construction of a blueberry reference genome [33, 35]. In this study, we use RNA-seq data from an autotetraploid *V. corymbosum* (section *Cyanococcus*) and a diploid species, *Vaccinium arboreum* (VA;section *Batodendron*).

## Data Description

The sequencing data used in this work is 270 million Illumina paired-end reads (2*101 bp long) for diploid VA and 582 million reads for tetraploid VC, originating from eight plants each [25] and sequenced on duplicate lanes. Libraries were prepared from RNA collected from roots of plants of similar age after eight weeks of growth in hydroponic systems under either stressful (pH 6.5) or control (pH 4.5) conditions. All sequence data are publicly available at the National Center for Biotechnology Information (NCBI) (see details below). At the first step of data curation, our tested methods are error correction of RNA-seq data with Rcorrector and trimming of low-quality bases by one of two methods, Trimmomatic [36] or Skewer [37] (Supplementary Table S1). Error correction of raw reads modified an average of 0.7% bases per library, a proportion larger than the expected 0.1% sequencing error rate in Illumina reads and suggesting a possible masking of variability in the data. Next, both original and corrected reads were trimmed using either Skewer or Trimmomatic at default settings. Gentle quality trimming with Skewer retained on average 99.6% reads at mean length 99.8 bp (Supplementary Table S2). In contrast, quality trimming with Trimmomatic, which has significantly more aggressive default trimming parameters, retained 77.2% of reads at mean length 93.8 bp. Error correction had a minimal effect on trimming results. From the combination of corrected/uncorrected reads and trimming software used, four read sets (reads processed by Rcorrector and Trimmomatic, Rcorrector and Skewer, Trimmomatic only, and Skewer only) for each species were used in downstream analyses.

## Analysis

### Generation of *de novo* transcriptome assemblies

A series of *de novo* assemblies were carried out with Trinity, SOAPdenovo-Trans, and Trans-ABySS software packages (Supplementary Table S1). For each species, assemblies of a single control library, a single treatment library, or a combination of both libraries were performed, using each of the four preprocessing techniques as input (Skewer corrected, Skewer uncorrected, Trimmomatic corrected, Trimmomatic uncorrected), to yield 24 initial runs from each assembler (Fig. 1 and Supplementary Fig. S1). For the assembly of two individual libraries, the results were combined post-assembly (Fig. 1 and Supplementary Fig. S1). The possible benefit of this approach is the reconstruction of specific transcripts from control and treated samples without mixture of alternative splice variants, at the expense of including a smaller data input size that may induce fragmentation of assemblies as well as a requirement to merge the separate assemblies afterward. This approach is contrasted to the second method, which combines multiple samples in a single assembly run; this approach aims at reconstructing longer and more complete transcripts despite mixing fragments from splice variants.

**Figure 2:**
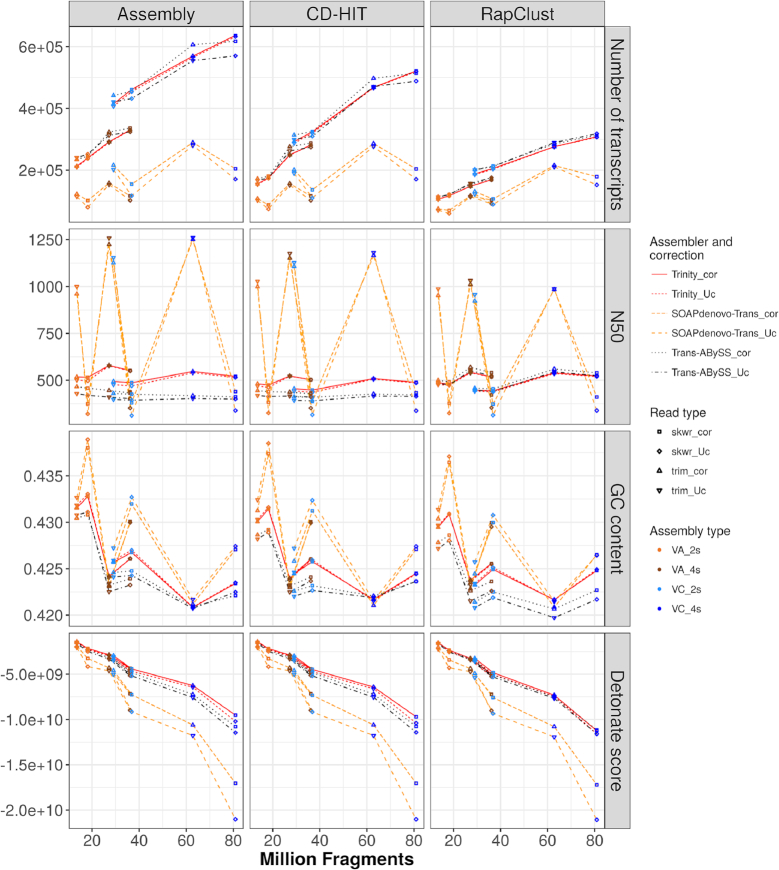
Basic statistics of *de novo* transcriptome assemblies and CD-HIT or RapClust reduced transcriptomes. Individual assemblies are plotted with the number of input fragments along the *x*-axis. Lines are drawn to visually associate assemblies from the same species, assembler (SOAPdenovo-Trans, Trans-ABySS, or Trinity) and error correction strategy (with or without Rcorrector). Total number of transcripts, N50 value, percent of GC content, and Detonate scores (rows) are shown for initial assemblies, assemblies clustered with CD-HIT, and assemblies clustered with RapClust (columns). Point colors indicate species and number of samples used on assembly. Point shapes indicate use of error correction (cor) or not (Uc) and trimming software (Skewer or Trimmomatic).

Trinity, SOAPdenovo-Trans, and Trans-ABySS responded differently to the number of input reads and how they are preprocessing (Fig. 2). Trinity and Trans-ABySS produced transcriptomes with similar numbers of transcripts, generally increasing with the number of input reads, and with similar N50 scores. By contrast, SOAPdenovo-Trans produced transcriptomes with 27%–52% fewer transcripts (80,000–290,000 sequences). SOAPdenovo-Trans also demonstrated more sensitivity to the trimming and correcting methods, with the use of Trimmomatic yielding a larger number of transcripts and increased N50 statistic. For both species, the highest observed N50 was achieved by uncorrected Trimmomatic-trimmed reads and four input samples assembled with SOAPdenovo-Trans. On the contrary, Skewer-trimmed reads had reduced transcript numbers and N50. The N50 from Trinity and Trans-ABySS assemblies followed a more constant pattern, with Trinity reaching a higher N50 (440–580 bp) compared to 390–465 bp from Trans-ABySS. Trinity also yielded a higher N50 in VA than VC and a slight improvement when using four samples. Detonate [22], a reference-free evaluation tool, was used to compare each set of transcriptomes formed from the same set of reads, where scores closer to zero indicate better assemblies. Transcriptome quality as assessed by Detonate was highest in Trinity, closely followed by Trans-ABySS; error correction and use of Trimmomatic had a positive impact on these metrics.

GC content of final transcriptome assemblies also varied by assembly strategy. Our results (Fig. 2) contained assemblies of 42.3%–43.9% GC for VA and 42.1%–43.3% GC for VC, with the highest variability across samples found with SOAPdenovo-Trans. GC content was generally higher and more variable when reads were preprocessed by Skewer, possibly indicating the role of residual primer sequences or low-quality bases in lowering final GC content. When input reads were trimmed with Trimmomatic, assemblies generally had very similar GC content across assemblers. The assemblies for VC four-sample (4s) with Trimmomatic had GC content between 42.1% and 42.2%, matching the 42.2% of predicted VC gene models from the reference genome [33]; VA transcriptomes had 42.3%–42.4% GC under the same conditions.

**Figure 3:**
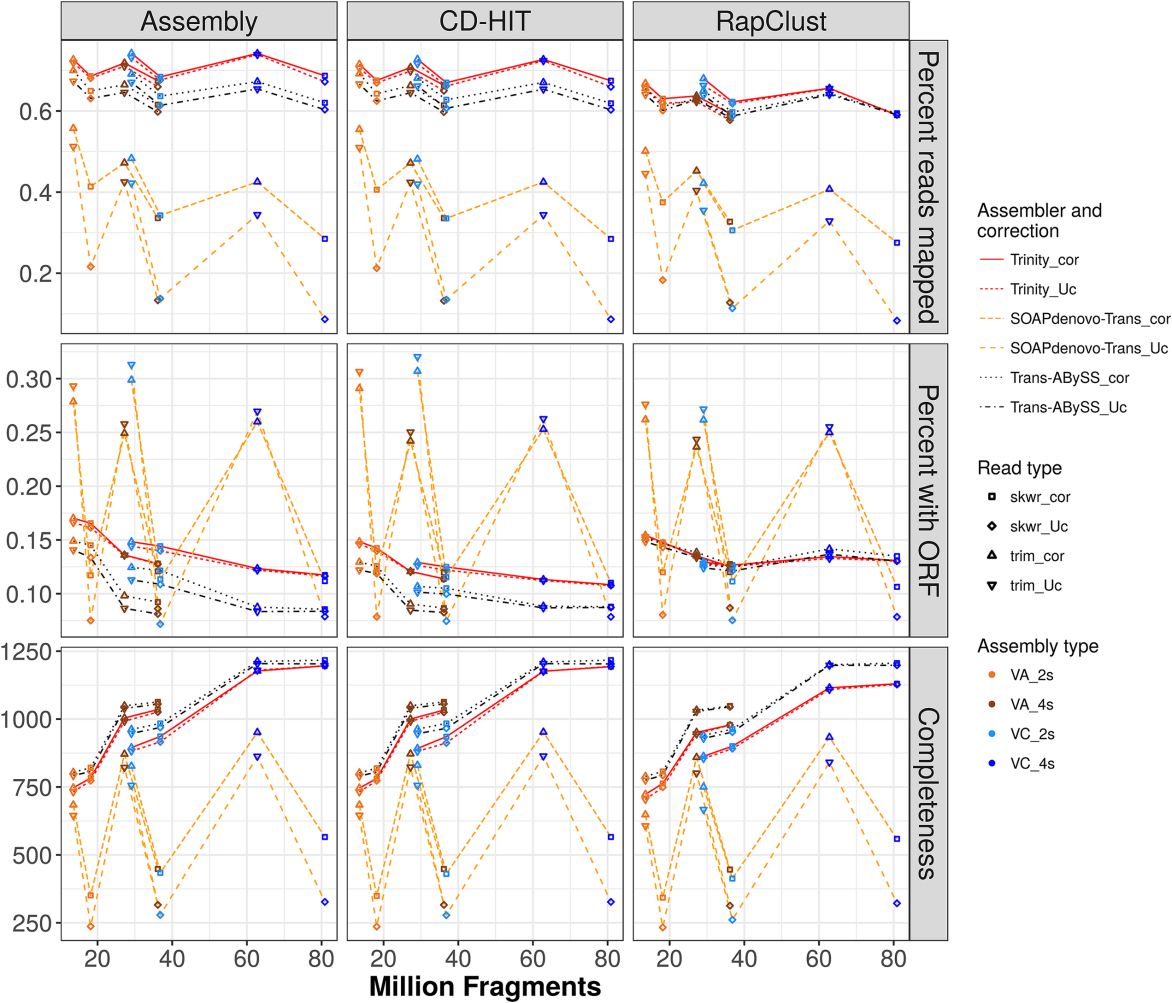
Read and annotation support of *de novo* transcriptome assemblies and CD-HIT or RapClust reduced transcriptomes. Quality metrics for assemblies, including percent of input reads that map back to assemblies, the proportion of transcripts with a putative open reading frame (ORF), and completeness as determined by the presence of Benchmarking Universal Single-Copy Orthologues orthologs (rows). These metrics are represented for initial assemblies, assemblies clustered with CD-HIT, and assemblies clustered with RapClust (columns). Lines are drawn to visually associate assemblies from the same species, assembler (SOAPdenovo-Trans, Trans-ABySS, or Trinity), and error correction strategy (with or without Rcorrector). Point colors indicate species and number of samples used for assembly; point shapes indicate use (cor) or not (Uc) of error correction and trimming software (Skewer or Trimmomatic).

Quality assessment can also be measured as the proportion of RNA-seq reads used to generate each assembly that map back to the transcriptome (Fig. 3). Read support (percent reads mapped, top row) was best for Trinity, ranging from 66% to 74%, followed by Trans-ABySS with 60%–70%, and was very variable in SOAPdenovo-Trans, 9%–56%. Strict trimming with Trimmomatic and error correction had an overall positive impact on read support. All assemblers showed reduced read mapping with uncorrected reads and Skewer trimming; the trend was most pronounced for SOAPdenovo-Trans, with more than 30% average reduction in mapping rate when using Skewer uncorrected than Trimmomatic corrected reads.

In addition to assembly metrics, functional annotation of transcripts was done to assess putative biological information contained in the transcriptomes. An initial observation of putative coding regions consisted of finding complete open reading frames (ORFs) with at least 50 amino acids from start to stop codon. SOAPdenovo-Trans showed strong variations by trimming software, with Skewer transcriptomes having 7%–12% of transcripts with predicted ORF vs 25%–31% with Trimmomatic (Fig. 3). Trinity, between 12% and 17%, had 2%–5% higher content on ORFs than Trans-ABySS, which ranged from 8% to 15%. Finally, assemblers were compared as a function of completeness of their assemblies, indicated by the total number of conserved orthologs (Benchmarking Universal Single-Copy Orthologues [BUSCO]) present in the transcriptomes, from 1,440 plant BUSCOs. Trans-ABySS yielded the assemblies with the highest completeness, with 792–1,217 identified BUSCOs, closely followed by Trinity with an average of 40 fewer BUSCOs per transcriptome. SOAPdenovo-Trans again showed strong variation with trimming type, yielding between 237 and 566 BUSCOs with Skewer and between 645 and 951 with Trimmomatic.

Overall, these results show the impacts error correction, trimming, and assembly software can have on transcriptome assembly results. Error correction contributed to transcriptomes with more transcripts, with higher completeness, and with decreased GC content; for Trinity and Trans-ABySS, error correction promoted higher N50 and ORF content while decreasing percent of reads mapping back to transcriptomes. These results are in agreement with previous reports showing improvement of assembly quality after using an error correction tool [8, 38]. Use of strict trimming, such as with Trimmomatic, generally improved transcriptome metrics and all Detonate scores, with a smaller number of total transcripts, improved N50, more consistent GC content, better rate of mapping of reads, and higher proportion of coding regions, with very little loss of completeness when using four samples. Use of Skewer-trimmed reads had a particularly negative effect on SOAPdenovo-Trans, including reduced number of transcripts, reduced N50, reduced Detonate score, lower percent of reads mapping, much lower number of identified ORF, and lower completeness. VA transcriptomes differed from those of VC with a generally lower number of transcripts and higher Detonate scores. With use on the Trans-Abyss and Trinity assemblies, more differences in VA vs VC can be observed, including slightly higher N50 and identified ORFs in VA assemblies, but more completeness in VC assemblies. Using two samples yielded fewer transcripts and a lower percent of reads mapped and lower completeness than those from four samples, despite their higher Detonate scores.

### Clustering of *de novo* assemblies

Assemblies may contain sequences from highly similar gene isoforms, transcript isoforms of the same gene, and, in the case of polyploids, homoeologous genes that may be considered redundant and lead to reads mapping to multiple locations. In addition, considering that plants contain 37,000 proteins on average [39], the number of transcripts from all of the *Vaccinium* assemblies (Fig. 2) largely surpasses this quantity. Tools aimed at the reduction of such redundancy are widely used to select nonredundant representative sequences [15, 40, 41]. We have compared the clustering capabilities of two tools with very different approaches (Supplementary Table S1). CD-HIT was used to select long representative transcripts and remove smaller redundant sequences at 95% similarity cutoff. RapClust groups transcripts based on the information of multimapped reads and removes transcripts with low read support. CD-HIT returns a classification of transcripts into clusters and a set of representative transcripts with reduced redundancy, while RapClust returns clustering information suited to be used for downstream differential expression analysis but does not report a reduced transcript set. For the sake of comparing results, the longest transcript from each cluster generated by RapClust was selected to form corresponding reduced assemblies. Prior to clustering, single-sample assemblies were combined into a merged assembly, with expected introduction of high redundancy. Then, transcripts from the 16 assemblies (8 per species) and 3 assemblers (Fig. 1 and Supplementary Fig. S1) were subjected to classification into clusters with either of these tools.

Clustering had a noticeable impact on assemblies (Fig. 2), with RapClust producing fewer clusters in comparison to CD-HIT's reduced transcript set in all cases. Noticeably after application of RapClust, Trinity and Trans-ABySS assemblies had a very similar number of transcripts, N50, and Detonate scores. On average, the number of clusters after CD-HIT and RapClust were 22% and 51% smaller than the initial number of transcripts, respectively, for both Trinity and Trans-ABySS, and 5% and 26% after SOAPdenovo-Trans. To a lesser extent, the degree of clustering varied by type of assembly and species. Despite the 4s assemblies having larger initial numbers of transcripts, the percent of removed or clustered transcripts was greater in two-sample (2s) than 4s assemblies. Thus, after clustering, a larger proportion of representative sequences was retained on 4s assemblies compared to 2s assemblies by 12%, 13%, and 8.7% by CD-HIT, or 2.5%, 3.3%, and 15% by RapClust, on Trinity, Trans-ABySS, and SOAPdenovo-Trans, respectively. Clustering only showed small difference by species with Trinity assemblies, with 3.2% more sequences retained as clusters in VA than VC. These trends are likely due to the putative higher redundancy in 2s assemblies and the presence of homoeolog genes due to polyploidy in VC. Clustering has a variety of impacts on N50. The N50 of Trinity assemblies was not much changed, while the N50 for Trans-ABySS assemblies was increased. For SOAPdenovo-Trans, the N50 was reduced after clustering, particularly with Trimmomatic trimming, from the highest N50 of 1,260 to 1,180 and 1,030 after CD-HIT and RapClust, respectively. Detonate scores were used to evaluate the original assembled transcripts with the three assemblers as well as the cluster representative sequences yielded by CD-HIT and the longest transcript from each RapClust cluster. Clustering with CD-HIT did not substantially modify Detonate scores, while for RapClust, Trinity scores were slightly lowered.

GC content of clustered assemblies (Fig. 2) was reduced by an average 0.2% from the original assemblies in those from two samples and generated with Trinity or Trans-ABySS. The same reduction was observed in 2s assemblies when using RapClust on SOAPdenovo-Trans assemblies. In all cases, values were reduced closer to the putative GC percent found in the diploid VC reference genes. All changes were minor, with most assemblies from four samples and Trimmomatic-trimmed reads staying close to their original values after clustering. Clustering yielded a less than 5% decrease in support from RNA-seq reads of the transcriptomes generated with Trans-ABySS and SOAPdenovo-Trans (Fig. 3) or clustered with CD-HIT. Trinity assemblies had an average of 7% loss of read support under clustering with RapClust, which is close to Trans-ABySS values but still having the highest support. Differences in ORF content between Trinity and Trans-ABySS decreased with clustering as Trans-ABySS modified ORF content from 8%–15% to 8%–12% after CD-HIT and 12%–15% after RapClust, while Trinity changed from 12%–17% to 11%–15% and 13%–15% after CD-HIT and RapClust, respectively. Lower values of SOAPdenovo-Trans remained at 7% after clustering, but the highest ORF content, originally at 31%, changed to 32% and 27% after CD-HIT and RapClust, respectively. The variation of the proportion of transcripts containing a coding sequence was not mirrored by the degree of completeness. Clustering with CD-HIT did not modify the overall completeness of assemblies, while RapClust slightly decreased them by 14, 43, and 24 in Trans-ABySS, Trinity, and SOAPdenovo-Trans, respectively.

Clustering with CD-HIT was effective in reducing the redundancy of transcriptome assemblies in Trinity and Trans-ABySS, without substantial modification of quality metrics. This reduction affected especially 2s assemblies compared to 4s, concomitant with the expected higher artificial redundancy induced in 2s assemblies after the merging of single assemblies. SOAPdenovo-Trans assemblies displayed little modification from CD-HIT clustering, suggesting a lower number of isoforms or less fragmentation in the output transcriptomes. By contrast, RapClust reduced the number of transcripts from all three assemblers, with different effects. SOAPdenovo-Trans assemblies had a lower N50 and ORF content but similar read support, Detonate scores, and completeness after RapClust clustering and selection of the longest transcript as a representative. For Trans-ABySS assemblies, there were similar read support, Detonate scores, and completeness after RapClust but higher N50 and ORF content, suggesting a reduction of smaller and noncoding transcripts. For Trinity assemblies, the similar N50 and ORF content but lower read support, Detonate scores, and completeness suggests a reduction of transcripts of all sizes by RapClust.

### Biological consistency of clustering methods

The general evaluation of *de novo* transcriptome assemblers revealed that Trinity assemblies have balanced metrics across options, with high support of RNA-seq reads, medium N50 and proportion of coding transcripts, and high completeness. Trans-ABySS was competitive on completeness and balanced on GC content but had lower read support, N50, and ORFs. SOAPdenovo-Trans was very sensitive to the input read trimming, showing good metrics with Trimmomatic, but had an overall low read support and completeness compared with the other methods. Thus, from here on, Trinity assemblies are selected to explore in more detail assembly metrics and mapping of RNA-seq reads.

**Figure 4:**
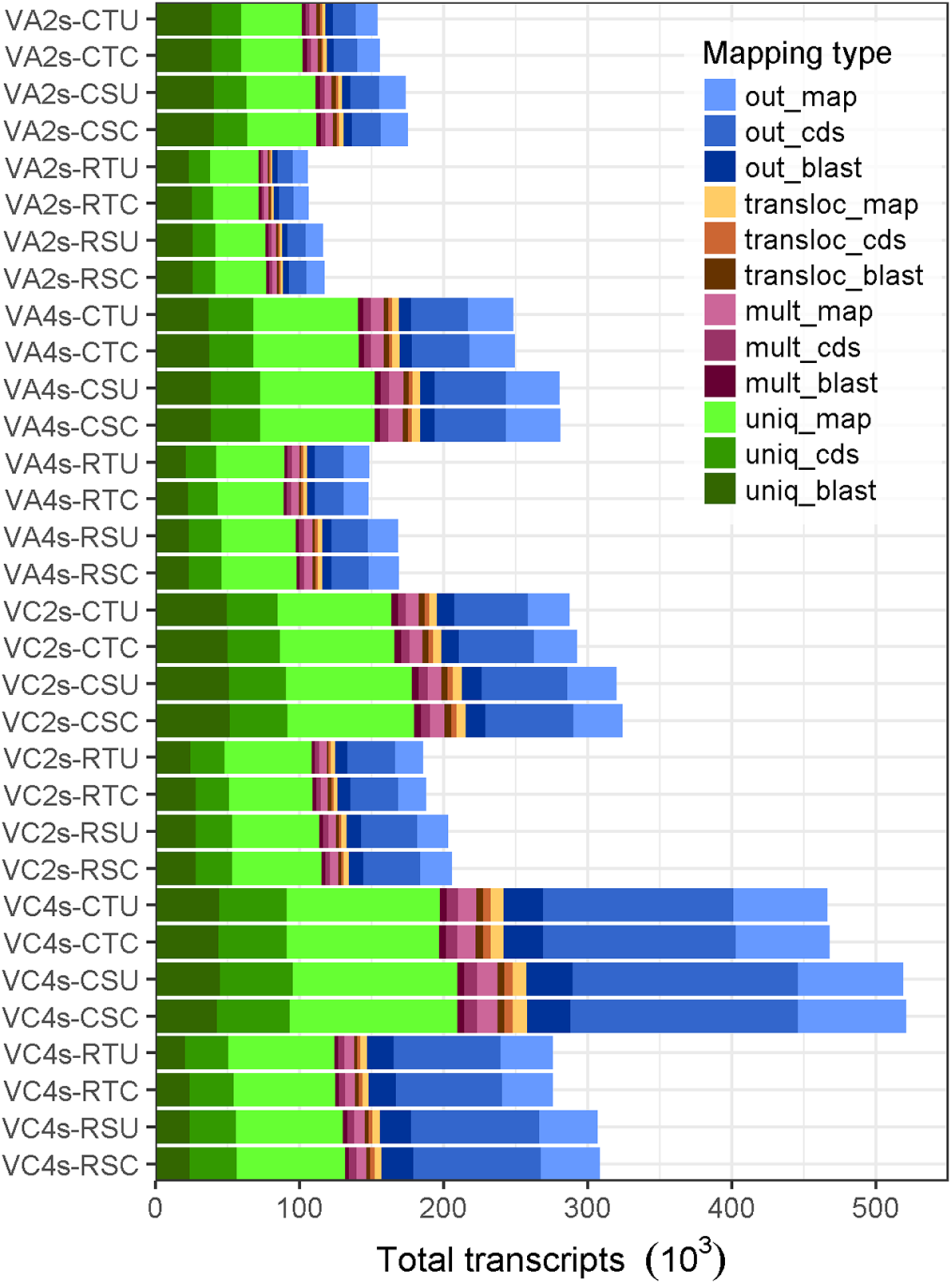
Mapping of *de novo* assembly transcriptomes to *V.corymbosum* reference genome and annotation of transcripts. Transcripts mapped uniquely to the genome (uniq), to multiple locations (mult), with translocations (transloc), or did not map (out). Annotation from prediction of coding sequences (cds) using homology results from blast is divided as “No Functional Annotation” (map), “CDS Only” (cds), and “CDS with Blast Hit” (blast). Transcriptomes for *V. arboreum* (VA) or *V. corymbosum* (VC) produced from two (2s) or four (4s) samples were clustered with either CD-HIT (C) or RapClust (R). The last two letters indicate trimming with Trimmomatic (T) or Skewer (S), and use (C) or not (U) of error correction of RNA-seq reads.

To further explore the effect of clustering, we utilized the published reference genome from the diploid *V. corymbosum* [33]. We presented two scenarios, one with a distant diploid species and the other with the same species but different ploidy level. To explore the portion of transcripts with sequence homology that each species shares with the reference genome, we mapped the clustered transcriptomes to it. Transcripts were classified as uniquely mapping, mapping to multiple loci, translocated (parts of the transcripts were mapped to different locations on the genome), or not mapping. These results were combined with coding sequence (cds) predictions from Transdecoder and blast homology results. Overall, transcripts generated for the diploid VA mapped to the reference genome at a larger proportion than the tetraploid VC, and the two-sample merged assemblies (2s) mapped at a higher rate than the 4s ones (Fig. 4). Specifically, the average mapping rate of transcripts was 66% and 57% in VA 2s and 4s and 57% and 43% in VC 2s and 4s. Thus, the use of multiple samples leads to a higher proportion of transcripts not resembling the genome, representing species-specific transcripts and possibly artifacts. While VA has higher mapping rates than VC, discrimination between a true higher similarity or an effect due to the read input cannot be made. The proportion of multiple mapping and translocated transcripts had little variation across transcriptomes in both species, being 5%–7% and 4%, respectively. Multimapping rate reflects highly similar regions of the genome, and translocations could indicate either true genome rearrangements or assembly artifacts such as transcript fusions (chimeras). Clustering with CD-HIT or RapClust (using a single representative sequence for each cluster), despite affecting the total number of transcripts, maintained similar proportion of transcripts in each mapping category; on average, RapClust increased 2.2% unique and decreased by 0.5% multiple and translocated mapping transcripts compared to CD-HIT. Trimming also influenced mapping; assemblies from reads trimmed with Trimmomatic showed an average 2% higher unique mapping rate than their counterparts with Skewer, suggesting better accuracy with stricter trimming. No effect was observed from error correction.

Prediction of a coding sequence and the extent to which it may be coding for proteins was used as an indicator of biological information contained in transcripts. Transdecoder finds all ORFs and selects the most likely putative cds using homology search results from blast; 51%–59% of transcripts contained a predicted cds for all assemblies (Supplementary Table S3). Compared to the length of original transcripts, the average length of cds decreased by 13% and 20% on 2s and 4s assemblies, respectively. Transcripts within each category (unique, multiple, translocated, and not mapping) had different likelihoods of having a predicted coding sequence and additionally of cds showing homology to known proteins. On average, 49.2%, 51.8%, 54.8%, and 64.5% of the transcripts in the categories unique, multiple, translocated, and not mapping contained a predicted coding sequence (Fig. 4, Supplementary Table S3). In addition, 54.0%, 42.4%, 55.2%, and 20.1% of the cds on those categories, respectively, had a blast hit. Thus, a relatively large proportion of cds do not map to the genome, particularly in VC with four samples (72%). These transcripts also show low similarity to known proteins, leaving unclear whether they belong to true novel transcripts or they are assembly artifacts. For transcripts that mapped to the genome, VA exhibited a greater proportion of annotation than VC. Nonetheless, comparing absolute number of transcripts, VC has a larger set of mapping transcripts with cds but also an even larger number of transcripts not matching the reference than VA. Influence from the other analysis options on annotation distribution was less drastic. Clustering with RapClust had a positive effect on the proportion of cds and blast results of unique and translocated transcripts, especially in 2s assemblies, in the range of 0.5% to 5.5%. Changes due to read trimming or correction were lower than 2%.

**Figure 5:**
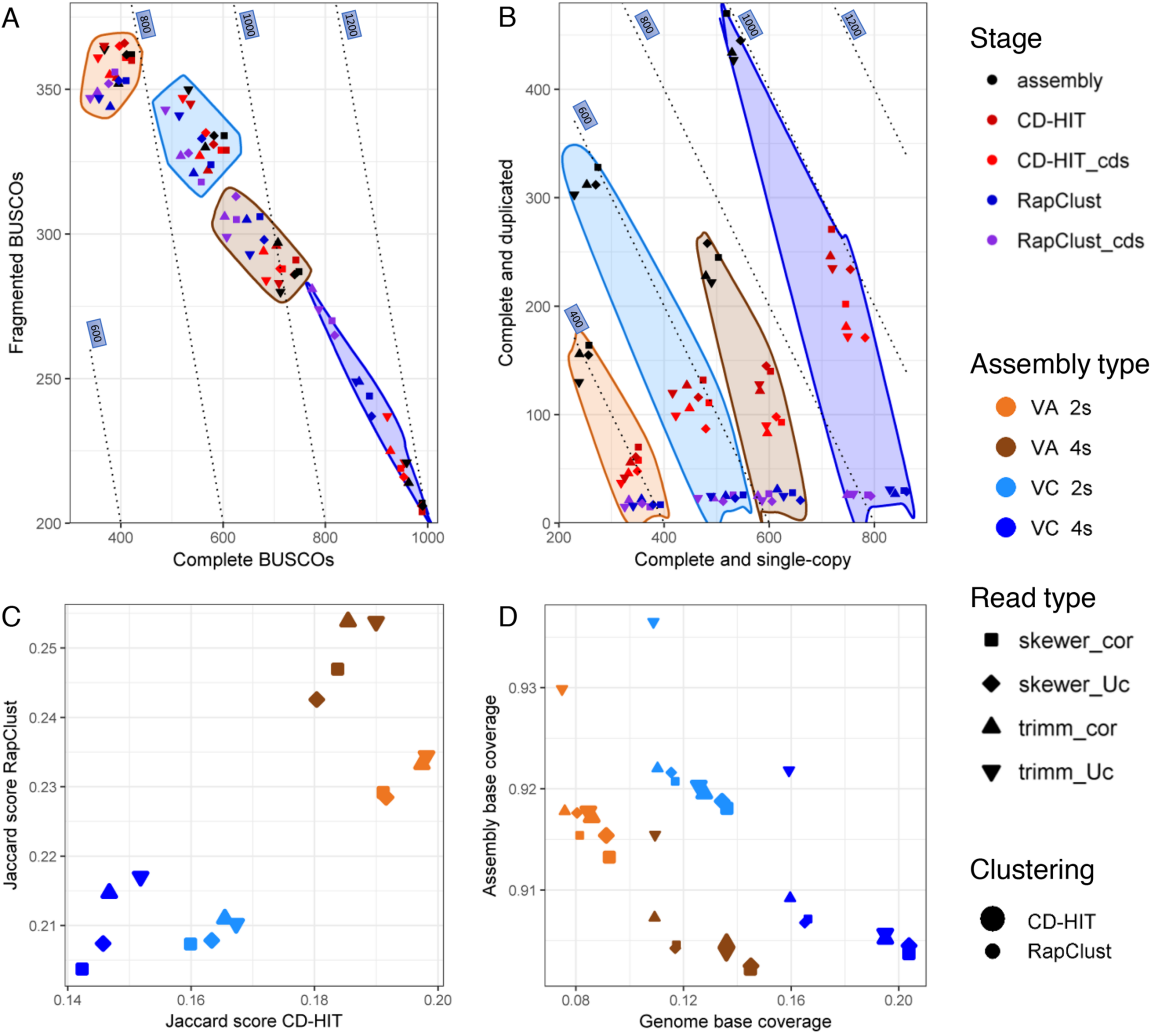
Evaluation of assembly and clustering methods for Trinity transcriptomes. Completeness assessment with BUSCO tool subdivided into complete vs fragmented BUSCOs **(A)** or single-copy vs duplicated complete BUSCOs **(B)**. Dotted lines represent isolines of BUSCO numbers from a total search space of 1,440 orthologs. Dot colors indicate assembly stage and areas assembly type. Stages of the assembly are divided into initial *de novo* assembly (asmb), clustered with either CD-HIT or RapClust, or predicted coding regions (cds). Assembly type indicates the combination of blueberry species (*V.arboreum*, VA; *V. corymbosum*, VC) and the use of two independent assemblies merged (2s) or assembly of four samples (4s). Shapes represent read pre-processing options, with (cor) or without (Uc) error correction, and the use of Skewer or Trimmomatic (trimm) trimming tools. **(C)** Distribution of mean Jaccard scores on CD-HIT and RapClust clusters of transcriptome assemblies. Scores range between ∼0 (low clustering of co-annotated transcripts) and 1 (perfect clustering of co-annotated transcripts). **(D)** Distribution of genome vs assembly base coverage on multiple *de novo* assemblies mapped to *V. corymbosum* reference genome after redundancy reduction with either CD-HIT (larger points) or RapClust (smaller points). Shapes indicate read processing, with (cor) or without (Uc) error correction, and trimmed with either Trimmomatic (trimm) or Skewer.

Specific variations on Trinity transcriptome completeness throughout the sequential stages of processing (i.e., assembly, clustering, and cds prediction) used the BUSCO tool to report, for each of the 1,440 near-universal conserved orthologs searched, whether it is present in the assembly as complete and single-copy, complete and duplicated, fragmented, or missing. Examining the impact on BUSCO results by read processing, assemblies from soft trimmed reads with Skewer presented higher completeness (Fig. 5A). Interestingly, error correction improved the formation of complete BUSCOs on 2s assemblies, while it did not have a significant effect on 4s assemblies. However, the major options influencing completeness were blueberry species and number of samples used. Thus, assembly of complete genes was improved in VC compared to VA and in assemblies of four rather than two samples (Fig. 5A). Overall, completeness of CD-HIT clusters was very similar to those of *de novo* assemblies, while RapClust clusters contained fewer total BUSCOs. Selection of cds further decreased completeness, either decreasing complete genes or also increasing fragmented genes, mostly in 4s assemblies. In addition, the distribution of complete vs fragmented BUSCOs shows a trend where a reduction in total BUSCOs is followed by an increase in fragmented BUSCOs (Fig. 5A). Following this trend, the rate of fragmented BUSCOs was not significantly modified by read processing nor by clustering with CD-HIT, while RapClust increased it except in VA 2s, where fragmented BUSCOs were reduced.

While some gene families may have undergone expansion or contraction since the *Vaccinium* common ancestor, we expect the majority of transcripts to provide one-to-one orthologs for the VA gene set and two-to-one orthologs for the tetraploid VC gene set. Coincident with their ploidy, duplicated vs single-copy ratio in unclustered VA *de novo* assemblies was half that of VC (0.50 in 2s and 0.58 in 4s). Also, the duplication ratio in 2s vs 4s unclustered assemblies was 1.25 in VA and 1.45 in VC, supporting higher redundancy in 2s assemblies. These ratios are independent from the size of transcriptomes. Clustering was efficient to remove redundant genes, as shown by the reduction of duplicates. RapClust drastically removed most duplicated BUSCOs, leaving 20–30 duplicated BUSCOs for all assemblies, while CD-HIT performed a reduction proportional to the assembly length of 62% on 2s and 44% on 4s assemblies. While the clustering did remove many duplicated BUSCOs, most became single-copy BUSCOs and were not lost from the assembly altogether. Only in the 4s assemblies, comparing the original assembly to RapClust cluster transcripts, was there a significant decrease in the number of complete BUSCOs (Fig. 5B). Ideally, clustering would reduce splice isoforms and partially assembled transcripts; however, the reduction in completeness suggests possible removal of gene isoforms in both species and collapse of homoeologs in the tetraploid VC, especially by RapClust.

BUSCO results were not only used to assess completeness but also to measure the success of the clustering methods using an adaptation of the Jaccard similarity method. Taking advantage of BUSCO consensus sequences, transcript co-annotation was calculated as the number of transcripts with the same BUSCO annotation within a cluster (set intersection) divided by the total number of transcripts with that BUSCO annotation or in the cluster (set union). The result is a value in the range of 0 to 1, from low to perfect shared annotation of transcripts within a cluster. This method not only indicates the degree of co-annotation depicted by each clustering algorithm but also compares the putative biological relevance of clusters. In this respect, RapClust consistently outperforms CD-HIT on clustering of co-annotated BUSCO genes (Fig. 5C). Clusters from the diploid VA were markedly better co-annotated than those from VC. Generally, RapClust performance was enhanced on larger transcriptomes, while CD-HIT performed better on smaller ones. In relation to read processing, Trimmomatic and uncorrected reads generally achieved higher scores.

To explore the percent of the blueberry genome captured by the *de novo* assemblies, base coverage was calculated for transcripts that mapped uniquely to the diploid reference genome (Figs. 4 and 5D). Assembly base coverage is the proportion of bases of each transcript assembly that were mapped to the reference genome, and genome base coverage is the proportion of the reference genome covered by the transcripts. In general, both metrics showed inverse correlation. Thus, genome coverage was enhanced with the use of Skewer, four samples, and CD-HIT, while decreasing assembly coverage. Thus, genome coverage is concordantly improved by those options that also increase transcriptome size, where a larger number of transcripts is able to better represent genomic sequences. This is true for both blueberry species, with the distinction that VC exhibits both better genome and assembly coverage than VA, consistent with phylogenetic proximity to the reference genome species. On the other hand, trimming with Trimmomatic, 2s assemblies, and clustering with RapClust had better assembly coverage, but lower genome coverage. This suggests that transcripts generated from more restrictive options are more likely to be real genes that can be found in the genome, but the more restrictive options do exclude some genes. Error correction did not follow this trend and generally decreased assembly coverage while not affecting genome coverage.

### Read mapping to reference genome

**Figure 6:**
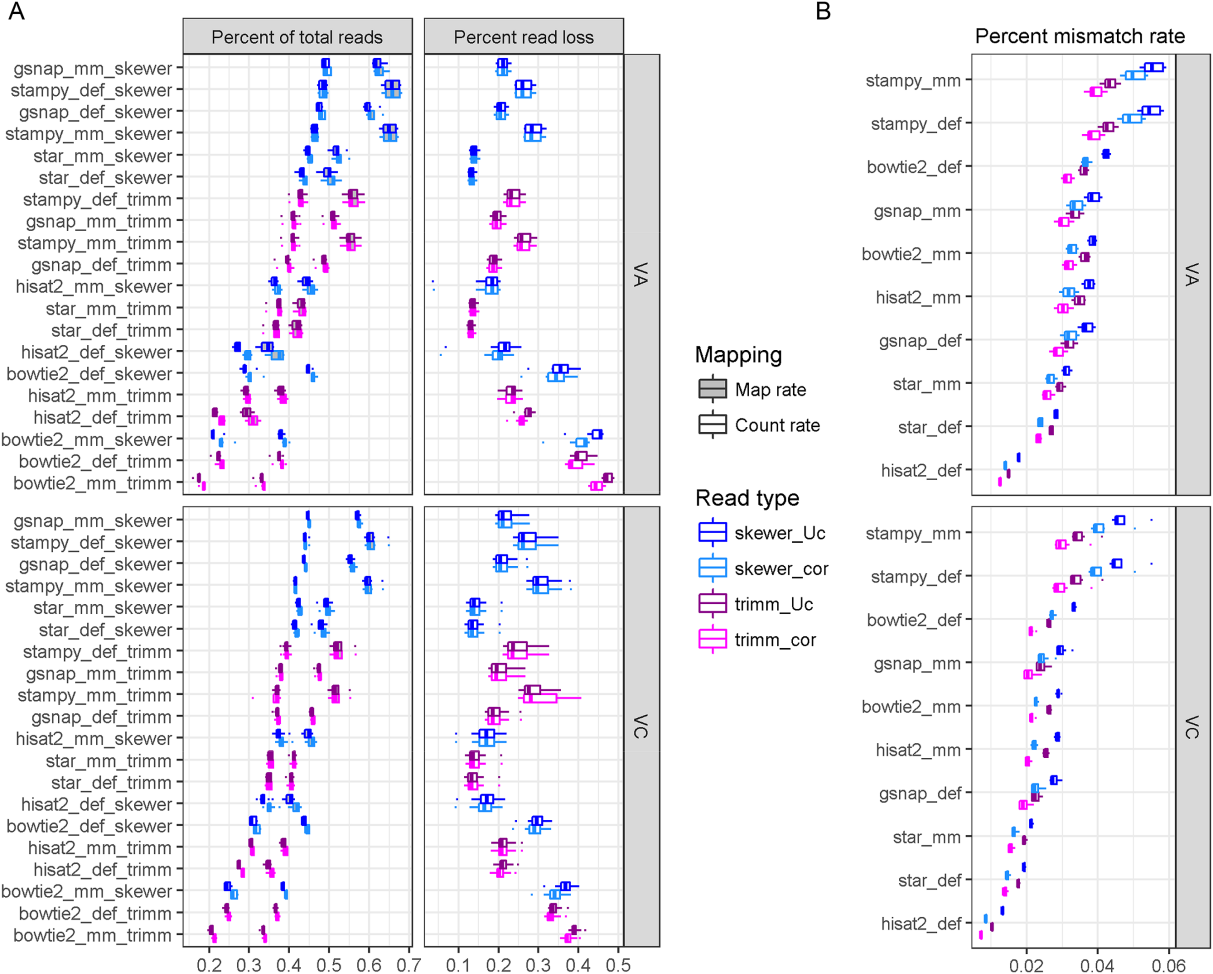
Read mapping to *V. corymbosum* reference genome. **(A, left panels)** Proportion of total reads mapping to reference (gray boxes or higher values) converted to counts (white boxes or lower values) and **(A, right panels)** percentage of the difference.**(B)** Mismatch rate depicted by each software option. Five mapping software programs were compared at default and modified settings to increase mismatch tolerance. Reads used (cor) or not (Uc) error correction and Trimmomatic (trimm) or Skewer trimming software. Results are distribution of eight samples.

As an alternative to *de novo* assembly, RNA-seq analysis for these two species could utilize a mapping approach with the publicly available genome of diploid VC. With this approach, an entirely different set of software options becomes available. In this case, mapping to a genomic reference that is evolutionarily diverged from the sequenced species may make accurate read mapping more difficult. For the diploid VA, mapping to homolog genes is expected, while for the tetraploid VC, reference genes may be mapped by reads originating from both homolog and homoeolog sequences. To account for sequence divergence, we compared results from five representative mapping software programs, run with either default settings or increasing mismatch tolerance (Fig. 6A, Supplementary Table S1). Overall, aligners behave similarly on both blueberry species. The programs that yield the most mapped reads are Stampy and GSNAP, both of which were designed to tolerate more sequence divergence during mapping, although only Stampy surpassed 5% mismatch rate (Fig. 6B). Bowtie2 and HISAT2 yielded the lowest mapping rates. The addition of relaxed conditions, despite modifying the percent of mismatches tolerated on alignments, did not have a significant effect on mapping results of GSNAP, Stampy, and STAR; it lowered the mapping rate for Bowtie2 and increased it for HISAT2, especially in VA. The effect of trimming was correlated with the number of available reads to be mapped; thus, Skewer improved mapping rates by 5%–11% compared to Trimmomatic (Supplementary Table S4). Finally, corrected reads, though not significant, promoted an increase in the mapping rate for all options, with 0.7% and 0.5% average increase in VA and VC and up to 2.5% in HISAT2 in VA.

It is desirable to utilize the maximum number of reads as possible in differential gene expression analysis, as increased depth of read counts leads to more sensitivity in statistical analysis. For example, more depth would increasingly allow detection of differences in lowly expressed genes or genes with small log fold changes in expression between treatments. To use this as a quality metric, we examined the successful conversion of raw reads to countable reads for each gene model using the software HTSeq. Starting from all mapping results, a read may not be converted to a countable read due to low-quality mapping, multiple alignments, or mapping to a genomic region without an annotation. The influence of each factor varies by mapping tool (Supplementary Fig. S2). The main cause of failed read conversion into counts was low quality of read alignment, found in Bowtie2, HISAT2, Stampy, and GSNAP, by decreasing magnitude. The second major factor that prevented counting was mapping within an intergenic region, which accounted for 5%–13% of mapped reads (Supplementary Figs. S2 and S3). Mapping to exonic features showed even larger variability, ranging from 57% displayed by Stampy to 80% by HISAT2, varying by mapping tool (Supplementary Fig. S3). In relation with mapping rate, these values indicate that both programs have similar mapping rates to exons, but Stampy is mapping more reads to non-exonic regions that may present higher sequence divergence. After collecting useful read counts, count rates to gene models were smaller than mapping rates by 14.2%, 10.9%, 7.5%, 15.7%, and 3.3% for Bowtie2, GSNAP, HISAT2, Stampy, and STAR, representing a loss of up to 45% of mapped reads for Bowtie2 and below 15% for STAR (Fig. 6A, right panels). Globally, modification of mismatch tolerance increased this loss in Bowtie2 and Stampy and reduced it in HISAT2. Read loss using Skewer compared to Trimmomatic was larger on GSNAP and Stampy and smaller on HISAT2 and Bowtie2. Interestingly, the rate of mapped reads not turned into counts in STAR was constant under the preprocessing and software options tested. After counting, count rates (Fig. 6A, lower values) displayed similar response to read processing as mapping rates discussed above, with GSNAP and Stampy showing equally high count rates.

**Figure 7:**
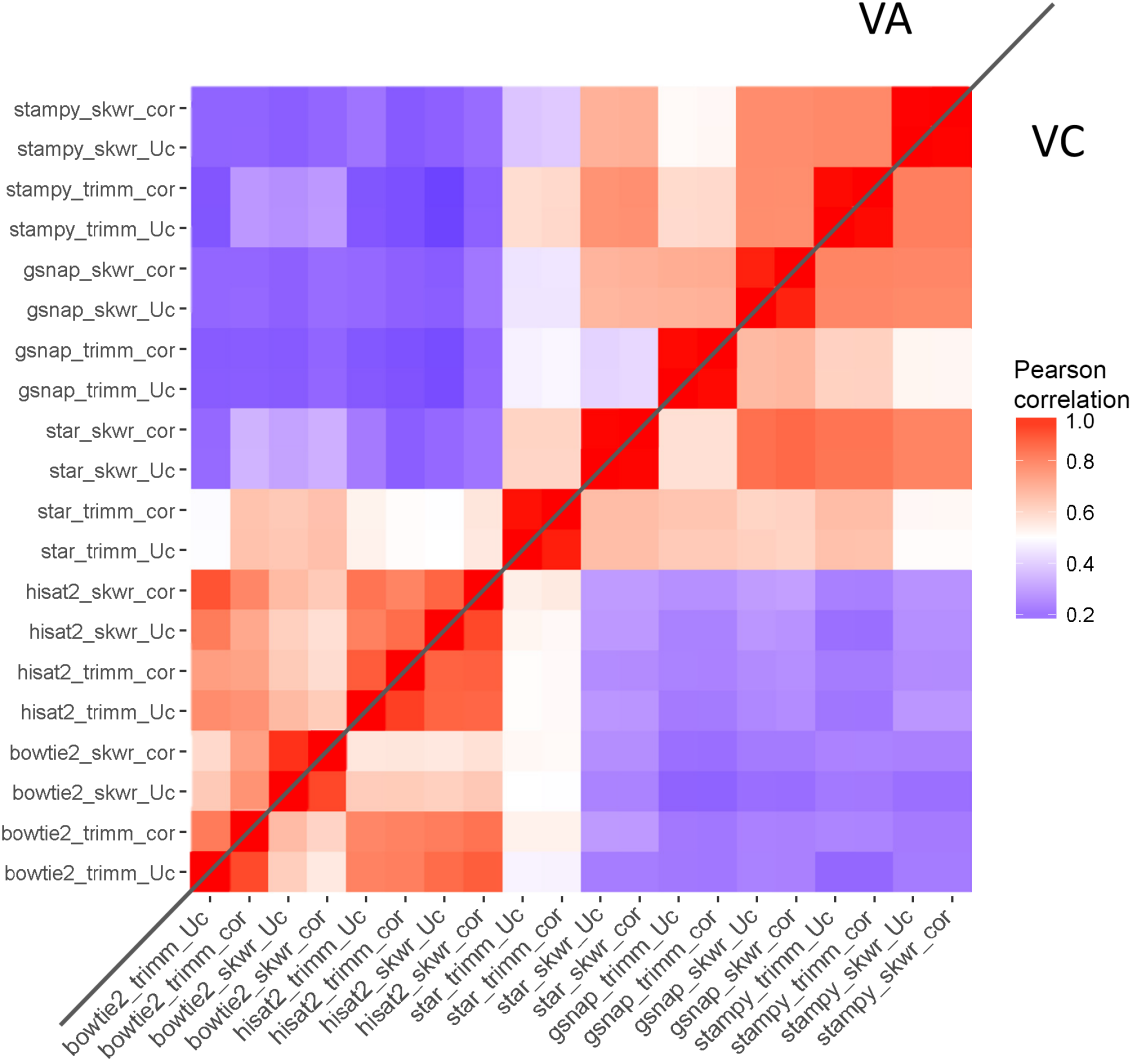
Correlation of gene count profiles after mapping to *V.corymbosum* genome. Values are mean of eight samples in either *V. arboreum* (VA, upper triangle) or V*. corymbosum* (VC, lower triangle). Each row/column corresponds to a unique combination of mapping software, trimming software, and error correction.

An important issue in science is reproducibility of results that, in the case of mapping results, can be reflected as similarity of gene count profiles, which ultimately determine genes that are differentially expressed. Correlation of counts was calculated across all blueberry samples comparing the 20 combinations of read processing and mapping software with default options (Fig. 7). Concomitant with their similarity on mapping results to the reference genome, VA and VC shared major correlation patterns between software programs, where two major groups are formed. This grouping is consistent with the algorithmic similarities of the software, i.e., one group is composed of Bowtie2 and HISAT2, which utilize a FM-index, and the second group includes GSNAP, Stampy and STAR, which use a combination of suffix array/hash table. Correlation was usually influenced by the trimming option, so that Skewer significantly improved correlation on GSNAP and STAR, Trimmomatic on Bowtie2 and Stampy, and HISAT2 was lightly affected by trimming. Interestingly, only Bowtie2 and HISAT2 responded to read correction, suggesting higher sensitivity to errors by the FM-index.

### Read mapping to *de novo* assemblies

The previous section focused on the effects of read correction, trimming, and alignment software on read mapping to a reference genome. Here, a similar analysis is performed though use of *de novo* assemblies from Trinity clustered with CD-HIT. To simplify the analysis, reads that underwent certain correction and trimming processing (e.g., samples with corrected reads trimmed with Skewer) were only mapped to the assemblies produced by reads with the same pre-processing. This method of *de novo* assembly followed by alignment is common for RNA-seq analysis when no reference genome is available and has advantages, including that mapping to transcript assemblies is usually contiguous, instead of spliced, and that assemblies are species specific, unlike a distant reference genome. All the aligners previously used for the genome alignment may also be used with transcriptomes. In addition, we incorporated the Salmon tool for transcript quantification, which is built solely for alignment of reads to a transcriptome.

**Figure 8:**
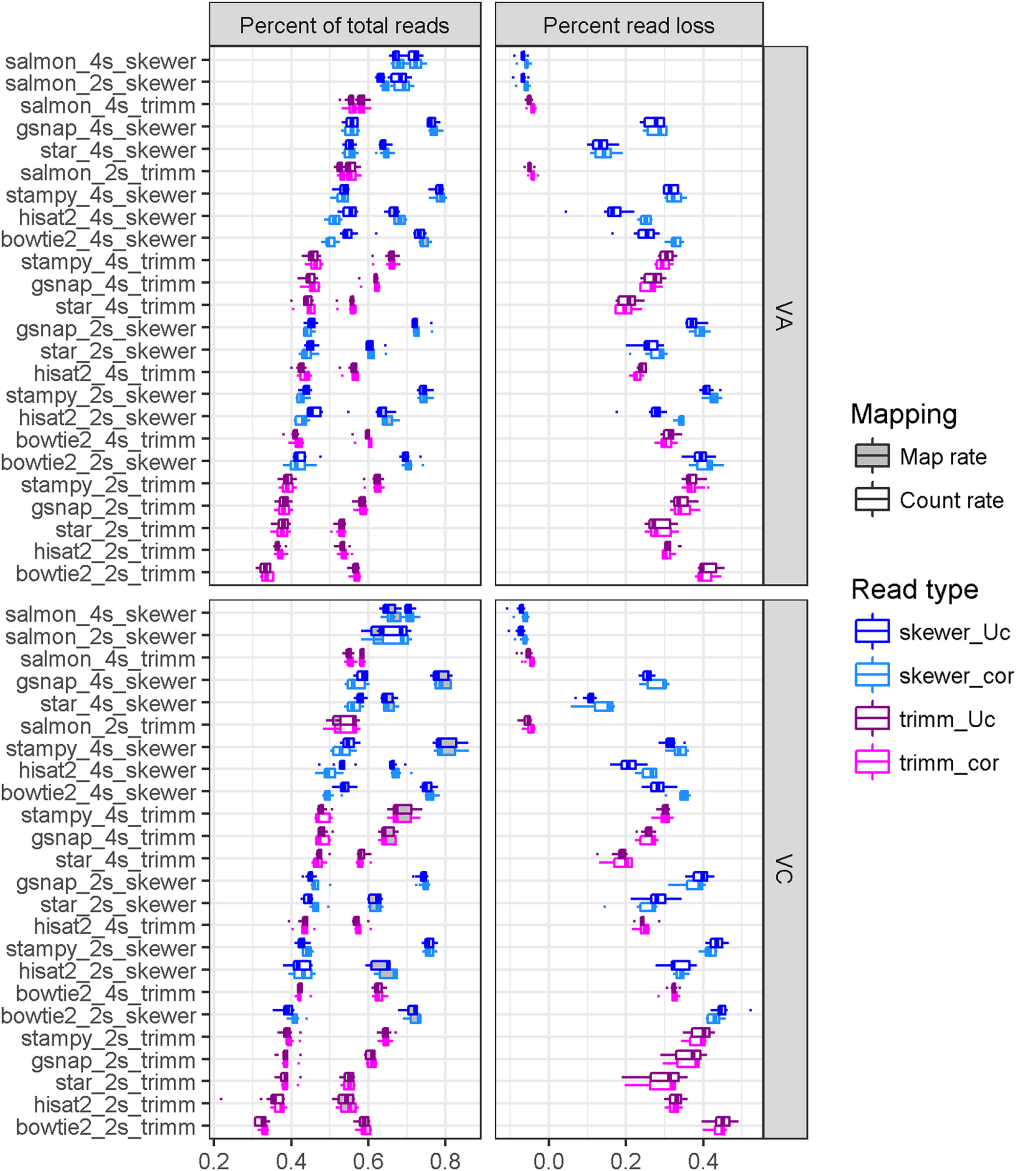
Read mapping to CD-HIT clustered *de novo* assemblies. Proportion of total mapped reads (left panels, gray boxes), converted to counts (left panels, white boxes), and percentage of the difference (right panels). Six mapping software programs were compared at default settings on assemblies made from four samples, produced either by two sets of two samples independently assembled (2s) and later merged or from the four samples assembled together (4s). Reads used (cor) or not (Uc) error correction and Trimmomatic (trimm) or Skewer trimming software.

Using *de novo* assemblies as the reference, mapping performance of the five aligners showed lower variability by condition (trimming and type of assembly) compared to mapping to the genome, with Stampy and GSNAP again as best performers (Fig. 8). The mapping profile was similar for both species, with higher mapping rates for VC than VA by 1.4% using Skewer and 2.5% using Trimmomatic, except for Salmon. Also, 4s assemblies had consistently better mapping rates than 2s assemblies, with improvements for Skewer/Trimmomatic of 3.7/3.0% in VA and 3.8/3.4% in VC. Examining only the effect of trimming, yield is likewise correlated with the number of reads available for mapping, so that Skewer had on average 12.5% more reads mapped than Trimmomatic. Finally, error correction of reads did not have a significant effect on read mapping. Examining conversion of raw reads to countable reads, 30%–45% and 22%–30% of mapped reads in 2s and 4s assemblies were not able to be turned into counts, with higher values on 2s assemblies than 4s ones (Fig. 8, right panels). For Bowtie2 and Stampy, the major cause of read loss was low-quality alignments, while for GSNAP, HISAT2, and STAR, most of the dropped reads were multimapped (Supplementary Fig. S4). Read counts further reduced variability across programs and intensified the difference between mapping to 4s compared to 2s assemblies, increasing by 9.1/6.1% in VA and 9.8/7.9% in VC for Skewer/Trimmomatic, respectively. The difference between using Skewer or Trimmomatic was reduced to an average of 9%. The different results yielded by Salmon reflect its different algorithm, which performs pseudo-mapping to estimate abundance but does not report mapping results in a format suitable to do quality assessment of alignments. The consequence is that Salmon has an artificially higher estimated count rate than reads mapped, and since no reads are filtered out for quality score, Salmon has higher count rates than other approaches.

**Figure 9:**
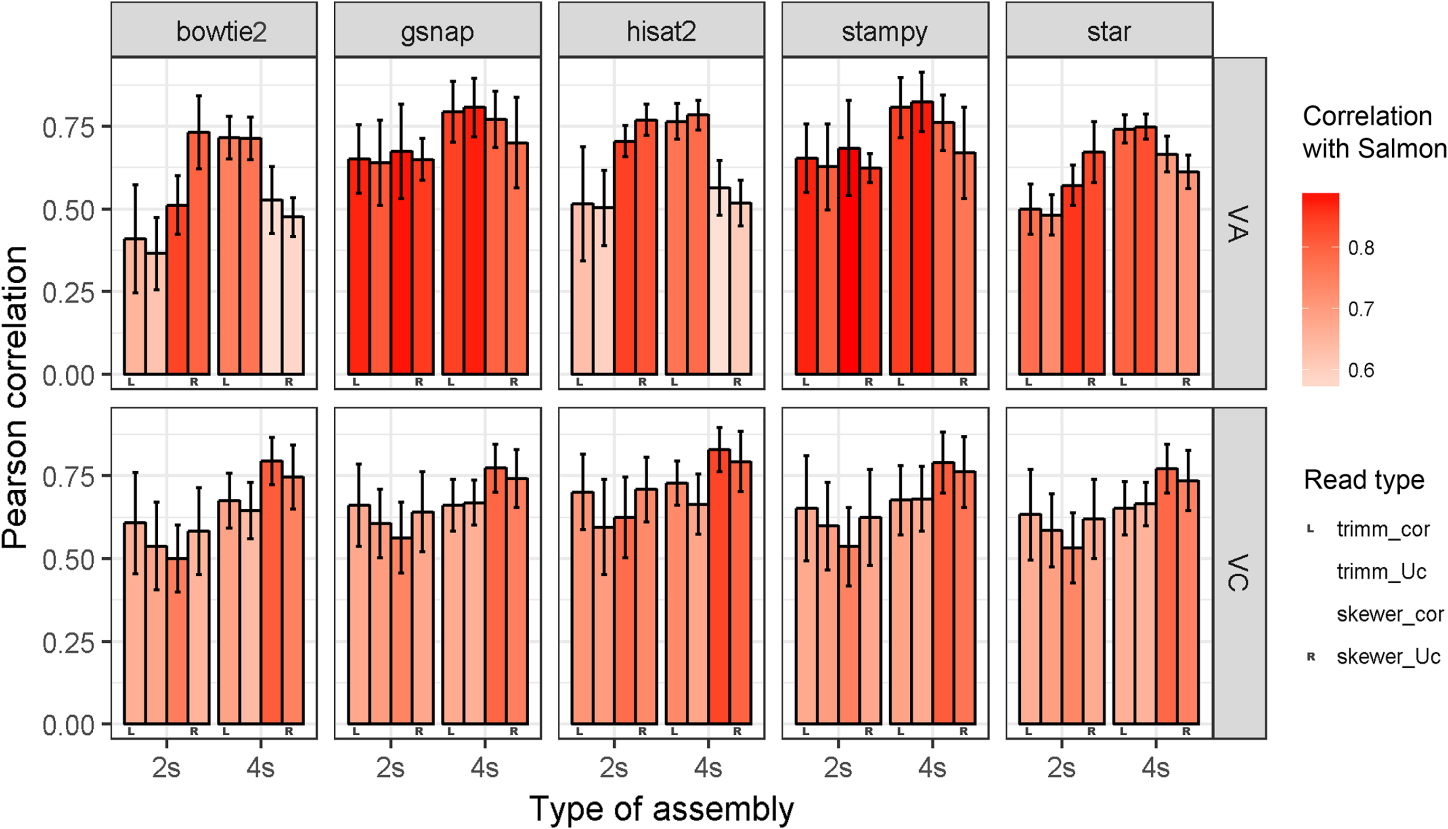
Correlation of gene count profiles obtained with *de novo* assemblies and the reference genome. Counts of transcripts aligned to the same reference gene model were added and re-annotated as that gene model. Correlation was calculated on the common set of gene models with non-zero counts on both reference and assemblies by mapping software and read pre-processing (error correction and trimming). Uc stands for uncorrected, cor for corrected, trimm for Trimmomatic. Color indicates mean correlation of reference counts with Salmon, a transcript-specific quantification tool. Values are mean ± standard deviation of eight samples.

In the case of mapping to a *de novo* assembly, calculation of a correlation of mapping results is not directly due to each assembly having their own set of transcripts. Hence, rather than program-to-program correlation, which is shown in the previous section, reference-to-assembly count profiles were compared (Fig. 9). To do this, the reference gene model gene space was used for such comparison. New count profiles for assembly mapping results were obtained by adding counts of all transcripts mapped to each single reference gene model. Then, they were compared to results with the reference genome by same read pre-processing and mapping software. Utilization of the reference genome from diploid VC, though useful for a shared gene set to compare, has the inconvenience of not representing species-specific transcripts (blue bars in Fig. 4). VA is a sister species but is also a diploid, so one-to-one homology may be expected. However, tetraploid VC assemblies not only contain a larger proportion of transcripts that do not match the genome but also splice isoforms, and lowly diverged homoeolog sequences are expected to map to the same gene models. Likewise, balancing this effect, reads originated from transcripts sharing sequence similarity are expected to map to the same gene model on the reference genome.

The highest assembly-to-genome correlation values are obtained on the diploid VA, which reach 75% on all programs (Fig. 9). However, the best-performing program differs by species: GSNAP and Stampy for VA and Bowtie2 and HISAT2 for VC. For both species, results with the larger 4s assemblies are better correlated to the genome than the 2s assemblies. Overall, the preference for trimming software, if any, is opposite by species; Skewer and Trimmomatic improves 2s and 4s assemblies in VA, respectively, and Skewer improves 4s assemblies in VC. These differences caused by read processing are more prominent on 4s assemblies, while on 2s assemblies they induce significant changes on VA with Bowtie2, HISAT2, and STAR. This suggests that stricter trimming in the distant VA may help mapping accuracy on the diploid VC genome, especially with Bowtie2 and HISAT2 4s, while gentle trimming in the tetraploid VC may help by either better assembly of transcripts or read mapping. Salmon results correlate well with the different aligners in VA, especially GSNAP and Stampy (Fig. 9, bar colors), while the tetraploid VC has overall poorly comparable results. This suggests that Salmon transcript quantification may be better suited for less complex genomes.

## Discussion

RNA-seq is an affordable and versatile tool to analyze transcriptomes of any species. Depending on the available resources, it can be guided by a reference genome or by building custom assemblies that will reflect the transcripts present in the samples. However, many confounders make the analysis less straightforward than simply trimming adapters, assembling reads as needed, and mapping to a reference. Some of these confounders are common for any RNA-seq data analysis, such as sequencing errors, repetitive sequences, natural heterozygosity, and variants, while the analysis of a species other than the reference has additional sequence variation and, in the case of a polyploid, gene redundancy. Thus, we explored the repercussions of various informatic choices on the final gene expression profiles.

Illumina short-read sequencing, though very accurate, is not exempt of sequencing errors. One strategy to deal with low-quality nucleotides aims to correct reads, usually by replacing poorly represented *k*-mers with similar ones of higher frequency patterns [38]. Effectivity of error correction on RNA-seq data is lower than on genomic data due to differences in expression level and splicing and is less dependent on the organism of study [8]. Despite sequencing errors of Illumina technology occurring at a reported average rate of only 0.1% bases [6], Rcorrector modified 0.7% bases in both species. While error correction tools can reduce sequencing errors, they can also introduce new errors at a variable rate, especially for complex datasets [38]. For a complex gene family or when examining a polyploid, this could be a significant problem, with some reads converted to the sequence of a close homolog, leading to incorrect mapping and/or misassembly. However, in this study, read correction did not reflect significant variation in overall mapping success. It induced a small amount of variation only on those aligners that use an FM-index, Bowtie2 and HISAT2, and thus require perfect matching for seeding an alignment. Read correction was more important for assemblies that exhibited larger changes depending on correction state, such as larger number of transcripts, higher Detonate scores, or higher completeness when using corrected reads in most cases, especially with SOAPdenovo-Trans. Previous research also demonstrated that error correction impacts genome assembly [38].

Trimming is required to, at the least, remove sequencing adapters and often also addresses short reads and low-quality bases. The broadly used tool Trimmomatic implements strict trimming based on sequencing base quality, where trimming removes low-quality bases that could lead to complex or incorrect de Bruijn graphs, but also reduces read length, which may have a negative impact on coverage bias [38]. Skewer takes a much less stringent trimming approach. The extent to which trimming of low-quality bases is beneficial for downstream analyses was explored for DNA-seq [42], suggesting a positive effect on genome assembly despite increased fragmentation and a tradeoff between accuracy and recall of assemblies. In our experiments, similar effects derived from trimming were shown on both the diploid or tetraploid species, especially with Trans-ABySS or Trinity. We found that Skewer (soft trimming) usually led to more complete assemblies at the expense of a larger amount of noncoding transcripts, while Trimmomatic (i.e., strict quality trimming) improved support from input reads and consistency of GC content across assemblers. In Trinity clusters, Trimmomatic also reduced fragmentation of assemblies and enhanced biological consistency of clustering. In mapping experiments, higher-quality reads are mapped at a larger relative proportion; however, this is at the expense of losing many reads at the trimming stage, many of which may have been successfully mapped downstream. Nonetheless, both options can lead to comparable expression profiles, usually if mapping tools can deal with bases of lower quality [42].

There are cases where transcriptome assemblies are required, such as absence of a suitable reference genome or discovery of novel isoforms. For transcriptome assembly with samples derived from various conditions, two approaches are common: one in which the samples are pooled into a single run [40, 41] and one in which samples are assembled independently [43–45]. The major interest is to obtain transcripts that are specific to each sample, and combination of reads is a potential source for mis-assembly or formation of chimeras. In this respect, we found that transcripts from separate samples had significantly higher assembly base coverage (transcript bases mapped to the reference genome), although the combined samples had better genome base coverage (reference genome bases covered by transcripts). However, merging individual assemblies generates high redundancy. This effect was studied in wheat, reporting that redundant merged assemblies showed improved read mappability with Trinity but lower with Trans-ABySS, but also had less continuity than assemblies from pooled samples, and their quality decreased after clustering [43]. We found improved read support on merged assemblies for the three assemblers, but lower mean transcript size and completeness. A strong reverse correlation between fragmentation of genes and assembled reads was also found, supporting that sequencing depth is beneficial to the recovery of full-length transcripts [13, 15, 20]. General conclusions apply to both the diploid and the tetraploid species, although the polyploid had proportional increased duplication rate and exhibited a larger species-specific proportion of transcripts. On the other hand, proper clustering in polyploids is difficult, not unexpectedly, as it must handle isoforms of genes as well as homoeologs. This is reflected by the outcomes of the clustering methods utilized, where aggressive reduction of redundancy also leads to loss of completeness, though to a lesser extent than sequencing depth.

Scientists examining organisms without a specific reference face the decision of whether to use the reference genome of a close organism or to build a custom *de novo* assembly. Mapping to a distant reference has disadvantages, including sequence divergence at the nucleotide level and also larger structural divergence, where genes may be missing or duplicated between the species. From our species studied, it would be expected for the distant diploid VA to have undergone greater sequence divergence than the tetraploid relative of the reference diploid VC, in which divergence would be driven by diversifying subgenomes. Mapping results to the reference genome reflect this issue, where mapping tools that have greater sensitivity to align divergent sequences, such as Stampy, GSNAP, and STAR, improve mapping results of VA compared to VC, while HISAT2 and Bowtie2, which require an exact match to seed, perform better in VC than VA. Regardless of the species, we found GSNAP and Stampy to yield the highest performances on the reference genome, probably due to their ability to align divergent sequences even at default settings. On the second mapping strategy, utilizing specific assemblies allowed much higher mapping rates compared to the reference, concordant with the high proportion of transcripts not represented on the genome that are now available to be mapped. Both species displayed comparable results when mapping to an assembly, slightly better on the tetraploid VC than on the diploid VA except with Salmon, probably due to the better completeness of the VC transcriptomes. In addition to higher mapping rates, specific biological information may be present on transcripts not represented in the genome, from which 64.5% had a predicted cds, gaining insight into the processes under study. Nonetheless, in addition the divergence with the reference genome, using assemblies can give similar results at 75% correlation; awareness of mismatches also played a role, improving correlations of VA with GSNAP and Stampy and of VC with HISAT2.

In conclusion, using a reference genome with either a distant diploid species or a polyploid relative can give reliable results, simplifying the RNA-seq analysis by skipping *de novo* assembly and associated steps. In the present work, we expanded many possibilities from read processing to gene counting, providing a complete overview on how each of the tested options impacts gene expression profiles. On both species studied, the pipeline that yielded high outcome with comparable results using either a reference genome or a transcriptome assembly used trimming with Skewer, a combination of multiple samples for improved assembly quality, and Stampy or GSNAP for short-read mapping. This pipeline was oriented to maximize the recovery of information from RNA-seq reads, working with the specific case where samples and reference genome are not from the same organism. While we suggest that this strategy can be extrapolated to other systems, our study also highlights the many downstream impacts software analysis decisions can have on results. For scientists faced with complex RNA-seq analysis projects, testing of different software packages to examine and optimize results can be beneficial.

## Methods

The following methods include a brief summary of the tools that were used in this work. For detailed descriptions of the algorithms, please refer to original publications or websites.

### Sequencing of RNA-seq reads of blueberry roots

Preparation of RNA-seq libraries from root tissue of diploid *V. arboreum* cultivar FL148 and tetraploid *V. corymbosum* “Emerald” blueberry species are previously described [25] and available in NCBI as bioproject PRJNA353989. Briefly, eight plants per species were acclimated to growth in hydroponic systems at either pH 4.5 or pH 6.5 for 8 weeks, after which roots were collected and flash frozen. RNA was extracted and prepared for sequencing of 100 base-pair (bp) paired-end reads on a HiSeq 2000 system (Illumina, CA).

### Error correction and trimming of RNA-seq reads

Rcorrector (*RNA-Seq error CORRECTOR*) [8] is a *k*-mer-based error correction method that uses a de Bruijn graph to represent trusted *k*-mers, a method similar to that used on *de novo* assembly. Rcorrector v1.0.2 was applied to raw reads with default parameters. Then, sets of corrected and uncorrected reads were trimmed for removal of Illumina adapter sequences using either Trimmomatic v0.35 [36], specifying parameters “SLIDINGWINDOW:4:15” and minimum read length of 30 bp, or Skewer v0.2.2 [37], with the same minimum length cutoff. Trimmomatic searches adapters by finding an approximate match and aligning using a *seed and extend* approach [46], both for regular and “adapter read-through” scenarios. Illumina quality scores of bases are used to determine cut points, discarding the 3’ end of the read. Skewer uses a novel *bit-masked k-difference matching* dynamic programming algorithm, which uses a variation of the *Smith-Waterman* [47] algorithm to search substrings and solve the *k-difference problem* and an extended *bit-vector algorithm* [48] to handle base-call quality values. Skewer can remove low-quality bases on both 5’ and 3’ read ends and is considerably faster than Trimmomatic. FastQC v0.11.4 [49] was used for quality assessment of reads. From each original read file (VA control, VA treatment, VC control, VC treatment), the combination of error correction and trimming generated four new sets of trimmed reads to be utilized in downstream processes: reads processed by Rcorrector and Trimmomatic, reads processed by Rcorrector and Skewer, reads processed by Trimmomatic only, and reads processed by Skewer only.

### *de novo* transcriptome assembly and redundancy reduction

Each of the four processed read sets was used for transcriptome *de novo* assembly, independently for each blueberry species, using Trinity 2.2.0 [14], Trans-ABySS v1.5.5 [16], and SOAPdenovo-Trans v1.03 [17], with *k*-mer = 25 and filtering for a minimum contig length of 200 bp. Environmental stress is expected to alter the transcripts present in the cells as well as transcript splicing patterns. To include this source of variability, two commonly used approaches were considered: assemble control and treated samples independently and concatenate results after assembly and combine two control and two treated samples in the same assembly run. Together, 12 Trinity assemblies for each species were generated (Supplementary Fig. S1). The next step consisted of removing redundant transcripts from assemblies using either CD-HIT v4.6.6 [18] at 95% identity or RapClust [50]. CD-HIT sorts all transcripts by length and attempts to consecutively cluster smaller sequences to longer representative ones, getting classified as redundant or representative based on sequence similarity; the result included a reduced transcript set consisting of one sequence per cluster. On the other hand, RapClust was developed to group assemblies using information from multimapper paired-ended reads, thus requiring input from Salmon [51] aligner. From the clustering information after RapClust, reduced transcriptomes were obtained after selection of the longest transcript per cluster. This step generated 16 clustered assemblies for each species (Supplementary Fig. S1).

### Quality assessment and functional annotation of assemblies

Transcriptome *de novo* and clustered assemblies were assessed for quality with DETONATE 1.11 [22] to calculate a score weighed with the reads used to generate each assembly, Transrate 1.0.3 [12] to get basic metrics, and BUSCO v2.0 [21] for completeness assessment. To compare the Trinity *de novo* assemblies to the genome, reduced assemblies were mapped to the diploid blueberry reference genome [35] with gmap version 2017-05-08 [52]. Base coverage was calculated on uniquely mapping transcripts using coverageBed from the BEDTools suite version 2.26 [53].

Biological consistency of clustering results was evaluated with a custom Jaccard similarity score based on the method described in [54] using the BUSCO annotation results on Trinity assemblies. Each cluster received an individual score calculated as the number of transcripts with the same BUSCO annotation within the cluster divided by the total number of transcripts with that BUSCO annotation plus the number of transcripts in the cluster that did not share that annotation. The statistic is based on amount of the intersection divided by amount of union where the two sets are all the transcripts sharing a BUSCO annotation and all the transcripts in a cluster. If multiple annotations were present in a cluster, the maximum score was selected for that cluster. The result is a value between 0, indicating low co-annotation of transcripts, and 1, indicating perfect clustering of co-annotated transcripts. Clusters with a single transcript were omitted.

Putative ORFs were predicted for each Trinity clustered assembly with TransDecoder v3.0.0 [55], software that incorporates results from blast [56] and Pfam [57] homology searches to select best ORF candidates. First, candidate cds encoding at least 50 amino-acid-long peptides were extracted from transcripts. Then, these were searched with blast against the plant TrEMBL protein database (evalue < 10e-5) and with HMMER 3.1b2 [58] against Pfam. Finally, a single putative ORF was selected for each transcript when possible.

### Read mapping

The four sets of processed RNA-seq reads from VA and VC were mapped to either the draft reference genome for diploid VC or Trinity *de novo* assemblies clustered with CD-HIT, using STAR 2.5.0, Stampy v1.0.28, GSNAP 2016-11-07, Bowtie2 2.2.8, and HISAT2 2.0.4. Software options were modified or not when mapping to the reference genome to increase mismatch tolerance. Salmon v0.7.2 [51], which uses quasi-mapping with a two-phase inference procedure, was specifically used on transcriptomes. Mapping metrics were collected using picard tools v2.1.0 [59] and RNA-SeQC v1.1.8 [60]. Finally, counts were obtained using HTSeq-count Version 0.6.1p1 [61].

Short-read aligners can be classified by algorithmic approach as not splice-aware (Bowtie2, Stampy) or splice-aware (HISAT2, STAR, GSNAP), or by their use of an uncompressed index, such as hash table, or compressed indexes, such as suffix arrays, Burrows-Wheeler transform (BWT) methods, and Full-text index in Minute space (FM-index). Bowtie2 [62] uses an algorithm based on the BWT and the FM-index, which extracts seed substrings from reads, finds exact alignments with the FM-index, and extends with gapped dynamic algorithms such as *Needleman-Wunsch* (global alignment) or *Smith-Waterman* (local alignment). Stampy [63] uses a hash table with locations of 15-mers in the genome used to search every overlapping 15-mer in the reads. Those that pass neighborhood similarity filtering are extended with *Needleman-Wunsch*. GSNAP (Genomic Short-read Nucleotide Alignment Program) [52] combines a set of algorithms to improve accuracy of alignment, using either hash tables or enhanced suffix arrays. Sequentially after failure of previous methods, GSNAP searches for a single continuous match, applies segment combination procedures, or employs its complete set analysis to allow for larger mismatch proportion. STAR (Spliced Transcripts Alignment to a Reference) software [64] is based on an algorithm that uses “sequential maximum mappable seed search in uncompressed suffix arrays followed by seed clustering and stitching procedure.” After stitching of seeds, the unmapped portions of the reads can be extended with *Needleman-Wunsch* algorithm. HISAT2 (Hierarchical Indexing for Spliced Alignment of Transcripts) [65] is based on the BWT and the FM-index, with operation methods adapted from Bowtie2. In addition to the global FM-index, the genome is divided into a large set of small FM-indexes. Read strings are first mapped to the global FM-index to find candidate locations, and the remaining bases are aligned with a local index, combining extension by direct comparison of sequences and further local index search of unaligned fragments.

## Availability of supporting data

The RNA-seq data (SRA496374) was deposited in the Sequence Read Archive database from the publicly available repository NCBI, https://www.ncbi.nlm.nih.gov/sra/?term=SRA496374. Further supporting data are available in the *GigaScience* repository, GigaDB [66].

## Additional files

**Figure S1**. Diagram representing the *de novo* assembly strategies, run independently for each *Vaccinium* species. The set of control and treatment reads produced by different correction and trimming strategies were used as input. The control read files were assembled (A) independently as were the treatment read files (B). From here, each set of control sample transcripts was combined with the treatment sample transcripts (i.e., the Skewer corrected control transcripts were merged with the Skewer corrected treatment transcripts, the Trimmomatic uncorrected control transcripts were merged with the Trimmomatic uncorrected treatment transcripts, etc.) (C). These merged transcript sets were then clustered with either CD-HIT (D) or RapClust (E). This results in eight clustered assemblies. A second assembly strategy merged the control and treatment reads prior to assembly (F). These sets of transcripts were also clustered with either CD-HIT (G) or RapClust (H), also resulting in another set of eight clustered assemblies.(.jpg).

**Figure S2**. Subdivision in categories of reads mapped to the reference genome performed by HTSeq. Except in the case of STAR, which does not report not mapped reads, height of bars up to red resembles the number of trimmed reads. Options are ordered by correction state, mismatch tolerance options and trimming software. (.tiff).

**Figure S3**. Mapping results to the reference genome categorized by overlapping gene feature. (.tiff).

**Figure S4**. Subdivision in categories of reads mapped to *de novo* assemblies performed by HTSeq. In specific cases with HISAT2 and STAR, multiple aligned reads are counted multiple times, overestimating the total number of reads. Options are ordered by correction state, trimming software and type of assembly. (**.pdf**).

**Table S1**. Description of main algorithms used on this work. Brief algorithmic explanations, software claims and major findings are included for programs tested at (A) pre-processing of RNA-Seq reads, (B) *de novo* assembly of transcriptomes and redundancy reduction by clustering, and (C) mapping of short reads to both blueberry reference genome and Trinity assemblies clustered with CD-HIT. BWT, Burrows-Wheeler Transform; FM-index, Full-text index in Minute space. (.xlsx).

**Table S2**. Variation in number and length of reads after pre-processing. Number of reads before and after trimming with either Skewer or Trimmomatic and using (cor) or not (Uc) error correction. Last column indicate average length of reads after trimming the 101-bp raw reads. Values are mean ± sd of 8 samples. (.xlsx).

**Table S3**. Mapping and annotation metrics of Trinity clustered assemblies to *V. corymbosum* reference genome. Transcripts mapped either uniquely to the genome (uniq), to multiple locations (mult), with translocations (transloc) or did not map (out). Subdivision based on annotation includes “All mapping transcripts” (map), “Mapping transcripts with CDS” (cds) and “CDS with blast hit” (blast). (.txt)

**Table S4**. Read mapping rates. Proportion of reads mapped from each combination of error correction, trimming software, mismatch tolerance or assembly samples, when appropriate, to either the reference genome or *de novo* assemblies after clustering with CD-HIT. (.xslx).

## Abbreviations

2s: two-sample; 4s: four-sample; bp: base-pair; BUSCO: Benchmarking Universal Single-Copy Orthologues; BWT: Burrows-Wheeler transform; cds: coding DNA sequence; cor: Use of error corrected reads by Rcorrector; FM-index: Full-text index in Minute space; GSNAP: Genomic Short-read Nucleotide Alignment Program; NCBI: National Center for Biotechnology Information; ORF: open reading frame; RNA-seq: RNA sequencing; Uc: Use of not corrected (or uncorrected) reads; VA: *Vaccinium arboreum*; VC: *Vaccinium corymbosum*.

## Competing interests

The authors declare that they have no competing interests.

## Funding

This research was supported by the National Institute of Food and Agriculture, US Department of Agriculture (award 2009–02533) and the Thad Cochran Southern Horticultural Laboratory, US Department of Agriculture Agricultural Research Service (under Non-Assistance Cooperative Agreement agreement 58–6062–5-004).

## Author contributions

G.N., J.O., and T.R. prepared the biological material and collected sequencing data. M.S. and M.P.M. conceived and designed the analysis workflow, analyzed the results and prepared figures, and contributed to the writing of the manuscript. M.P.M. performed computational analysis of the data. All authors read and approved the final manuscript.

## Supplementary Material

GIGA-D-18-00140_Original_submission.pdf

GIGA-D-18-00140_Revision_1.pdf

Response_to_Reviewer_Comments_Original_Submission.pdf

Reviewer_1_Report_(Original_Submission) -- Ricardo Ramirez-Gonzalez 5/22/2018 Reviewed

Reviewer_1_Report_revision_1 -- Ricardo Ramirez-Gonzalez 1/10/2018 Reviewed

Reviewer_2_Report_(Original_Submission) -- Ruibang Luo 2/6/2018 Reviewed

Reviewer_2_Report_Revision_1 -- Ruibang Luo 9/23/2018 Reviewed

Supplement Files
