# Supplementary material for: Comprehensive evaluation of RNA-seq analysis pipelines in diploid and polyploid species
Source: Gigascience. 2018 Nov 10;7(12):giy132. doi: 10.1093/gigascience/giy132 (PMC6275443; doi:10.1093/gigascience/giy132)
Supplement: GIGA-D-18-00140_Original_submission.pdf [file giy132_giga-d-18-00140_original_submission.pdf]

GigaScience

Comprehensive evaluation of RNA-Seq analysis pipelines in diploid and polyploid species

--Manuscript Draft--

Manuscript Number:	GIGA-D-18-00140
Full Title:	Comprehensive evaluation of RNA-Seq analysis pipelines in diploid and polyploid species
Article Type:	Research
Funding Information:	National Institute of Food and Agriculture (2009–02533) — Dr. Margaret Staton<br>Agricultural Research Service (58–6062–5-004) — Dr. Margaret Staton
Abstract:	Background<br><br>The usual analysis of RNA-Seq reads is based on an existing reference genome and annotated gene models. However, when a reference for the sequenced species is not available, alternatives include using a reference genome from a related species or reconstructing transcript sequences with de novo assembly. In addition, researchers are faced with many options for RNA-Seq data processing and limited information on how their decisions will impact the final outcome. Using both a diploid and polyploid species with a distant reference genome, we have tested the influence of different tools at various steps of a typical RNA-Seq analysis workflow on the recovery of useful processed data available for downstream analysis.<br><br>Findings<br><br>At the preprocessing step, we found error correction has a strong influence on de novo assembly but not on mapping results. After trimming, a greater percentage of reads were able to be used in downstream analysis by selecting gentle quality trimming performed with Skewer instead of strict quality trimming with Trimmomatic. This availability of reads correlated with size, quality and completeness of de novo assemblies, and number of mapped reads. When selecting a reference genome from a related species to map reads, outcome was significantly improved when using mapping software tolerant of greater sequence divergence, such as Stampy or GSNAP.<br><br>Conclusions<br><br>The selection of bioinformatic software tools for RNA-Seq data analysis can maximize quality parameters on de novo assemblies and availability of reads in downstream analysis.
Corresponding Author:	Margaret E Staton, Ph.D.<br>University of Tennessee Knoxville<br>Knoxville, TN UNITED STATES
Corresponding Author Secondary Information:	
Corresponding Author's Institution:	University of Tennessee Knoxville
Corresponding Author's Secondary Institution:	
First Author:	Miriam Payá-Milans
First Author Secondary Information:	
Order of Authors:	Miriam Payá-Milans<br>James W. Olmstead

Powered by Editorial Manager® and ProduXion Manager® from Aries Systems Corporation

	Gerardo Nunez
	Timothy A. Rinehart
	Margaret Staton
**Order of Authors Secondary Information:**	
**Additional Information:**	
**Question**	**Response**
Are you submitting this manuscript to a special series or article collection?	No
**Experimental design and statistics**<br><br>Full details of the experimental design and statistical methods used should be given in the Methods section, as detailed in our Minimum Standards Reporting Checklist. Information essential to interpreting the data presented should be made available in the figure legends.<br><br>Have you included all the information requested in your manuscript?	Yes
**Resources**<br><br>A description of all resources used, including antibodies, cell lines, animals and software tools, with enough information to allow them to be uniquely identified, should be included in the Methods section. Authors are strongly encouraged to cite Research Resource Identifiers (RRIDs) for antibodies, model organisms and tools, where possible.<br><br>Have you included the information requested as detailed in our Minimum Standards Reporting Checklist?	Yes
**Availability of data and materials**<br><br>All datasets and code on which the conclusions of the paper rely must be either included in your submission or deposited in publicly available repositories (where available and ethically appropriate), referencing such data using a unique identifier in the references and in the "Availability of Data and Materials" section of your manuscript.<br><br>Have you have met the above requirement as detailed in our Minimum	Yes

Standards Reporting Checklist?	

Powered by Editorial Manager® and ProduXion Manager® from Aries Systems Corporation

Click here to download Manuscript workflow paper v4 MS2 upload version.docx

Comprehensive evaluation of RNA-Seq analysis pipelines in diploid and polyploid species

Miriam Payá-Milans[1], James W. Olmstead[2], Gerardo Nunez[2], Timothy A. Rinehart[3,4], Margaret Staton[1*]

Affiliations for authors:

[1] Department of Entomology and Plant Pathology, University of Tennessee

[2] Horticultural Sciences Department, University of Florida

[3] Thad Cochran Southern Horticultural Laboratory, USDA-Agricultural Research Service

[4] Crop Production and Protection, USDA-Agricultural Research Service

emails:

Miriam Payá-Milans (MPM): mmilans@utk.edu

James W. Olmstead (JO): james.olmstead@driscolls.com

Gerardo Nunez (GN): g.nunez@ufl.edu

Timothy A. Rinehart (TR): tim.rinehart@ars.usda.gov

Margaret Staton (MS): mstaton1@utk.edu

*Corresponding author:

E-mail: mstaton1@utk.edu

Abstract

Background: The usual analysis of RNA-Seq reads is based on an existing reference genome and annotated gene models. However, when a reference for the sequenced species is not available, alternatives include using a reference genome from a related species or reconstructing transcript sequences with *de novo* assembly. In addition, researchers are faced with many options for RNA-Seq data processing and limited information on how their decisions will impact the final outcome. Using both a diploid and polyploid species with a distant reference genome, we have tested the influence of different tools at various steps of a typical RNA-Seq analysis workflow on the recovery of useful processed data available for downstream analysis.

Findings: At the preprocessing step, we found error correction has a strong influence on *de novo* assembly but not on mapping results. After trimming, a greater percentage of reads were able to be used in downstream analysis by selecting gentle quality trimming performed with Skewer instead of strict quality trimming with Trimmomatic. This availability of reads correlated with size, quality and completeness of *de novo* assemblies, and number of mapped reads. When selecting a reference genome from a related species to map reads, outcome was significantly improved when using mapping software tolerant of greater sequence divergence, such as Stampy or GSNAP.

Conclusions: The selection of bioinformatic software tools for RNA-Seq data analysis can maximize quality parameters on *de novo* assemblies and availability of reads in downstream analysis.

Keywords: RNA-Seq, pipeline, polyploid, correction, trimming, assembly, clustering, reference genome, mapping

Background

Bioinformatics is a field under constant expansion with regular advances in the development of software and algorithms. This requires researchers to continuously evaluate available software tools and approaches to maximize accuracy of experimental outcomes [1]. However, the majority of the relevant studies comparing bioinformatic tools for RNA-Seq data focus on straightforward scenarios with diploid eukaryotes with an available reference genome [2-5]. The implications of data analysis decisions are less clearly understood in situations where, for example, the species of interest is a polyploid or the species of interest does not have a reference genome but a reference genome is available from a sister clade. This study aims to explore RNA-Seq data analysis from this scenario, where the main steps are read trimming, either mapping to a related species reference genome (from here on referred to as a "distant reference") or to a *de novo* transcriptome assembly, and read quantification by gene or transcript (Figure 1). Moreover, this study compares decisions along the RNA-Seq analysis steps of a workflow, examining all permutations of those decisions from the beginning to the end of the pipeline.

Figure 1. Schematic view of the RNA-Seq pipeline. Uc stands for uncorrected, trimm for Trimmomatic, Cor for corrected.

From the many next generation sequencing platforms that generate RNA-Seq data, Illumina has had the greatest success, yielding high quality reads at a reasonable price and read length increasing with new generations of instruments [6]. From the raw reads, numerous informatic analysis decisions must be made to derive meaningful biological data, starting with any preprocessing of the reads. Despite the usually high accuracy of Illumina reads (0.1% error rate), error correction is a method with potential to improve the quality of read alignment and *de novo* assembly [7]. Before sequencing, adapters are incorporated to both ends of each sequence. Trimming of bases originating from these adapters is required, but the merit of aggressive versus gentle trimming of lower quality bases, which modifies the final amount of data, is still being explored [8].

After preprocessing, if a reference genome is available, RNA-Seq reads may be used to call variants or determine differentially expressed genes; on the contrary, *de novo* assembly may be used to reconstruct transcripts to do such analyses [9]. *De novo* transcriptome assembly in plants is complex due to the sequence similarity of transcripts that are isoforms, paralogs, orthologs and, in the case of polyploids, homeologs. Moreover, in transcriptomes of plants under environmental stress, alternative splicing is even more prevalent [10]. This complexity leads to imperfect assemblies, with a portion of assembled transcripts affected by artifacts, which include hybrid assembly of gene families, transcript fusion (chimerism), insertions in contigs, and structural abnormalities such as incompleteness, fragmentation, and local misassembly of contigs [11]. From the many assemblers developed to use with short reads, Trinity [12] is commonly selected and has good performance [4, 13]. A usual step to refine *de novo* assemblies is to reduce transcript redundancy. One popular tool is CD-HIT [14], which removes shorter redundant sequences based on sequence similarity. A more recently released clustering tool, RapClust [15], generates clusters based on the relationships exposed by multi-mapping sequencing fragments and is considerably faster than previous approaches. Several methods are usually compared to assess the overall quality, accuracy, contiguity and completeness of a *de novo* assembled transcriptome, including basic metrics for assemblies, contig-level metrics, and comparison to protein datasets from related species [9, 11, 16, 17].

Read mapping is a crucial step to estimate gene expression for further analysis, but is made difficult by sequencing errors and is dependent on characteristics of the reference (quality of gene annotation, relatedness to sequenced individuals, size, repetitive regions, ploidy, etc.) [18]. Mapping transcript reads to a reference genome has the additional challenge of crossing splice junctions, some of which may not be accurately annotated [3]. Multiple metrics can be used to determine performance of read aligners. Precision and recall are the usual metrics with simulated data, while evaluations without a priori known outcomes utilize mapping rate, base mismatch rate, detected transcripts or correlation of gene expression estimates to quantify performance [2, 19]. Most common short read aligners are based on hash tables, which are more accurate but slow, or a compressed FM-index, which is faster but less flexible with errors [2, 9]. When using a distant genome, sequence divergence between reads and the reference genome

may compromise results; nucleotide mismatches are more likely to decrease the number of mapped reads, while indels are usually better tolerated with gapped alignments [2]. One benefit from the utilization of a distant genome is a direct comparison of gene expression results from multiple related species [20]. On the other hand, utilization of *de novo* assemblies avoids the mapping issues to a distant genome and also captures divergent and novel genes useful for species-specific discovery of new functions. Selecting between a *de novo* transcriptome or a reference genome has been shown to produce comparable gene expression profiles at over 87% correlation in other systems but has not been examined in plants [5, 19].

Most prior papers examining the choice of informatics software for RNA-Seq data analysis worked with straightforward data sets, either performing a single type of analysis on the data or working with data from diploid organisms with well-developed reference genomes. However, much less research has been done into genomics of complex species and, especially in the case of plants, polyploids. Many polyploid crops now have available reference genomes, like strawberry [21], cotton [22], wheat [23], or sweet potato [24], while others continue to rely on genomic resources from diploid relatives, such as potato [25], kiwifruit [26], peanut [27], or blueberry [28]. Here, we have selected blueberry datasets as an example. A number of different species of blueberries are used in agricultural production and breeding, with autotetraploid *Vaccinium corymbosum* (highbush blueberry) as the most economically important [29]. In this study we use RNA-Seq data from an autotetraploid *V. corymbosum* and a diploid species, *V. arboreum*. The available reference genome is from a diploid *V. corymbosum* [28, 30].

Data description

The sequencing data used in this work is 270 million Illumina paired-end reads (2*101 bp long) for diploid *V. arboreum* (VA) and 582 million reads for tetraploid *V. corymbosum* (VC), originating from 8 plants each [20] and sequenced on duplicate lanes. Libraries were prepared from RNA collected from roots of plants of similar age after eight weeks of growth in hydroponic systems under either stressful (pH 6.5) or control (pH 4.5) conditions. All sequence data is publicly available at NCBI (see details

below). At the first step of data curation, our tested methods are error correction of RNA-Seq data with Rcorrector and trimming of low quality bases by one of two methods, Trimmomatic [31] or Skewer [32]. Error correction of raw reads modified an average of 0.7% bases per library, a proportion larger than the expected 0.1% sequencing error rate in Illumina reads and suggests a possible masking of variability in the data. Next, both original and corrected reads were trimmed using either Skewer or Trimmomatic at default settings. Gentle quality trimming with Skewer retained on average 99.6% reads at mean length 99.8 bp (Table S1). In contrast, quality trimming with Trimmomatic, which has significantly more aggressive default trimming parameters, retained 77.2% of reads at mean length 93.8 bp. Error correction did not have a significant effect on trimming results. From the combination of corrected/uncorrected reads and trimming software used, four read sets (reads processed by Rcorrector and Trimmomatic, Rcorrector and Skewer, Trimmomatic only, and Skewer only) for each species were used in downstream analyses.

Analysis

Construction of *de novo* assemblies

A series of *de novo* assemblies were carried out with the Trinity software. For each species, assemblies of a single control library, a single treatment library or a combination of both libraries were performed, using each of the four preprocessing techniques as input (Skewer corrected, Skewer uncorrected, Trimmomatic corrected, Trimmomatic uncorrected), to yield a total of 24 Trinity runs (Figure S1). For the assembly of two individual libraries, the results were combined post-assembly. The possible benefit of this approach is the reconstruction of specific transcripts from control and treated samples without mixture of alternative splice variants, at the expense of including a smaller data input size that may induce fragmentation of assemblies as well as a requirement to merge the separate assemblies afterward. This approach is contrasted to the second method, which combines multiple samples in a single assembly run; this approach aims at reconstructing longer and more complete transcripts despite mixing fragments from splice variants.

Table 1. *De novo* assembly basic metrics.

assembly	trimming	error correction	# input fragments	# output seqs	# output seqs <500bp	# output seqs >1kb	# output seqs >10kb	N50
VA_4s	skewer	-	36 148 028	329 614	255 716	29 022	27	550
VA_4s	skewer	+	36 148 810	330 075	256 072	29 054	26	552
VA_4s	trimmomatic	-	27 202 836	290 112	222 424	27 029	22	577
VA_4s	trimmomatic	+	27 204 308	291 843	223 430	27 275	26	579
VA_1sC	skewer	-	10 587 674	142 129	108 121	12 110	4	542
VA_1sC	skewer	+	10 587 893	143 209	107 881	12 828	4	565
VA_1sC	trimmomatic	-	8 236 566	127 214	96 277	10 726	3	544
VA_1sC	trimmomatic	+	8 236 881	128 668	96 432	11 516	4	564
VA_1sT	skewer	-	7 568 547	95 736	76 461	5 364	2	441
VA_1sT	skewer	+	7 568 703	96 587	76 517	5 712	4	453
VA_1sT	trimmomatic	-	5 271 314	82 949	66 043	4 718	1	444
VA_1sT	trimmomatic	+	5 271 955	84 136	66 482	5 018	2	454
VC_4s	skewer	-	80 878 048	632 185	492 743	49 578	34	515
VC_4s	skewer	+	80 879 542	636 227	494 632	50 564	32	521
VC_4s	trimmomatic	-	62 799 424	565 025	434 903	47 798	32	540
VC_4s	trimmomatic	+	62 801 807	569 258	436 967	48 755	32	547
VC_1sC	skewer	-	18 472 410	227 024	176 969	17 850	6	507
VC_1sC	skewer	+	18 472 731	230 322	177 699	19 286	14	529
VC_1sC	trimmomatic	-	14 504 065	203 961	158 201	16 373	16	517
VC_1sC	trimmomatic	+	14 504 527	207 763	159 491	17 698	10	536
VC_1sT	skewer	-	18 330 711	227 074	183 773	13 431	13	435
VC_1sT	skewer	+	18 331 169	230 852	185 160	14 487	10	449
VC_1sT	trimmomatic	-	14 570 654	202 713	163 406	12 184	6	440
VC_1sT	trimmomatic	+	14 571 002	206 743	165 075	13 078	11	454

Assemblies are formed by the combination of trimming software, error correction with Rcorrector, blueberry species (VA, *Vaccinium arboreum*; VC, *V. corymbosum*), and number of samples on the assembly (1s, one sample; C, control; T, treatment; 4s, four samples).

After each assembly run, the number of output sequences is highly correlated with the number of input fragments. The N50 statistic responded to the number of input samples used and, to less extent, trimming and error correction (Table 1). As the selection of trimming software directly impacts the number of fragments available to assemble, the

assemblies made after Trimmomatic have a lower number of transcripts and better N50 values. N50 with Trimmomatic was increased in comparison to Skewer by 5% on 4-sample assemblies and 0-1% on those from 1 sample. In agreement with previous reports showing improvement of assembly quality after using an error correction tool [7, 33], assemblies from corrected reads increased the number of transcripts by 1% and increased N50 by 2.5%. For all assemblies, the default minimum transcript length was 200 bp. 75-80% of assembled transcripts were shorter than 500 bp, representing putative assembly artifacts or transcripts encoding protein fragments or short proteins (10-200 amino acids [34]). This over-abundance of short assembled transcripts is reflected by the low N50 values, also around 500 bp. Increasing the number of input fragments also has a positive effect on the assembly of long transcripts, following similar trends with trimming and correction as the total number of transcripts.

Clustering of *de novo* assemblies

Assemblies may contain sequences from highly similar gene isoforms, transcript isoforms of a same gene and, in the case of polyploids, homeologous genes, that may be considered redundant and lead to reads mapping to multiple locations. In addition, considering that plants contain 37000 proteins on average [35], the number of transcripts from all of the *Vaccinium* assemblies (Table 1) largely surpasses this quantity. Tools aimed at the reduction of such redundancy are widely used to select non-redundant representative sequences [13, 36, 37]. We have compared the clustering capabilities from two tools with very different approaches. CD-HIT was used to select long representative transcripts and remove smaller redundant sequences at 95% similarity cutoff. RapClust groups transcripts based on the information of multi-mapped reads, and removes transcripts with low read support. CD-HIT returns a classification of transcripts into clusters and a set of representative transcripts with reduced redundancy, while RapClust returns clustering information suited to be used for downstream differential expression analysis but does not report a reduced transcript set. For the sake of comparing results, the longest transcript from each cluster generated by RapClust was selected to form corresponding reduced assemblies. Prior to clustering, single-sample assemblies were combined into a merged assembly, with expected introduction of high redundancy. Then, transcripts from the 16 assemblies (8 per species) (Figure S1) were subjected to classification into clusters with either of these tools.

In all cases RapClust produced fewer clusters than CD-HIT (Figure 2A, Table S2); on average, the number of clusters after CD-HIT and RapClust were 22% and 51% smaller than the initial number of transcripts, respectively. In addition, RapClust filtered out 9% and 24% of sequences on 2s and 4s assemblies, respectively, due to low read support (Table S2). The degree of clustering varies by type of assembly and species. There was less clustering in 4s than 2s assemblies, as shown by the 12% and 2.5% larger proportion of representative sequences in 4s retained after CD-HIT or RapClust, respectively. On average VA had slightly less clustering, with 3.2% more sequences retained as clusters than VC. This correlates with the putative higher redundancy in 2s assemblies, and by the presence of homeolog genes due to polyploidy in VC. The higher degree of clustering of RapClust yielded a larger mean number of transcripts per cluster and the largest clusters are one order of magnitude higher in number of member transcripts than those of CD-HIT (Table S2). However, both methods left a large proportion of transcripts unclustered; 74-87% and 58-77% of clusters for CD-HIT and RapClust, respectively, had a single member transcript (Table S2). Abundance of small sequences (<500 bp) remained high after clustering, on average 78%, constituting the majority of these single-member clusters. Despite very short transcripts (e.g. < 300 bp) are usually considered less informative, selection of a larger transcript length cutoff is not in the scope of the present work.

Detonate scores are used to compare a set of transcriptomes formed from the same set of reads, where values closer to zero indicate better assemblies. Detonate was used to evaluate the original assembled transcripts, the cluster representative sequences yielded by CD-HIT, and the longest transcript from each RapClust cluster. For initial assemblies, detonate scores are inversely correlated with the number of transcripts (Figure 2B), possibly reflecting the compactness component in detonate evaluation. All detonate scores were lower after clustering compared to initial assemblies, possibly reflecting a reduced support from RNA-Seq reads. Scores decreased by 87.2%, 102.5%, 1.8% and 15.1% in 2s CD-HIT, 2s RapClust, 4s CD-HIT and 4s RapClust, respectively. These rates were not influenced by species or read processing. Thus, despite reducing the initial score, clustering of assemblies has better evaluation when using CD-HIT

instead of RapClust, and when combining multiple samples in the assembly instead of separately assemble and merge.

Figure 2. Basic assembly metrics of initial Trinity assemblies, redundancy-reduced clusters and predicted cds. (A) number of transcripts, (B) Detonate scores and (C) N50 values. Lines are colored by assembly type: VA for *Vaccinium arboreum*, VC for *Vaccinium corymbosum*, 2s for 2 sample assembly strategy, 4s for 4 sample assembly strategy. Symbols indicate how reads were trimmed (trimm for Trimmomatic) and whether they were corrected (Uc for uncorrected, cor for corrected).

Annotation of *de novo* and clustered assemblies

In addition to assembly metrics, functional annotation of transcripts was done to assess putative biological information contained in transcriptomes. The first step for transcriptome annotation consisted of extracting coding sequences (cds) from transcripts with Transdecoder. This software finds all open reading frames (ORFs) and selects the most likely putative cds using homology search results from blast. 52-58% of transcripts contained a predicted cds for all assemblies. Compared to the length of original transcripts, the average length of cds decreased by 13% and 20% on 2s and 4s assemblies, respectively. The shortest cds sequences of 147 bp corresponded to the lower limit of 50 amino acids, after Transdecoder refining the start codon nucleotides. In contrast, the N50 value of cds was increased on average 24% compared to clusters, except in VC 4s assemblies that decreased by 5% (Figure 2C), possibly reflecting the reduction in total number of bases after discarding non-coding regions. N50 was consistently larger after RapClust than CD-HIT. These variations were dependent on type of assembly (species and samples), rather than read processing (correction and trimming).

To further explore the effect of clustering, we utilized the published reference genome from the diploid *Vaccinium corymbosum* [28]. We presented two scenarios, one with a distant diploid species and other with the same species but different ploidy level. To explore the portion of transcripts with sequence homology that each species shares with the reference genome, we mapped the clustered transcriptomes to it. Transcripts were

classified as uniquely mapping, mapping to multiple loci, translocated (parts of the transcripts were mapped to different locations on the genome) or not mapping. These results were combined with Transdecoder cds prediction and blast homology results. Overall, transcripts generated for the diploid VA mapped to the reference genome at a larger proportion than the tetraploid VC, and the 2-sample merged assemblies (2s) mapped at a higher rate than the 4-sample ones (4s) (Figure 3). Specifically, average mapping rate of transcripts was 66% and 57% in VA 2s and 4s, and 57 and 43% in VC 2s and 4s. Thus, the use of multiple samples leads to a higher proportion of transcripts not resembling the genome, representing species-specific transcripts and possibly artifacts. While VA has higher mapping rates than VC, discrimination between a true higher similarity or an effect due to the read input cannot be made. The proportion of multiple mapping and translocated transcripts had little variation across transcriptomes in both species, being 5-7% and 4% respectively. Multi-mapping rate reflects highly similar regions of the genome, and translocations could indicate either true genome rearrangements or assembly artifacts such as transcript fusions (chimeras). Clustering with CD-HIT or RapClust (using a single representative sequence for each cluster), despite affecting the total number of transcripts, maintained similar proportion of transcripts in each mapping category; on average, RapClust increased 2.2% unique and decreased by 0.5% multiple and translocated mapping transcripts compared to CD-HIT. Trimming also influenced mapping; assemblies from reads trimmed with Trimmomatic showed an average 2% higher unique mapping rate than their counterparts with Skewer, suggesting better accuracy with stricter trimming. No effect was observed from error correction.

Prediction of a coding sequence and the extent to which they may be coding for proteins was used as an indicator of biological information contained in transcripts. Transcripts within each category (unique, multiple, translocated and not mapping) had different likelihoods of having a predicted coding sequence and additionally of cds showing homology to known proteins. On average, 49.2%, 51.8%, 54.8% and 64.5% of the transcripts in the categories unique, multiple, translocated and not mapping, contained a predicted coding sequence (Figure 3). In addition, 54.0%, 42.4%, 55.2% and 20.1% of the cds on those categories, respectively, had a blast hit. Thus, a relatively large proportion of cds do not map to the genome, particularly in VC with 4 samples (72%).

These transcripts also show low similarity to known proteins, leaving unclear whether they belong to true novel transcripts or they are assembly artifacts. For transcripts that mapped to the genome, VA exhibited greater proportion of annotation than VC. Nonetheless, comparing absolute number of transcripts, VC has a larger set of mapping transcripts with cds but also an even larger number of transcripts not matching the reference than VA. Influence from the other analysis options on annotation distribution were less drastic. Clustering with RapClust had a positive effect on the proportion of cds and blast results of unique and translocated transcripts, especially in 2s assemblies, in the range of 0.5-5.5%. Changes due to read trimming or correction were lower than 2%.

Figure 3. Mapping of *de novo* assembly transcriptomes to *Vaccinium corymbosum* reference genome and annotation of transcripts. Transcripts mapped either uniquely to the genome (uniq), to multiple locations (mult), with translocations (transloc) or did not map (out). Annotation from prediction of coding sequences (cds) using homology results from blast is divided as “No Functional Annotation” (map), “CDS Only” (cds) and “CDS with Blast Hit” (blast). Transcriptomes derive from the combination of use (C) or not (U) of error correction, Trimmomatic (tr) or Skewer (sk) trimming tools, CD-HIT or RapClust clustering software, two (2s) or four (4s) samples, and blueberry species (VA and VC).

Quality assessment of assemblies and derivatives

To compare results throughout the sequential stages of transcriptome processing, the BUSCO tool was used to assess completeness of assemblies in relation to a select plant protein database that contains 1440 near-universal conserved orthologs. The results report for each BUSCO whether it is present in the assembly complete and single-copy, complete and duplicated, fragmented, or missing. Examining the impact on BUSCO results by read processing, assemblies from soft trimmed reads with Skewer presented higher completeness (Figure 4A). Interestingly, error correction improved the formation of complete BUSCOs on 2s assemblies, while it did not have a significant effect on 4s assemblies. However, the major options influencing completeness were blueberry species and number of samples used. Thus, assembly of complete genes was improved in VC compared to VA, and in assemblies of four rather than two samples (Figure 4A).

Overall, completeness of CD-HIT clusters was very similar to those of *de novo* assemblies, while RapClust clusters contained fewer total BUSCOs. Selection of cds further decreased completeness, either decreasing complete genes or also increasing fragmented genes, mostly in 4s assemblies. The distribution of complete vs fragmented BUSCOs follows a trend where a reduction in total BUSCOs is followed by an increase in fragmented BUSCOs (Figure 4A). Following this trend, the rate of fragmented BUSCOs was not significantly modified by read processing nor by clustering with CD-HIT, while RapClust increased it except in VA 2s, where fragmented BUSCOs were reduced.

While some gene families may have undergone expansion or contraction since the *Vaccinium* common ancestor, we expect the majority of transcripts to provide one-to-one orthologs for the VA gene set and two-to-one orthologs for the tetraploid VC gene set. Coincident with their ploidy, duplicated vs single-copy ratio in unclustered VA *de novo* assemblies was half that of VC (0.50 in 2s and 0.58 in 4s). Also, the duplication ratio in 2s vs 4s unclustered assemblies was 1.25 in VA and 1.45 in VC, supporting higher redundancy in 2s assemblies. These ratios are independent from the size of transcriptomes. Clustering was efficient to remove redundant genes, as shown by the reduction of duplicates. RapClust drastically removed most duplicated BUSCOs, leaving 20-30 duplicated BUSCOs for all assemblies, while CD-HIT performed a reduction proportional to the assembly length of 62% on 2s and 44% on 4s assemblies. While the clustering did remove many duplicated BUSCOs, most became single copy BUSCOs and were not lost from the assembly altogether. Only in the 4s assemblies, comparing the original assembly to RapClust cluster transcripts, there was a significant decrease in the number of complete BUSCOs (Figure 4B).

Figure 4. Evaluation of assembly and clustering methods. (A, B) Completeness assessment with BUSCO tool subdivided into complete versus fragmented BUSCOs (A) or single-copy versus duplicated complete BUSCOs (B). Dotted lines represent isolines of BUSCO numbers from a total search space of 1440 orthologs. Dot colors indicate assembly stage and areas assembly type. Stages of the assembly are divided into initial *de novo* assembly (asmb), clustered with either CD-HIT or RapClust, or

predicted coding regions (cds). Assembly type indicates the combination of blueberry species (V. *arboreum*, VA; *V. corymbosum*, VC) and the use of two independent assemblies merged (2s) or assembly of four samples (4s). Shapes represent read pre-processing options, with (cor) or without (Uc) error correction, and the use of Skewer or Trimmomatic (trimm) trimming tools. (C) Distribution of mean Jaccard scores on CD-HIT and RapClust clusters of transcriptome assemblies. Scores range between ~0 (low clustering of co-annotated transcripts) and 1 (perfect clustering of co-annotated transcripts). (D) Distribution of genome versus assembly base coverage on multiple *de novo* assemblies mapped to *Vaccinium corymbosum* reference genome after redundancy reduction with either CD-HIT (larger points) or RapClust (smaller points). Shapes indicate read processing, with (cor) or without (Uc) error correction, and trimmed with either Trimmomatic (trimm) or Skewer.

BUSCO results were not only used to assess completeness, but also to measure the success of the clustering methods using an adaptation of the Jaccard similarity method. Taking advantage of BUSCO consensus sequences, transcript co-annotation was calculated as the number of transcripts with the same BUSCO annotation within a cluster (set intersection) divided by the total number of transcripts with that BUSCO annotation or in the cluster (set union). The result is a value in the range 0 to 1, from low to perfect shared annotation of transcripts within a cluster. This method not only indicates the degree of co-annotation depicted by each clustering algorithm but also compares the putative biological relevance of clusters. On this respect, RapClust consistently outperforms CD-HIT on clustering of co-annotated BUSCO genes (Figure 4C). Clusters from the diploid VA were markedly better co-annotated from those of VC. Generally, RapClust performance was enhanced on larger transcriptomes, while CD-HIT performed better on smaller ones. In relation to read processing, Trimmomatic and uncorrected reads generally achieved higher scores.

To explore the percent of the blueberry genome captured by the *de novo* assemblies, base coverage was calculated for transcripts that mapped uniquely to the diploid reference genome (Figure 4D). Assembly base coverage is the proportion of bases of each transcript assembly that were mapped to the reference genome, and genome base coverage is the proportion of the reference genome covered by the transcripts. In

general, both metrics showed inverse correlation. Thus, genome coverage was enhanced with the use of Skewer, four samples and CD-HIT, while decreasing assembly coverage. Thus, genome coverage is concordantly improved by those options that also increase transcriptome size, where a larger number of transcripts is able to better represent genomic sequences. This is true for both blueberry species, with the distinction that VC exhibits both better genome and assembly coverage than VA, consistent with phylogenetic proximity to the reference genome species. On the other hand, trimming with Trimmomatic, two-sample assemblies and clustering with RapClust had better assembly coverage, but lower genome coverage. This suggests that transcripts generated from more restrictive options are more likely to be real genes that can be found in the genome, but the more restrictive options do exclude some genes. Error correction did not follow this trend, and generally decreased assembly coverage while not affecting genome coverage.

Read mapping to reference genome

As an alternative to *de novo* assembly, RNA-Seq analysis for these two species could utilize a mapping approach with the publicly available genome of diploid VC. With this approach, an entirely different set of software options become available. In this case, mapping to a genomic reference that is evolutionarily diverged from the sequenced species may make accurate read mapping more difficult. To account for sequence divergence, we compared results from five representative mapping software programs, run with either default settings or increasing mismatch tolerance (Figure 5A). Overall, aligners behave similarly on both blueberry species. The programs that yield the most mapped reads are Stampy and GSNAP, both of which were designed to tolerate more sequence divergence during mapping, although only Stampy surpassed 5% mismatch rate (Figure 5B). Bowtie2 and HISAT2 yielded the lowest mapping rates. The addition of relaxed conditions, despite modifying the percent of mismatches tolerated on alignments, did not have a significant effect on mapping results of GSNAP, Stampy and STAR; it lowered the mapping rate for Bowtie2 and increased for HISAT2, especially in VA. The effect of trimming was correlated with the number of available reads to be mapped; thus, Skewer improved mapping rates by 5-11% compared to Trimmomatic (Table S3). Finally, corrected reads, though not significant, promoted an increase in

mapping rate for all options, with 0.7 and 0.5% average increase in VA and VC, and up to 2.5% in HISAT2 in VA.

It is desirable to utilize the maximum number of reads as possible in differential gene expression analysis, as increased depth of read counts leads to more sensitivity in statistical analysis. For example, more depth would increasingly allow detection of differences in lowly expressed genes or genes with small log fold changes in expression between treatments. To use this as a quality metric, we examined the successful conversion of raw reads to countable reads for each gene model using the software HTSeq. Starting from all mapping results, a read may not be converted to a countable read due to low quality mapping, multiple alignments or mapping to a genomic region without an annotation. The influence of each factor varies by mapping tool (Figure S2). The main cause of failed read conversion into counts was low quality of read alignment, found in Bowtie2, HISAT2, Stampy and GSNAP, by decreasing magnitude. The second major factor that prevented counting was mapping within an intergenic region, which accounted for 5-13% of mapped reads (Figures S2 and S3). Mapping to exonic features showed even larger variability, ranging from 57% displayed by Stampy, to 80% by HISAT2, varying by mapping tool (Figure S3). In relation with mapping rate, these values indicate that both programs have similar mapping rates to exons but Stampy is mapping more reads to non-exonic regions that may present higher sequence divergence. After collecting useful read counts, count rates to gene models were smaller than mapping rates by 14.2%, 10.9%, 7.5%, 15.7% and 3.3% for Bowtie2, GSNAP, HISAT2, Stampy and STAR, representing a loss up to 45% of mapped reads for Bowtie2 and below 15% for STAR (Figure 5A, right panels). Globally, modification of mismatch tolerance increased this loss in Bowtie2 and Stampy, and reduced it in HISAT2. Read loss using Skewer compared to Trimmomatic was larger on GSNAP and Stampy, and smaller on HISAT2 and Bowtie2.

Interestingly, the rate of mapped reads not turned into counts in STAR was constant under the pre-processing and software options tested. After counting, count rates (Figure 5A, lower values) displayed similar response to read processing as mapping rates discussed above, with GSNAP and Stampy showing equally high count rates.

Figure 5. Read mapping to *V. corymbosum* reference genome. (A, left panels) Proportion of total reads mapping to reference (grey boxes or higher values), converted to counts (white boxes or lower values) and (A, right panels) percentage of the difference, and (B) mismatch rate depicted by each software option. Five mapping software programs were compared at default and modified settings to increase mismatch tolerance. Reads used (cor) or not (Uc) error correction, and Trimmomatic (trimm) or Skewer trimming software. Results are distribution of 8 samples.

An important issue in science is reproducibility of results, that in the case of mapping results can be reflected as similarity of gene count profiles, which ultimately determine genes that are differentially expressed. Correlation of counts was calculated across all blueberry samples comparing the 20 combinations of read processing and mapping software with default options (Figure 6). Concomitant with their similarity on mapping results to the reference genome, VA and VC shared major correlation patterns between software programs, where two major groups are formed. This grouping is consistent with the alogrithmic similarities of the software, i.e. one group is composed by Bowtie2 and HISAT2, which utilize an FM-index, and the second group includes GSNAP, Stampy and STAR, which use a combination of suffix array / hash table. Correlation was usually influenced by the trimming option, so that Skewer significantly improved correlation on GSNAP and STAR, Trimmomatic on Bowtie2 and Stampy, and HISAT2 was ligthly affected by trimming. Interestingly, only Bowtie2 and HISAT2 responded to read correction, suggesting higher sensitivity to errors by the FM-index.

Figure 6. Correlation of gene count profiles after mapping to *Vaccinium corymbosum* genome. Values are mean of 8 samples in either *V. arboreum* (VA, upper triangle) or V. *corymbousm* (VC, lower triangle). Each row/column corresponds to a unique combination of mapping software, trimming software and error correction.

Read mapping to *de novo* assemblies

The previous section focused on the effects of read correction, trimming and alignment software on read mapping to a reference genome. Here, a similar analysis is performed though using *de novo* assemblies clustered with CD-HIT. To simplify the analysis,

reads that underwent certain correction and trimming processing (e.g. samples with corrected reads trimmed with Skewer), were only mapped to the assemblies produced by reads with the same pre-processing. This method of *de novo* assembly then alignment is common for RNA-Seq analysis when no reference genome is available, and has advantages, including that mapping to transcript assemblies is usually contiguous, instead of spliced, and that assemblies are species specific, unlike a distant reference genome. All the aligners previously used for the genome alignment may also be used with transcriptomes. In addition, we incorporated the Salmon tool for transcript quantification, which is built solely for alignment of reads to a transcriptome.

Using *de novo* assemblies as the reference, mapping performance of the five aligners showed lower variability by condition (trimming and type of assembly) compared to mapping to the genome, with Stampy and GSNAP again as best performers (Figure 7). The mapping profile was similar for both species, with higher mapping rates for VC than VA by 1.4% using Skewer and 2.5% using Trimmomatic, except for Salmon. Also, 4s assemblies had consistently better mapping rates than 2s, with improvements for Skewer/Trimmomatic of 3.7/3.0% in VA and 3.8/3.4% in VC. Examining only the effect of trimming, yield is likewise correlated with the number of reads available for mapping, so that Skewer had on average 12.5% more reads mapped than Trimmomatic. Finally, error correction of reads did not have a significant effect on read mapping. Examining conversion of raw reads to countable reads, 30-45% and 22-30% of mapped reads in 2s and 4s assemblies were not able to be turned into counts, with higher values on 2s assemblies than 4s ones (Figure 7, right panels). For Bowtie2 and Stampy, the major cause of read loss was low quality alignments, while for GSNAP, HISAT2 and STAR most of the dropped reads were multi-mapped (Figure S4). Read counts further reduced variability across programs, and intensified the difference between mapping to 4s compared to 2s assemblies, increasing by 9.1/6.1% in VA and 9.8/7.9% in VC for Skewer/Trimmomatic, respectively. The difference between using Skewer or Trimmomatic was reduced to an average of 9%. The different results yielded by Salmon reflects its different algorithm, which performs pseudo-mapping to estimate abundance, but does not report mapping results in a format suitable to do quality assessment of alignments. The consequence is that Salmon has an artificially higher estimated count

rate than reads mapped, and since no reads are filtered out for quality score, Salmon has higher count rates than other approaches.

Figure 7. Read mapping to CD-HIT clustered *de novo* assemblies. Proportion of total mapped reads (left panels, grey boxes), converted to counts (left panels, white boxes) and percentage of the difference (right panels). Six mapping software programs were compared at default settings on assemblies made from four samples, produced either by two sets of 2 samples independently assembled (2s) and later merged or from the four samples assembled together (4s). Reads used (cor) or not (Uc) error correction, and Trimmomatic (trimm) or Skewer trimming software.

In the case of mapping to a *de novo* assembly, to calculate a correlation of mapping results is not directly due to each assembly having their own set of transcripts. Hence, rather than program-to-program correlation, which is showed on the previous section, reference-to-assembly count profiles were compared (Figure 8). To do so, the reference gene model gene space was used for such comparison. New count profiles for assembly mapping results were obtained from adding counts of all transcripts mapped to each single reference gene model. Then, they were compared to results with the reference genome by same read pre-processing and mapping software. Utilization of the reference genome from diploid VC, though useful for a shared gene set to compare, has the inconvenience of not representing species-specific transcripts (blue bars in Figure 3). VA is a sister species but is also a diploid, so one-to-one homology may be expected. However, tetraploid VC assemblies not only contain a larger proportion of transcripts that do not match the genome, but also splice isoforms and lowly-diverged homeolog sequences are expected to map to same gene models. Likewise, balancing this effect, reads originated from transcripts sharing sequence similarity are expected to map to the same gene model on the reference genome.

The highest assembly-to-genome correlation values are obtained on the diploid VA, which reach 75% on all programs (Figure 8). However, the best performing program differs by species: GSNAP and Stampy for VA, and Bowtie2 and HISAT2 for VC. For both species, results with the larger 4s assemblies are better correlated to the genome than the 2s assemblies. Overall, the preference for trimming software, if any, is opposite

by species; Skewer and Trimmomatic improves 2s and 4s assemblies on VA, respectively, and Skewer improves 4s assemblies in VC. These differences caused by read processing are more prominent on 4s assemblies, while on 2s assemblies they induce significant changes on VA with Bowtie2, HISAT2 and STAR. This suggests that stricter trimming in the distant VA may help mapping accuracy on the diploid VC genome, especially with Bowtie2 and HISAT2 4s, while gentle trimming in the tetraploid VC may help by either better assembly of transcripts or read mapping. Salmon results correlate well with the different aligners in VA, especially GSNAP and Stampy (Figure 8, bar colors), while the tetraploid VC has overall poorly-comparable results. This suggests that Salmon transcript quantification may be better suited for less complex genomes.

Figure 8. Correlation of gene count profiles obtained with *de novo* assemblies and the reference genome. Counts of transcripts aligned to a same reference gene model were added and re-annotated as that gene model. Correlation was calculated on the common set of gene models with non-zero counts on both reference and assemblies, by mapping software and read pre-processing (error correction and trimming). Uc stands for uncorrected, cor for corrected, trimm for Trimmomatic. Color indicates mean correlation of reference counts with Salmon, a transcript-specific quantification tool. Values are mean ± sd of 8 samples.

Discussion

RNA-Seq is an affordable and versatile tool to analyze transcriptomes of any species. Depending on the available resources, it can be guided by a reference genome or by building custom assemblies that will reflect the transcripts present in the samples. However, many confounders make the analysis less straight-forward than simply trimming adapters, assembling reads as needed and mapping to a reference. Some of these confounders are common for any RNA-Seq data analysis, such as sequencing errors, repetitive sequences, natural heterozygosity and variants, while the analysis of a species other than the reference has additional sequence variation and, in the case of a

polyploid, gene redundancy. Thus, we explored the repercussions of various informatic choices on the final gene expression profiles.

Illumina short read sequencing, though very accurate, is not exempt of sequencing errors. One strategy to deal with low quality nucleotides aims to correct reads, usually by replacing poorly represented *k*-mers with similar ones of higher frequency patterns [33]. Effectivity of error correction on RNA-Seq data is lower than on genomic data due to differences in expression level and splicing and is less dependent on the organism of study [7]. Despite sequencing errors of Illumina technology occurring at a reported average rate of only 0.1% bases [6], Rcorrector modified 0.7% bases in both species. While error correction tools can reduce sequencing errors, they can also introduce new errors at a variable rate, especially for complex datasets [33]. For a complex gene family or when examining a polyploid, this could be a significant problem with some reads converted to the sequence of a close homolog, leading to incorrect mapping and/or misassembly. However, in this study read correction did not reflect significant variation in overall mapping success. It induced a small amount of variation only on those aligners that use an FM-index, Bowtie2 and HISAT2, and thus require perfect matching for seeding an alignment. Read correction was more important for assemblies, which exhibited larger changes depending on correction state, such as improvement of completeness when using corrected reads in most cases. Previous research also demonstrated that error correction impacts genome assembly [33].

Trimming is required to, at the least, remove sequencing adapters, and often also addresses short reads and low quality bases. The broadly-used tool Trimmomatic implements strict trimming based on sequencing base quality, where trimming removes low quality bases that could lead to complex or incorrect de Bruijn graphs, but also reduces read length, which may have a negative impact on coverage bias [33]. Skewer takes a much less stringent trimming approach. The extent to which trimming of low quality bases is beneficial for downstream analyses was explored for DNA-Seq [38], suggesting a positive effect on genome assembly despite increased fragmentation, and a tradeoff between accuracy and recall of assemblies. In our experiments, similar effects derived from trimming were shown on both the diploid or tetraploid species. We found that Trimmomatic (i.e. strict quality trimming) reduced fragmentation of assemblies and

enhanced biological consistency of clustering, while Skewer (soft trimming) led to more complete assemblies at the expense of a larger amount of non-coding transcripts. In mapping experiments, higher quality reads are mapped at a larger relative proportion, however, this is at the expense of losing many reads at the trimming stage, many of which may have been successfully mapped downstream. Nonetheless, both options can lead to comparable expression profiles, mostly if mapping tools can deal with bases of lower quality [38].

There are cases where transcriptome assemblies are required, such as absence of a suitable reference genome, or discovery of novel isoforms. For transcriptome assembly with samples derived from various conditions, two approaches are common; one in which the samples are pooled into a single run [36, 37] and one in which samples are assembled independently [39-41]. The major interest is to obtain transcripts that are specific to each sample, and combination of reads is a potential source for mis-assembly or formation of chimeras. In this respect, we found that transcripts from separate samples had significantly higher assembly base coverage (transcript bases mapped to the reference genome), although the combined samples had better genome base coverage (reference genome bases covered by transcripts). However, merging individual assemblies generates high redundancy. Redundant merged assemblies show improved read mappability, but less continuity than assemblies from pooled samples, and their quality decreases after clustering [39]. We found a strong reverse correlation between fragmentation of genes and assembled reads, supporting that sequencing depth is beneficial to the recovery of full-length transcripts [13, 16, 42]. General conclusions apply to both the diploid and the tetraploid species, although the polyploid had proportional increased duplication rate and exhibited a larger species-specific proportion of transcripts. On the other hand, proper clustering in polyploids is difficult, not unexpectedly, as it must handle isoforms of genes as well as homeologs. This is reflected by the outcomes of the clustering methods utilized, where aggressive reduction of redundancy also leads to loss of completeness, though to a lesser extent than sequencing depth.

Scientists examining organisms without a specific reference face the decision of whether to use the reference genome of a close organism or to build a custom *de novo*

assembly. Mapping to a distant reference has disadvantages, including sequence divergence at the nucleotide level, and also larger structural divergence, where genes may be missing or duplicated between the species. From our species studied, it would be expected for the distant diploid VA to have undergone greater sequence divergence than the tetraploid relative of the reference diploid VC, in which divergence would be driven by diversifying subgenomes. Mapping results to the reference genome reflect this issue, where mapping tools that have greater sensitivity to align divergent sequences, such as Stampy, GSNAP and STAR, improve mapping results of VA compared to VC, while HISAT2 and Bowtie2, which require an exact match to seed, perform better in VC than VA. Regardless of the species, we found GSNAP and Stampy to yield the highest performances on the reference genome, probably due to their ability to align divergent sequences even at default settings. On the second mapping strategy, utilizing specific assemblies allowed much higher mapping rates compared to the reference, concordant with the high proportion of transcripts not represented on the genome that are now available to be mapped. Both species displayed comparable results when mapping to an assembly, slightly better on the tetraploid VC than on the diploid VA except with Salmon, probably due to the better completeness of the VC transcriptomes. In addition of higher mapping rates, specific biological information may be present on transcripts not represented in the genome, from which 64.5% had a predicted cds, gaining insight in the processes under study. Nonetheless, besides the divergence with the reference genome, using assemblies can give similar results at 75% correlation; awareness of mismatches also played here a role, improving correlations of VA with GSNAP and Stampy, and of VC with HISAT2.

In conclusion, using a reference genome with either a distant diploid species or a polyploid relative can give reliable results, simplifying the RNA-Seq analysis by skipping *de novo* assembly and associated steps. In the present work, we expanded many possibilities from read processing to gene counting, providing a complete overview on how each of the tested options impacts gene expression profiles. On both species studied, the pipeline that yielded high outcome with comparable results using either a reference genome or a transcriptome assembly used trimming with Skewer, a combination of multiple samples for improved assembly quality, and Stampy or GSNAP for short-read mapping. This pipeline was oriented to maximize the recovery of

information from RNA-Seq reads, working with the specific case where samples and reference genome are not from the same organism. While we suggest that this strategy can be extrapolated to other systems, our study also highlights the many downstream impacts software analysis decisions can have on results. For scientists faced with complex RNASeq analysis projects, testing of different software packages to examine and optimize results can be beneficial.

Methods

The following methods include a brief summary of the tools that were used in this work. For detailed descriptions of the algorithms, original publications or websites are referred.

Sequencing of RNA-Seq reads of blueberry roots

Preparation of RNA-Seq libraries from root tissue of diploid *Vaccinium arboreum* cultivar FL148 and tetraploid *V. corymbosum* 'Emerald' blueberry species are previously described [20] and available in NCBI as bioproject PRJNA353989. Briefly, eight plants per species were acclimated to growth in hydroponic systems at either pH 4.5 or pH 6.5 for 8 weeks, after which roots were collected and flash frozen. RNA was extracted and prepared for sequencing of 100 base-pair (bp) paired-end reads on a HiSeq 2000 system (Illumina, CA, USA).

Error correction and trimming of RNA-Seq reads

Rcorrector (*RNA-Seq error CORRECTOR*) [7] is a *k*mer-based error correction method that uses a De Bruijn graph to represent trusted *k*-mers, a method similar to that used on *de novo* assembly. Rcorrector v1.0.2 was applied to raw reads with default parameters. Then, sets of corrected and uncorrected reads were trimmed for removal of Illumina adapter sequences using either Trimmomatic v0.35 [31], specifying parameters 'SLIDINGWINDOW:4:15' and minimum read length of 30 bp, or Skewer v0.2.2 [32], with same minimum length cutoff. Trimmomatic searches adapters by finding an approximate match and aligning using a *seed and extend* approach [43], both for regular and 'adapter read-through' scenarios. Illumina quality scores of bases are used to determine cut points, discarding the 3' end of the read. Skewer uses a novel *bit-masked*

k-difference matching dynamic programming algorithm, which uses a variation of the *Smith-Waterman* [44] algorithm to search substrings and solve the *k-difference problem* and an extended *bit-vector algorithm* [45] to handle base-call quality values. Skewer can remove low quality bases on both 5' and 3' read ends, and is considerably faster than Trimmomatic. FastQC v0.11.4 [46] was used for quality assessment of reads. From each original read file (VA control, VA treatment, VC control, VC treatment), the combination of error correction and trimming generated four new sets of trimmed reads to be utilized in downstream processes: reads processed by Rcorrector and Trimmomatic, reads processed by Rcorrector and Skewer, reads processed by Trimmomatic only and reads processed by Skewer only.

de novo transcriptome assembly and redundancy reduction

Each of the four processed read sets was used for transcriptome *de novo* assembly, independently for each blueberry species, using Trinity 2.2.0 [12]. Environmental stress is expected to alter the transcripts present in the cells as well as transcript splicing patterns. To include this source of variability, two commonly used approaches were considered: (i) assemble control and treated samples independently and concatenate results after assembly, and (ii) combine two control and two treated samples in the same assembly run. Altogether, 12 Trinity assemblies for each species were generated (Figure S1). The next step consisted of removing redundant transcripts from assemblies using either CD-HIT v4.6.6 [14] at 95% identity or RapClust [47]. CD-HIT sorts all transcripts by length and attempts to consecutively cluster smaller sequences to longer representative ones, getting classified as redundant or representative based on sequence similarity; the result included a reduced transcript set consisted of one sequence per cluster. On the other hand, RapClust was developed to group assemblies using information from multi-mapper paired-ended reads, thus requiring input from Salmon [48] aligner. From the clustering information after RapClust, reduced transcriptomes were obtained after selection of the longest transcript per cluster. This step generated 16 clustered assemblies for each species (Figure S1).

Quality assessment and functional annotation of assemblies

Trinity assemblies and clustered assemblies were assessed for quality with DETONATE 1.11 [49] to calculate a score weighed with the reads used to generate each assembly, Transrate 1.0.3 [11] to get basic metrics, and BUSCO v2.0 [17] for completeness assessment. To compare the *de novo* assemblies to the genome, reduced assemblies were mapped to the diploid blueberry reference genome [30] with gmap version 2017-05-08 [50]. Base coverage was calculated on uniquely mapping transcripts using coverageBed from the BEDTools suite version 2.26 [51].

Biological consistency of clustering results was evaluated with a custom Jaccard similarity score based on the method described in [52] using the BUSCO annotation results. Each cluster received an individual score calculated as the number of transcripts with the same BUSCO annotation within the cluster divided by the total number of transcripts with that BUSCO annotation plus the number of transcripts in the cluster that did not share that annotation. The statistic is based on amount of the intersection divided by amount of union where the two sets are (i) all the transcripts sharing a BUSCO annotation and (ii) all the transcripts in a cluster. If multiple annotations were present in a cluster, the maximum score was selected for that cluster. The result is a value between 0, indicating low co-annotation of transcripts, and 1, indicating perfect clustering of co-annotated transcripts. Clusters with a single transcript were omitted.

Putative open reading frames (ORFs) were predicted for each clustered assembly with TransDecoder v3.0.0 [53], software that incorporates results from blast [54] and Pfam [55] homology searches to select best ORF candidates. First, candidate cds encoding at least 50 amino-acid-long peptides were extracted from transcripts. Then, these were searched with blast against the plant TrEMBL protein database (evalue < 10e-5) and with HMMER 3.1b2 [56] against Pfam. Finally, a single putative ORF was selected for each transcript when possible.

Read mapping

The four sets of processed RNA-Seq reads from VA and VC were mapped to either the draft reference genome for diploid VC or *de novo* assemblies clustered with CD-HIT,

using STAR 2.5.0, Stampy v1.0.28, GSNAP 2016-11-07, Bowtie2 2.2.8 and HISAT2 2.0.4. Software options were modified or not when mapping to the reference genome to increase mismatch tolerance. Salmon v0.7.2 [48], that uses quasi-mapping with a two-phase inference procedure, was specifically used on transcriptomes. Mapping metrics were collected using picard tools v2.1.0 [57] and RNA-SeQC v1.1.8 [58]. Finally, counts were obtained using HTSeq-count Version 0.6.1p1 [59].

Short read aligners can be classified by algorithmic approach as not splice-aware (Bowtie2, Stampy) or splice-aware (HISAT2, STAR, GSNAP), or by their use of an uncompressed index, such as hash table, or compressed indexes, like suffix arrays, Burrows-Wheeler transform (BWT) methods and Full-text index in Minute space (FM-index). Bowtie2 [60] uses an algorithm based on the BWT and the FM-index, which extracts seed substrings from reads, finds exact alignments with the FM index and extends with gapped dynamic algorithms like *Needleman-Wunsch* (global alignment) or *Smith-Waterman* (local alignment). Stampy [61] uses a hash table with locations of 15-mers in the genome used to search every overlapping 15-mer in the reads. Those that pass neighborhood similarity filtering are extended with *Needleman-Wunsch*. GSNAP (*Genomic Short-read Nucleotide Alignment Program*) [50] combines a set of algorithms to improve accuracy of alignment, using either hash tables or enhanced suffix arrays (ESA). Sequentially after failure of previous methods, GSNAP searches for a single continuous match, applies segment combination procedures, or employs its complete set analysis to allow for larger mismatch proportion. STAR (*Spliced Transcripts Alignment to a Reference*) software [62] is based on an algorithm that uses “sequential maximum mappable seed search in uncompressed suffix arrays followed by seed clustering and stitching procedure”. After stitching of seeds, the unmapped portions of the reads can be extended with *Needleman-Wunsch* algorithm. HISAT2 (*Hierarchical Indexing for Spliced Alignment of Transcripts*) [63] is based on the BWT and the FM-index, with operation methods adapted from Bowtie2. In addition to the global FM index, the genome is divided into a large set of small FM indexes. Read strings are first mapped to the global FM index to find candidate locations and the remaining bases are aligned with a local index, combining extension by direct comparison of sequences and further local index search of unaligned fragments.

826 # Availability of supporting data

827 The RNA-Seq data was deposited in the SRA database from the publicly available 828 repository NCBI, https://www.ncbi.nlm.nih.gov/sra/?term=SRA496374.

829 # Declarations

830 ## List of abbreviations

831 BUSCO benchmarking universal single-copy orthologs

832 cds coding DNA sequence

833 VA *Vaccinium arboreum*

834 VC *Vaccinium corymbosum*

835 ## Competing interests

836 The authors declare no competing financial interests.

837 ## Funding

838 This research was supported by the National Institute of Food and Agriculture, U.S. 839 Department of Agriculture, under award number 2009–02533 and the Thad Cochran 840 Southern Horticultural Laboratory, U. S. Department of Agriculture Agricultural 841 Research Service, under NACA agreement number 58–6062–5-004.

842 ## Author Contributions

843 GN, JO and TR prepared the biological material and collected sequencing data. MS and 844 MPM conceived and designed the analysis workflow. MPM performed computational 845 analysis of the data. MPM and MS analyzed the results and prepared figures. MPM and 846 MS contributed to the writing of the manuscript. All authors read and approved the final 847 manuscript.

848 ## Acknowledgements

849 We thank R.L. Darnell and V. Jones for their guidance and support in development of 850 hydroponic experiments, H.P. Rodriguez-Armenta and W.R. Collante for their skillful

support in RNA extraction and preparing libraries for sequencing, and Sun Xiaocun for support in statistics.

References

1. Conesa A, Madrigal P, Tarazona S, Gomez-Cabrero D, Cervera A, McPherson A, et al. A survey of best practices for RNA-seq data analysis. Genome Biol. 2016;17:13. doi:10.1186/s13059-016-0881-8.
2. Lindner R and Friedel CC. A comprehensive evaluation of alignment algorithms in the context of RNA-seq. PLoS One. 2012;7 12:e52403. doi:10.1371/journal.pone.0052403.
3. Engstrom PG, Steijger T, Sipos B, Grant GR, Kahles A, Ratsch G, et al. Systematic evaluation of spliced alignment programs for RNA-seq data. Nat Methods. 2013;10 12:1185-91. doi:10.1038/nmeth.2722.
4. Wang S and Gribskov M. Comprehensive evaluation of de novo transcriptome assembly programs and their effects on differential gene expression analysis. Bioinformatics. 2017;33 3:327-33. doi:10.1093/bioinformatics/btw625.
5. Nookaew I, Papini M, Pornputtapong N, Scalcinati G, Fagerberg L, Uhlen M, et al. A comprehensive comparison of RNA-Seq-based transcriptome analysis from reads to differential gene expression and cross-comparison with microarrays: a case study in Saccharomyces cerevisiae. Nucleic Acids Res. 2012;40 20:10084-97. doi:10.1093/nar/gks804.
6. Goodwin S, McPherson JD and McCombie WR. Coming of age: ten years of next-generation sequencing technologies. Nat Rev Genet. 2016;17 6:333-51. doi:10.1038/nrg.2016.49.
7. Song L and Florea L. Rcorrector: efficient and accurate error correction for Illumina RNA-seq reads. Gigascience. 2015;4:48. doi:10.1186/s13742-015-0089-y.
8. Macmanes MD. On the optimal trimming of high-throughput mRNA sequence data. Front Genet. 2014;5:13. doi:10.3389/fgene.2014.00013.
9. da Fonseca RR, Albrechtsen A, Themudo GE, Ramos-Madrigal J, Sibbesen JA, Maretty L, et al. Next-generation biology: Sequencing and data analysis approaches for non-model organisms. Mar Genomics. 2016;30:3-13. doi:10.1016/j.margen.2016.04.012.
10. Staiger D and Brown JWS. Alternative Splicing at the Intersection of Biological Timing, Development, and Stress Responses. Plant Cell. 2013;25 10:3640-56. doi:10.1105/tpc.113.113803.
11. Smith-Unna R, Boursnell C, Patro R, Hibberd JM and Kelly S. TransRate: reference-free quality assessment of de novo transcriptome assemblies. Genome Res. 2016;26 8:1134-44. doi:10.1101/gr.196469.115.
12. Haas BJ, Papanicolaou A, Yassour M, Grabherr M, Blood PD, Bowden J, et al. De novo transcript sequence reconstruction from RNA-seq using the Trinity platform for reference generation and analysis. Nat Protoc. 2013;8 8:1494-512. doi:10.1038/nprot.2013.084.
13. Zhao QY, Wang Y, Kong YM, Luo D, Li X and Hao P. Optimizing de novo transcriptome assembly from short-read RNA-Seq data: a comparative study. BMC Bioinformatics. 2011;12 Suppl 14:S2. doi:10.1186/1471-2105-12-S14-S2.

14. Fu L, Niu B, Zhu Z, Wu S and Li W. CD-HIT: accelerated for clustering the next-generation sequencing data. Bioinformatics. 2012;28 23:3150-2. doi:10.1093/bioinformatics/bts565.

15. Srivastava A, Sarkar H, Malik L and Patro R. Accurate, Fast and Lightweight Clustering of de novo Transcriptomes using Fragment Equivalence Classes. arXiv preprint arXiv:160403250. 2016.

16. O'Neil ST and Emrich SJ. Assessing De Novo transcriptome assembly metrics for consistency and utility. Bmc Genomics. 2013;14 doi:Artn 46510.1186/1471-2164-14-465.

17. Simao FA, Waterhouse RM, Ioannidis P, Kriventseva EV and Zdobnov EM. BUSCO: assessing genome assembly and annotation completeness with single-copy orthologs. Bioinformatics. 2015;31 19:3210-2. doi:10.1093/bioinformatics/btv351.

18. Thankaswamy-Kosalai S, Sen P and Nookaew I. Evaluation and assessment of read-mapping by multiple next-generation sequencing aligners based on genome-wide characteristics. Genomics. 2017;109 3-4:186-91. doi:10.1016/j.ygeno.2017.03.001.

19. Benjamin AM, Nichols M, Burke TW, Ginsburg GS and Lucas JE. Comparing reference-based RNA-Seq mapping methods for non-human primate data. BMC Genomics. 2014;15:570. doi:10.1186/1471-2164-15-570.

20. Paya-Milans M, Nunez GH, Olmstead JW, Rinehart TA and Staton M. Regulation of gene expression in roots of the pH-sensitive Vaccinium corymbosum and the pH-tolerant Vaccinium arboreum in response to near neutral pH stress using RNA-Seq. Bmc Genomics. 2017;18 doi:ARTN 58010.1186/s12864-017-3967-0.

21. Hirakawa H, Shirasawa K, Kosugi S, Tashiro K, Nakayama S, Yamada M, et al. Dissection of the octoploid strawberry genome by deep sequencing of the genomes of Fragaria species. DNA research : an international journal for rapid publication of reports on genes and genomes. 2014;21 2:169-81. doi:10.1093/dnares/dst049.

22. Li F, Fan G, Lu C, Xiao G, Zou C, Kohel RJ, et al. Genome sequence of cultivated Upland cotton (Gossypium hirsutum TM-1) provides insights into genome evolution. Nature biotechnology. 2015;33 5:524-30. doi:10.1038/nbt.3208.

23. International Wheat Genome Sequencing C. A chromosome-based draft sequence of the hexaploid bread wheat (Triticum aestivum) genome. Science. 2014;345 6194:1251788. doi:10.1126/science.1251788.

24. Yang J, Moeinzadeh MH, Kuhl H, Helmuth J, Xiao P, Haas S, et al. Haplotype-resolved sweet potato genome traces back its hexaploidization history. Nature plants. 2017;3 9:696-703. doi:10.1038/s41477-017-0002-z.

25. Genome sequence and analysis of the tuber crop potato. Nature. 2011;475 7355:189-95. doi:http://www.nature.com/nature/journal/v475/n7355/abs/nature10158-f1.2.html - supplementary-information.

26. Huang S, Ding J, Deng D, Tang W, Sun H, Liu D, et al. Draft genome of the kiwifruit Actinidia chinensis. Nature communications. 2013;4:2640. doi:10.1038/ncomms3640.

27. Bertioli DJ, Cannon SB, Froenicke L, Huang G, Farmer AD, Cannon EK, et al. The genome sequences of Arachis duranensis and Arachis ipaensis, the diploid ancestors of cultivated peanut. Nature genetics. 2016;48 4:438-46. doi:10.1038/ng.3517.
28. Gupta V, Estrada AD, Blakley I, Reid R, Patel K, Meyer MD, et al. RNA-Seq analysis and annotation of a draft blueberry genome assembly identifies candidate genes involved in fruit ripening, biosynthesis of bioactive compounds, and stage-specific alternative splicing. Gigascience. 2015;4:5. doi:10.1186/s13742-015-0046-9.
29. Hancock JF, Lyrene P, Finn CE, Vorsa N and Lobos GA. Blueberries and cranberries. Temperate fruit crop breeding. Springer; 2008. p. 115-50.
30. Bian Y, Ballington J, Raja A, Brouwer C, Reid R, Burke M, et al. Patterns of simple sequence repeats in cultivated blueberries (Vaccinium section Cyanococcus spp.) and their use in revealing genetic diversity and population structure. Molecular Breeding. 2014;34 2:675-89. doi:10.1007/s11032-014-0066-7.
31. Bolger AM, Lohse M and Usadel B. Trimmomatic: a flexible trimmer for Illumina sequence data. Bioinformatics. 2014;30 15:2114-20. doi:10.1093/bioinformatics/btu170.
32. Jiang H, Lei R, Ding SW and Zhu S. Skewer: a fast and accurate adapter trimmer for next-generation sequencing paired-end reads. BMC Bioinformatics. 2014;15:182. doi:10.1186/1471-2105-15-182.
33. Heydari M, Miclotte G, Demeester P, Van de Peer Y and Fostier J. Evaluation of the impact of Illumina error correction tools on de novo genome assembly. BMC Bioinformatics. 2017;18 1:374. doi:10.1186/s12859-017-1784-8.
34. Yang X, Tschaplinski TJ, Hurst GB, Jawdy S, Abraham PE, Lankford PK, et al. Discovery and annotation of small proteins using genomics, proteomics, and computational approaches. Genome Res. 2011;21 4:634-41. doi:10.1101/gr.109280.110.
35. Ramirez-Sanchez O, Perez-Rodriguez P, Delaye L and Tiessen A. Plant Proteins Are Smaller Because They Are Encoded by Fewer Exons than Animal Proteins. Genomics Proteomics Bioinformatics. 2016;14 6:357-70. doi:10.1016/j.gpb.2016.06.003.
36. Hoang NV, Furtado A, Mason PJ, Marquardt A, Kasirajan L, Thirugnanasambandam PP, et al. A survey of the complex transcriptome from the highly polyploid sugarcane genome using full-length isoform sequencing and de novo assembly from short read sequencing. BMC Genomics. 2017;18 1:395. doi:10.1186/s12864-017-3757-8.
37. Visser EA, Wegrzyn JL, Steenkmap ET, Myburg AA and Naidoo S. Combined de novo and genome guided assembly and annotation of the Pinus patula juvenile shoot transcriptome. BMC Genomics. 2015;16:1057. doi:10.1186/s12864-015-2277-7.
38. Del Fabbro C, Scalabrin S, Morgante M and Giorgi FM. An extensive evaluation of read trimming effects on Illumina NGS data analysis. PLoS One. 2013;8 12:e85024. doi:10.1371/journal.pone.0085024.
39. Duan J, Xia C, Zhao G, Jia J and Kong X. Optimizing de novo common wheat transcriptome assembly using short-read RNA-Seq data. BMC Genomics. 2012;13:392. doi:10.1186/1471-2164-13-392.

40. Chandra S, Singh D, Pathak J, Kumari S, Kumar M, Poddar R, et al. De Novo Assembled Wheat Transcriptomes Delineate Differentially Expressed Host Genes in Response to Leaf Rust Infection. PLoS One. 2016;11 2:e0148453. doi:10.1371/journal.pone.0148453.

41. Chow KS, Ghazali AK, Hoh CC and Mohd-Zainuddin Z. RNA sequencing read depth requirement for optimal transcriptome coverage in Hevea brasiliensis. BMC Res Notes. 2014;7:69. doi:10.1186/1756-0500-7-69.

42. Martin JA and Wang Z. Next-generation transcriptome assembly. Nat Rev Genet. 2011;12 10:671-82. doi:10.1038/nrg3068.

43. Li H and Homer N. A survey of sequence alignment algorithms for next-generation sequencing. Briefings in Bioinformatics. 2010;11 5:473-83. doi:10.1093/bib/bbq015.

44. Smith TF and Waterman MS. Identification of common molecular subsequences. J Mol Biol. 1981;147 1:195-7.

45. Myers G. A fast bit-vector algorithm for approximate string matching based on dynamic programming. Journal of the Acm. 1999;46 3:395-415. doi:Doi 10.1145/316542.316550.

46. Andrews S: FastQC. http://www.bioinformatics.babraham.ac.uk/projects/fastqc/.

47. Trapnell C, Hendrickson DG, Sauvageau M, Goff L, Rinn JL and Pachter L. Differential analysis of gene regulation at transcript resolution with RNA-seq. Nat Biotechnol. 2013;31 1:46-53. doi:10.1038/nbt.2450.

48. Patro R, Duggal G, Love MI, Irizarry RA and Kingsford C. Salmon provides fast and bias-aware quantification of transcript expression. Nat Methods. 2017;14 4:417-9. doi:10.1038/nmeth.4197.

49. Li B, Fillmore N, Bai Y, Collins M, Thomson JA, Stewart R, et al. Evaluation of de novo transcriptome assemblies from RNA-Seq data. Genome Biol. 2014;15 12:553. doi:10.1186/s13059-014-0553-5.

50. Wu TD, Reeder J, Lawrence M, Becker G and Brauer MJ. GMAP and GSNAP for Genomic Sequence Alignment: Enhancements to Speed, Accuracy, and Functionality. Methods Mol Biol. 2016;1418:283-334. doi:10.1007/978-1-4939-3578-9_15.

51. Quinlan AR and Hall IM. BEDTools: a flexible suite of utilities for comparing genomic features. Bioinformatics. 2010;26 6:841-2. doi:10.1093/bioinformatics/btq033.

52. Jay JJ, Eblen JD, Zhang Y, Benson M, Perkins AD, Saxton AM, et al. A systematic comparison of genome-scale clustering algorithms. BMC Bioinformatics. 2012;13 Suppl 10:S7. doi:10.1186/1471-2105-13-S10-S7.

53. Haas B and Papanicolaou A: TransDecoder (Find Coding Regions Within Transcripts). https://transdecoder.github.io/.

54. Altschul SF, Gish W, Miller W, Myers EW and Lipman DJ. Basic local alignment search tool. J Mol Biol. 1990;215 3:403-10. doi:10.1016/S0022-2836(05)80360-2.

55. Finn RD, Coggill P, Eberhardt RY, Eddy SR, Mistry J, Mitchell AL, et al. The Pfam protein families database: towards a more sustainable future. Nucleic Acids Research. 2016;44 D1:D279-D85. doi:10.1093/nar/gkv1344.

56. HMMER 3.1b2. http://hmmer.org/.

57. BroadInstitute: Picard Tools. https://github.com/broadinstitute/picard (2017).

58. DeLuca DS, Levin JZ, Sivachenko A, Fennell T, Nazaire MD, Williams C, et al. RNA-SeQC: RNA-seq metrics for quality control and process optimization. Bioinformatics. 2012;28 11:1530-2. doi:10.1093/bioinformatics/bts196.
59. Anders S, Pyl PT and Huber W. HTSeq-a Python framework to work with high-throughput sequencing data. Bioinformatics. 2015;31 2:166-9. doi:10.1093/bioinformatics/btu638.
60. Langmead B and Salzberg SL. Fast gapped-read alignment with Bowtie 2. Nature Methods. 2012;9 4:357-U54. doi:10.1038/Nmeth.1923.
61. Lunter G and Goodson M. Stampy: A statistical algorithm for sensitive and fast mapping of Illumina sequence reads. Genome Research. 2011;21 6:936-9. doi:10.1101/gr.111120.110.
62. Dobin A, Davis CA, Schlesinger F, Drenkow J, Zaleski C, Jha S, et al. STAR: ultrafast universal RNA-seq aligner. Bioinformatics. 2013;29 1:15-21. doi:10.1093/bioinformatics/bts635.
63. Kim D, Langmead B and Salzberg SL. HISAT: a fast spliced aligner with low memory requirements. Nat Methods. 2015;12 4:357-60. doi:10.1038/nmeth.3317.

Supplementary data

Figure S1

.jpg

Diagram representing the *de novo* assembly strategies, run independently for each *Vaccinium* species. The set of control and treatment reads produced by different correction and trimming strategies were used as input. The control read files were assembled (A) independently as were the treatment read files (B). From here, each set of control sample transcripts was combined with the treatment sample transcripts (i.e. the Skewer corrected control transcripts were merged with the Skewer corrected treatment transcripts, the Trimmomatic uncorrected control transcripts were merged with the Trimmomatic uncorrected treatment transcripts, etc.) (C). These merged transcript sets were then clustered with either CD-HIT (D) or RapClust (E). This results in eight clustered assemblies. A second assembly strategy merged the control and treatment reads prior to assembly (F). These sets of transcripts were also clustered with either CD-HIT (G) or RapClust (H), also resulting in another set of eight clustered assemblies.

Figure S2

.tiff

Subdivision in categories of reads mapped to the reference genome performed by HTSeq. Except in the case of STAR, which does not report not mapped reads, height of bars up to red resembles the number of trimmed reads. Options are ordered by correction state, mismatch tolerance options and trimming software.

Figure S3

.tiff

Mapping results to the reference genome categorized by overlapping gene feature.

Figure S4

.pdf

Subdivision in categories of reads mapped to *de novo* assemblies performed by HTSeq. In specific cases with HISAT2 and STAR, multiple aligned reads are counted multiple times, overestimating the total number of reads. Options are ordered by correction state, trimming software and type of assembly.

Table S1

.xlsx

Variation in number and length of reads after pre-processing.

Number of reads before and after trimming with either Skewer or Trimmomatic and using (cor) or not (Uc) error correction. Last column indicate average length of reads after trimming the 101-bp raw reads. Values are mean ± sd of 8 samples.

Table S2

.xslx

Combination of clustering results of *de novo* assemblies with transcript lengths.

Distribution of the number of sequences within each cluster (CLUSSEQS) and length of the transcript sequences (LEN). FILTSEQS indicate transcripts filtered out due to low read support by RapClust.

Table S3

.xslx

Read mapping rates. Proportion of reads mapped from each combination of error correction, trimming software, mismatch tolerance or assembly samples, when appropriate, to either the reference genome or *de novo* assemblies after clustering with CD-HIT.

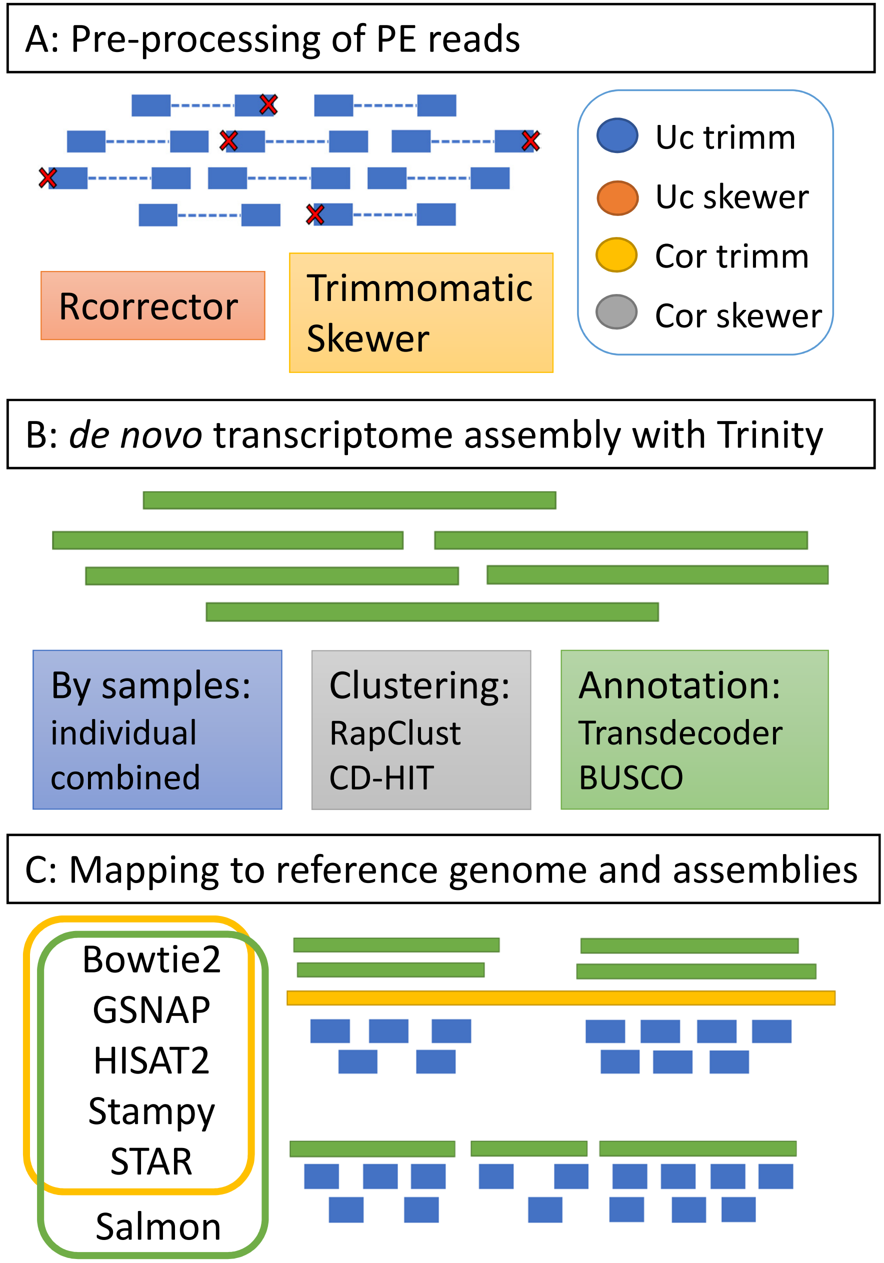
A: Pre-processing of PE reads
Uc trimm
Uc skewer
Cor trimm
Cor skewer
Rcorrector
Trimmomatic
Skewer
B: de novo transcriptome assembly with Trinity
By samples:
individual
combined
Clustering:
RapClust
CD-HIT
Annotation:
Transdecoder
BUSCO
C: Mapping to reference genome and assemblies
Bowtie2
GSNAP
HISAT2
Stampy
STAR
Salmon

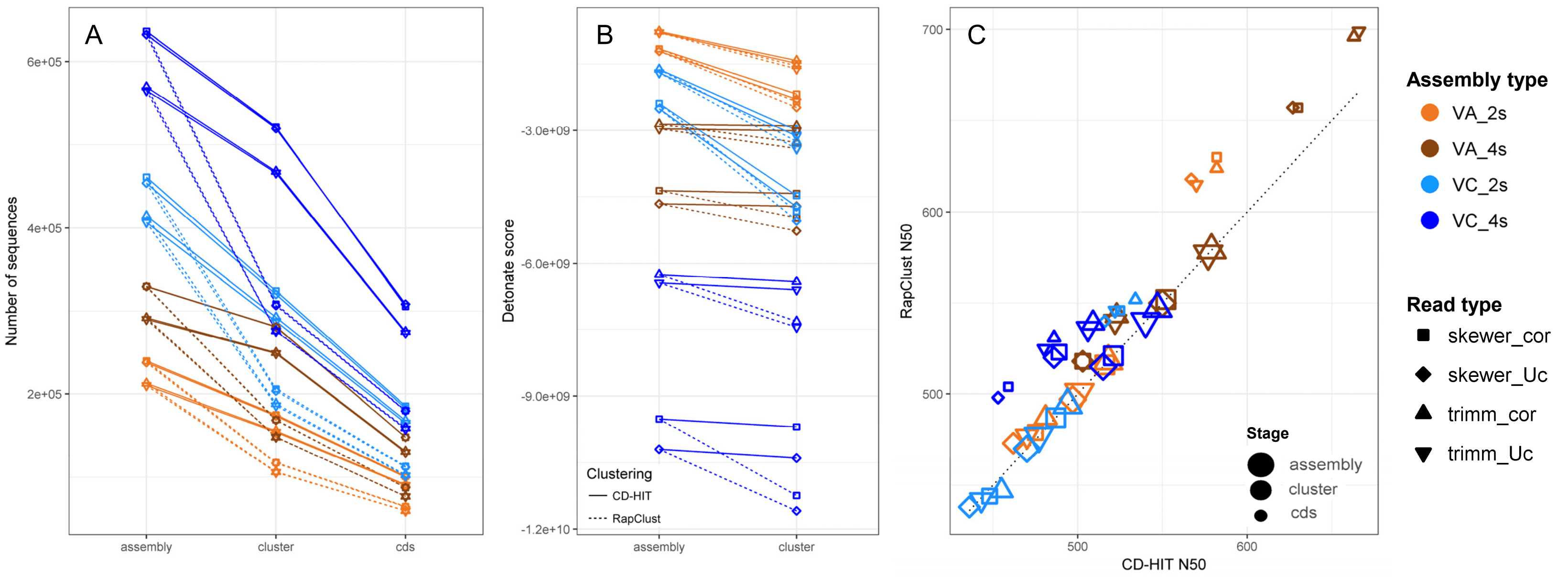

A
Number of sequences
6e+05
4e+05
2e+05
assembly
cluster
cds
B
Detonate score
-3.0e+09
-6.0e+09
-9.0e+09
-1.2e+10
Clustering
CD-HIT
RapClust
assembly
cluster
C
RapClust N50
700
600
500
CD-HIT N50
Stage
assembly
cluster
cds
Assembly type
VA_2s
VA_4s
VC_2s
VC_4s
Read type
skewer_cor
skewer_Uc
trimm_cor
trimm_Uc

Click here to download Figure Fig 3.tiff

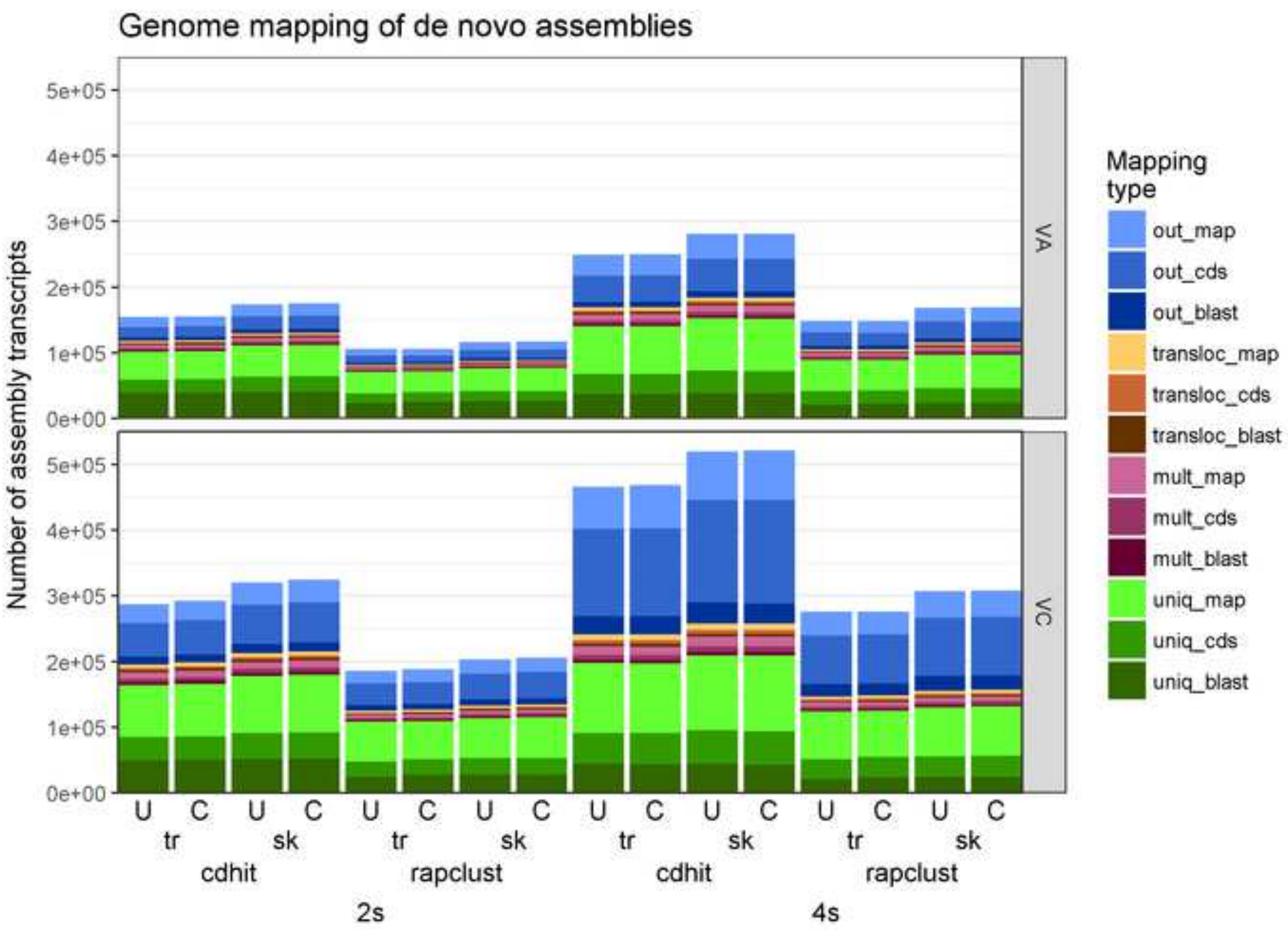

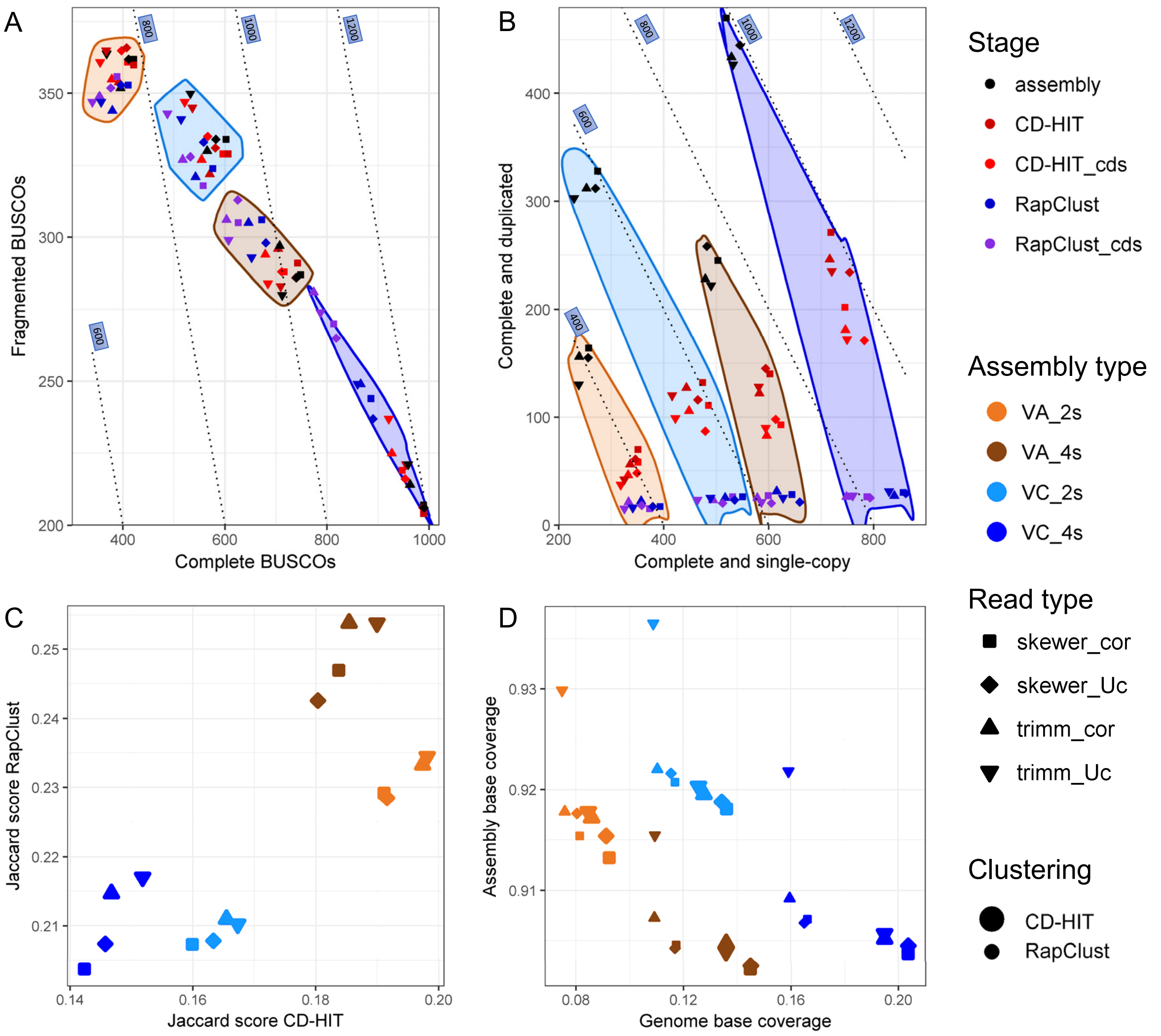

A
B
C
D
Fragmented BUSCOs
Complete BUSCOs
Complete and duplicated
Complete and single-copy
Jaccard score RapClust
Jaccard score CD-HIT
Assembly base coverage
Genome base coverage
600
800
1000
1200
400
Stage
assembly
CD-HIT
CD-HIT_cds
RapClust
RapClust_cds
Assembly type
VA_2s
VA_4s
VC_2s
VC_4s
Read type
skewer_cor
skewer_Uc
trimm_cor
trimm_Uc
Clustering
CD-HIT
RapClust

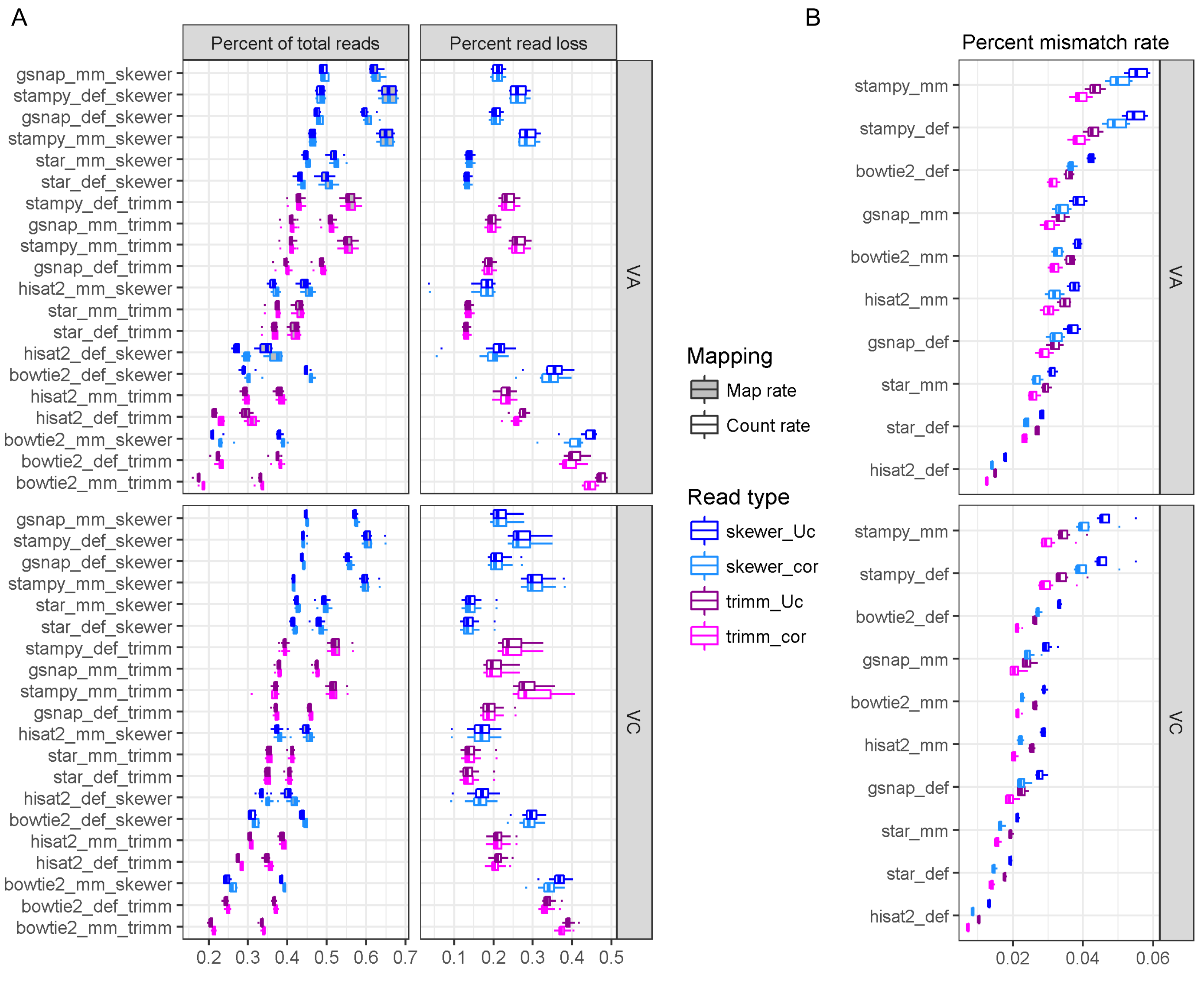

A
B
Percent of total reads
Percent read loss
Percent mismatch rate
gsnap_mm_skewer
stampy_def_skewer
gsnap_def_skewer
stampy_mm_skewer
star_mm_skewer
star_def_skewer
stampy_def_trimm
gsnap_mm_trimm
stampy_mm_trimm
gsnap_def_trimm
hisat2_mm_skewer
star_mm_trimm
star_def_trimm
hisat2_def_skewer
bowtie2_def_skewer
hisat2_mm_trimm
hisat2_def_trimm
bowtie2_mm_skewer
bowtie2_def_trimm
bowtie2_mm_trimm
stampy_mm
stampy_def
bowtie2_def
gsnap_mm
bowtie2_mm
hisat2_mm
gsnap_def
star_mm
star_def
hisat2_def
VA
VC
Mapping
Map rate
Count rate
Read type
skewer_Uc
skewer_cor
trimm_Uc
trimm_cor
0.2 0.3 0.4 0.5 0.6 0.7
0.1 0.2 0.3 0.4 0.5
0.02 0.04 0.06

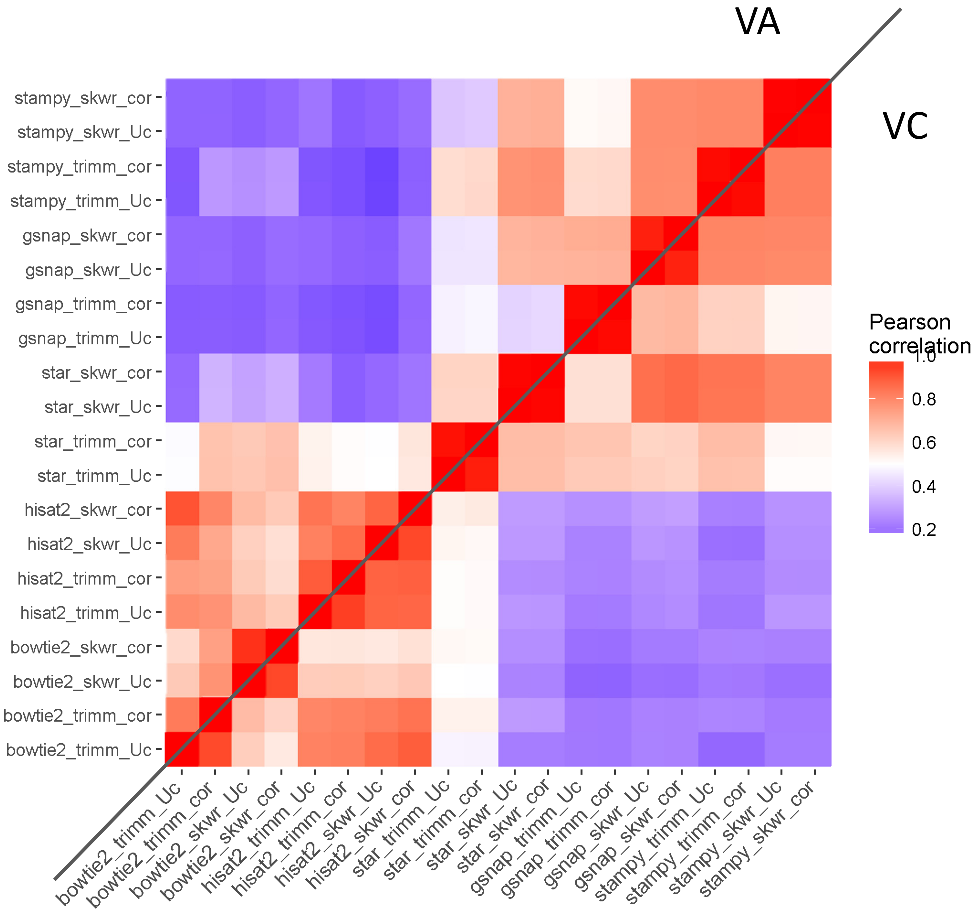
VA
VC
Pearson correlation
1.0
0.8
0.6
0.4
0.2
stampy_skwr_cor
stampy_skwr_Uc
stampy_trimm_cor
stampy_trimm_Uc
gsnap_skwr_cor
gsnap_skwr_Uc
gsnap_trimm_cor
gsnap_trimm_Uc
star_skwr_cor
star_skwr_Uc
star_trimm_cor
star_trimm_Uc
hisat2_skwr_cor
hisat2_skwr_Uc
hisat2_trimm_cor
hisat2_trimm_Uc
bowtie2_skwr_cor
bowtie2_skwr_Uc
bowtie2_trimm_cor
bowtie2_trimm_Uc

Click here to download Figure Fig 7.tiff

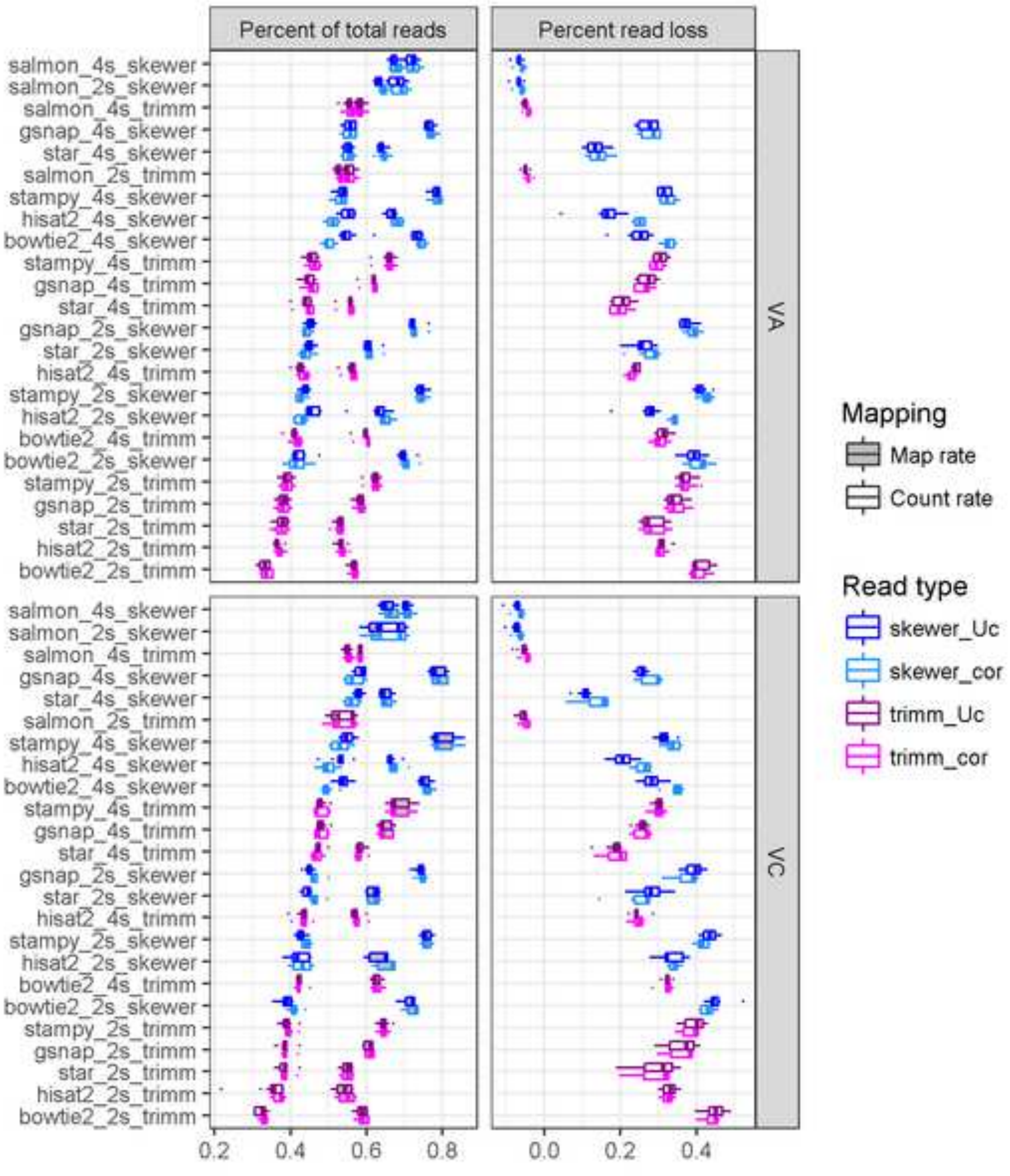

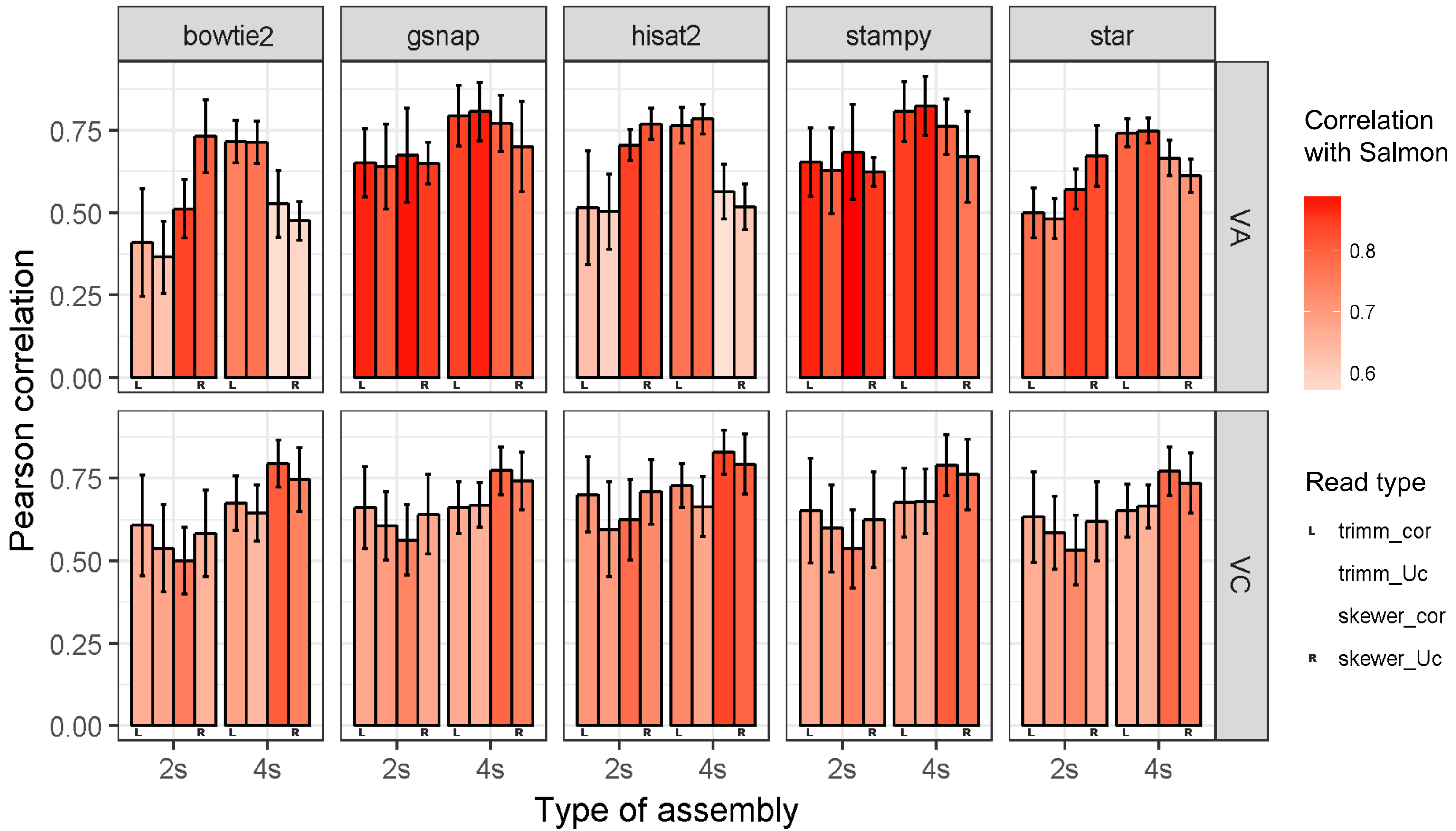
bowtie2
gsnap
hisat2
stampy
star
VA
VC
Pearson correlation
0.00
0.25
0.50
0.75
L
R
2s
4s
Type of assembly
Correlation with Salmon
0.6
0.7
0.8
Read type
trimm_cor
trimm_Uc
skewer_cor
skewer_Uc

Click here to access/download
Supplementary Material
Fig S1.jpg

Click here to access/download
Supplementary Material
Fig S2.tiff

Click here to access/download
Supplementary Material
Fig S3.tiff

Click here to access/download
Supplementary Material
Fig S4.tiff

Click here to access/download
Supplementary Material
Table S1 reads.xlsx

Click here to access/download
Supplementary Material
Table S2.xlsx

Click here to access/download
Supplementary Material
Table S3 map.xlsx
