# Supplementary material for: Comprehensive evaluation of RNA-seq analysis pipelines in diploid and polyploid species
Source: Gigascience. 2018 Nov 10;7(12):giy132. doi: 10.1093/gigascience/giy132 (PMC6275443; doi:10.1093/gigascience/giy132)
Supplement: GIGA-D-18-00140_Revision_1.pdf [file giy132_giga-d-18-00140_revision_1.pdf]

GigaScience

Comprehensive evaluation of RNA-Seq analysis pipelines in diploid and polyploid species

--Manuscript Draft--

Manuscript Number:	GIGA-D-18-00140R1
Full Title:	Comprehensive evaluation of RNA-Seq analysis pipelines in diploid and polyploid species
Article Type:	Research
Funding Information:	National Institute of Food and Agriculture (2009–02533) — Dr. Margaret Staton<br>Agricultural Research Service (58–6062–5-004) — Dr. Margaret Staton
Abstract:	Background<br><br>The usual analysis of RNA-Seq reads is based on an existing reference genome and annotated gene models. However, when a reference for the sequenced species is not available, alternatives include using a reference genome from a related species or reconstructing transcript sequences with de novo assembly. In addition, researchers are faced with many options for RNA-Seq data processing and limited information on how their decisions will impact the final outcome. Using both a diploid and polyploid species with a distant reference genome, we have tested the influence of different tools at various steps of a typical RNA-Seq analysis workflow on the recovery of useful processed data available for downstream analysis.<br><br>Findings<br><br>At the preprocessing step, we found error correction has a strong influence on de novo assembly but not on mapping results. After trimming, a greater percentage of reads were able to be used in downstream analysis by selecting gentle quality trimming performed with Skewer instead of strict quality trimming with Trimmomatic. This availability of reads correlated with size, quality and completeness of de novo assemblies, and number of mapped reads. When selecting a reference genome from a related species to map reads, outcome was significantly improved when using mapping software tolerant of greater sequence divergence, such as Stampy or GSNAP.<br><br>Conclusions<br><br>The selection of bioinformatic software tools for RNA-Seq data analysis can maximize quality parameters on de novo assemblies and availability of reads in downstream analysis.
Corresponding Author:	Margaret E Staton, Ph.D.<br>University of Tennessee Knoxville<br>Knoxville, TN UNITED STATES
Corresponding Author Secondary Information:	
Corresponding Author's Institution:	University of Tennessee Knoxville
Corresponding Author's Secondary Institution:	
First Author:	Miriam Payá-Milans
First Author Secondary Information:	
Order of Authors:	Miriam Payá-Milans<br>James W. Olmstead

Powered by Editorial Manager® and ProduXion Manager® from Aries Systems Corporation

	Gerardo Nunez
	Timothy A. Rinehart
	Margaret Staton
**Order of Authors Secondary Information:**	

Response to Reviewers:

Reviewer reports:

>Reviewer #1: General Comments.

>The idea of comparing different assembly and mapping strategies is compelling. It is true, that there are few resources about the effects of polyplody on tools designed mostly for diploids. Since the mappings are already done, you could explore in more detail how multiple homoeologues may be mapping to the same "unigene", or you could try to figure out if the homoeologues are removed/merged into single unigenes. If that is the case, you may be mapping the tetraploid to a reference closer to a diploid. If the duplication event is recent, you can expect almost double of the genes in the tetraploid transcriptome, compared to the diploid.

We attempted a comparison between transcripts from the tetraploid and diploid gene models, but results were difficult to interpret. To date, there is no tetraploid Vaccinium genome to use for the sequences for homoeolog genes to distinguish between isoforms and homoeologues. Thus, we used the BUSCO tool (benchmark universal single copy orthologs) to explore the relative duplication of transcriptomes, considering that similar homoeologues may be hits to the same BUSCO protein, and also we discuss how clustering reduces duplication; however, whether these duplicates are homoeologues or isoforms remains uncertain. In relation to when the duplication event took place, although cytogenetic studies have been done to assess blueberry ploidy (Sakhanokho 2018), we couldn’t find any information on specific timing (recent or not) in the literature. Figure 5 A&B, Lines 517-520.

>The idea behind figure 1, that shows all the tools is nice. However, it can be improved to make the order of the pipeline more explicit.

Figure 1 is modified and now contains arrows to help follow the pipeline.

>Also, the kind of algorithms, drawbacks, advantages, etc of each program used is scatter all over the place. It would be nice to have a table with all that information summarized, including one column with a short description of the final effect in each step of the analysis. A row could look like (with more rows, one for each step in the pipeline)
>Tool: Trimmomatic
>De Novo Assembly: Improves in 5% on VC (or whatever you find)
>Mapping to genome: Limited effect.

As suggested, a new supplementary table including pre-processing tools, assemblers, clustering methods and aligners has been added. (Table S1)

The figures require a lot of work to make them look consistent (same colours for same variables across the paper, for example).

>Colors have been made consistent among figures.

>Specific comments.

>Figure 1.
>General: It is confusing what are characteristics of the analysis (like individual/combined), programs (Is Rcorrector a program? A typo?). Some colour/font style change could help to distinguish them. The legend requires a lot of work, as it is not very descriptive of the elements represented. Also, the colours could be improved to reduce confusion. Yellow seems to represent "cor trim" and reference genome. Grey is for Cor skewer, but it is also used for Clustering.
>Panel A: Cor skewer is not present in the diagram. Also, there is no explanation of

Powered by Editorial Manager® and ProduXion Manager® from Aries Systems Corporation

	what the crosses mean. Rcorrector is not defined in legend. The figure seems to suggest that Rcorrector and Trimmomatic/Skewer are two different pipelines, where in the text it is described as Rcorrector+Trimmmatic or Rcorrector+Skewer<br>>Panel B: The boxes don't need to be colour coded, as the colours are not used elsewhere to link, and adds confusion as green are blue are used to represent transcripts and reads elsewhere in the figure.<br>>Panel C: It is not clear that the top and the bottom diagrams are different things (De Novo vs reference guided).<br><br>Considering the comments of the reviewer, Figure 1 has been modified and the legend is now fully descriptive.<br><br>>Line 72. Illumina may still be cheaper, but it may be worth mentioning Iso-Seq, from PacBio that are already able to retrieve full transcripts. I understand it is beyond the scope of the paper, but it is worth mentioning.<br><br>A line commenting on Iso-Seq for transcriptome studies is added (Line 85-88).<br><br>>Line 89. A supplementary figure showing how the different errors affect the assembly could help the unexperienced reader to understand why the errors happen.<br><br>A short description and an additional citation are included to help readers with this (Line 104-105).<br><br>>Line 93. It is commonly selected, agreed. But how do you define good performance? Having used it before, the pipeline writes several temporary files, which computationally is not very performant. If it refers to the quality of the assembly, no other options are discussed in this paper, are there any other RNA-Seq assemblers?<br><br>Our original goal for good performance was referring to high scores in metrics such as mapped-back reads,fewer chimeras, or good recovery of transcripts, where Trinity performs well. The review makes a good point that performance may be related to computational efficiency rather than or in addition to biological accuracy, “good performance” is changed to “good quality”. (Line 112). Also, a pair of extra assemblers are added to the analysis as requested by another reviewer, please see below.<br><br>>Line 112. FM-Index is not defined. Hash tables are considered fast in computer science. You can argue that it depends on details of the implementations and how the different software compensate for the drawbacks (like doing a "proper " alignment once the region where the read maps is identified).<br><br>The description has been added, and also the sentence was modified to indicate array and algorithm on the comparison. (Line 135-138)<br><br>>Line 136: Is Vaccinium corymbosum derived from a duplication of V. arboreum? if so, it may be worth to mention. It would also be nice to have a comparison of how distant they are.<br><br>V. corymbosum is considered autotetraploid, derived from a duplication of a diploid V. corymbosum (not a hybridization). A diploid V. corymbosum individual was used for genome sequencing; this is now indicated right after the informtion that VC is autotetraploid. (Line 158-162) V. arboreum is a different species in a different section of Vaccinium, now specified in the text. (Line 164-165). While some limited phylogenetics analysis of Vaccinium spp. has been completed, none include both the species we used for this study.<br><br>>Table S1. Add more detailed columns, so besides the column with the name, you have a description. So, VC_trimm_Uc can have a three extra columns explaining VC, trim and Uc. May seem redundant, but it will allow to interpret the table on its own.<br><br>As suggested, the extra columns are added.<br><br>>Line 157. How do you decide if it is significant?

Powered by Editorial Manager® and ProduXion Manager® from Aries Systems Corporation

A statistician was consulted during the interpretation of results, but because the statistical report did not contain all possible options, we decided not to include it. Significant is changed to low. (Line 182)

>Figure S1. You can coordinate the colours of the samples with the legend on Figure 1, to make everything consistent.

Colors have been made more consistent between the figures and to the rest of the figures in the paper.

>Line 196/Table 1. I would suggest to move this table to supplementals and show a boxplot with the size of the assemblies for each donation.

This table has been moved to the supplemental materials.

>Line 240. Detonate has not been described in the introduction, where other tools had been mentioned and how they work.

The tools mentioned in the introduction are all used in head-to-head comparisons. Tools used only to calculate metrics were not mentioned. However, it is a good idea to explain more about Detonate in the results. A sentence about it and the reference are now added to the Analysis section. (Line 217-222)

>Lile 272: Be consistent with the nomenclature. In the figure it is marked as "VC_4" and on text as "VC 4". You can rename the columns on your tables before plotting with something like: gsub("_"," ", table$Assmbly_type) if you are using R.

The underscores on assembly type in figures are removed.

>Figure 3. The "transloc" and mult "bands" are hard to read, probably have this a supplemental table. I would also normalize the plot in percentages and have an extra panel with the number of transcripts that are used.

Mapping results are now provided as a table. Leaving total number of transcripts in the figure instead of using percents is intentional to visualize the global variations, and also, its not clear if it would be more informative to look at percents of total reads mapped or of total reads sequenced. However, to provide readers with either option, we have added the percentages in the supplemental file. This figure is now updated to improve compactness and visualization.

>Paragraph starting on Line 337: So from this paragraph, we may conclude that it is more important the number and volume of reads than the data processing? Maybe it would be worth to consider if the cost of sequencing more is cheaper than having more steps in the analysis? Or full transcript sequencing?

From these results, the suggestion is that if you have sequenced multiple samples, combining them may perform better than using them separately. Also, soft trimming has a positive effect. We find it to be impossible to estimate if the cost of analysis, which largely depends on the type of bioinformatics support available for each research group. Full transcript sequencing (IsoSeq) may help assembly, although this type of sequencing has higher error and requires error correction. Without testing we prefer not to make further suggestions about this method. Instead, we mention IsoSeq as an alternative method in the introduction (line 85-89).

>Paragraph starting on line 486: Did you evaluate how homoeologue genes affect the mapping? I'm wondering if during the clustering step you could be collapsing homoeologues in a single representation.

Current genomic resources in blueberry, like in most polyploids, do not include precise information on homoeolog sequences. As such, transcripts produced from homoeologues with less than 5% sequence variation, would be collapsed by CD-HIT, which affected 22% of sequences with very little effect on quality metrics. Considering the soft clustering method applied and high similarity of putative collapsed homoeologues, the global effect on read mapping is expected to be low. A sentence

mentioning this is added at the beginning of the section (Lines 548-550). Specific to assembly clustering, possible collapse of homoeologs by clustering is mentioned as well (Line 517-520).

>Methods.

>Are the scripts/exact commands used for the analysis deposited somewhere? You could have a GitHub repository with your scripts or add them as supplemental (or both!)

Most of the work consisted of running external software on the command line. Basic instructions on how to run these are included in the manuscript. For some specific functions written by the authors, including the calculations of Jaccard scores and coverage, a package of scripts was submitted to Gigascience and will be available as part of the publication through an ftp link. This should also be provided to the reviewers.

>List of abbreviations: Include all the abbreviations used, like "cor", "trim", etc.

Following the suggestion, the list has been updated.

>Reviewer #2: Major Concern:
>The authors benchmarked Control Reads against Treatment Reads, Single Sample against Multiple Samples as input, CD-HIT against RapClust for clustering, and five mappers including bowtie2, gsnap, stampy, star and hisat2 for mapping reads. But for assembly, the authors benchmarked only one transcriptome assembler, Trinity.

We now included three assemblers, see below.

>The authors claimed, "Trinity is commonly selected and has good performance" in line 94 and cited two papers. One paper titled "Optimizing de novo transcriptome assembly …" was published 2011, which is a bit outdated and doesn't include the benchmark of latest short-read transcriptome assemblers. The other paper "Comprehensive evaluation of de novo …" is new (2017) but doesn't support the authors claim and concluded in its abstract, quote: "SOAPdenovo-Trans performed best in base coverage, while Trans-ABySS performed best in gene coverage and number of recovered full-length transcripts. In terms of chimeric sequences, BinPacker and Oases-Velvet were the worst, while IDBA-tran, SOAPdenovo-Trans, Trans-ABySS and Trinity produced fewer chimeras across all single k-mer assemblies."

The claim of "good performance" is modified to "usually good quality", which is not contradicted with the references considering that in both of them, Trinity was best or second best at some quality metrics.

>As we know, transcriptome assemblers perform differently on genomes of different characteristics - Trinity usually performs better on mammals and vertebrates, SOAPdenovo-Trans on plants and Trans-ABySS on metagenomics. As the authors are targeting a "Comprehensive evaluation of RNA-Seq analysis pipelines", it is necessary to include another one or two leading transcriptome assemblers.

A comparison including assemblies from SOAPdenovo-Trans (due to the indicated usual better performance on plants, which we were not aware of), Trans-AbySS (which had also good performance in the references), and Trinity has been added.

>Minor Concerns:
>Cite Detonate score paper in line 240.

Citation was added.

**Additional Information:**	
**Question**	**Response**

Powered by Editorial Manager® and ProduXion Manager® from Aries Systems Corporation

Are you submitting this manuscript to a special series or article collection?	No
**Experimental design and statistics**<br><br>Full details of the experimental design and statistical methods used should be given in the Methods section, as detailed in our Minimum Standards Reporting Checklist. Information essential to interpreting the data presented should be made available in the figure legends.<br><br>Have you included all the information requested in your manuscript?	Yes
**Resources**<br><br>A description of all resources used, including antibodies, cell lines, animals and software tools, with enough information to allow them to be uniquely identified, should be included in the Methods section. Authors are strongly encouraged to cite Research Resource Identifiers (RRIDs) for antibodies, model organisms and tools, where possible.<br><br>Have you included the information requested as detailed in our Minimum Standards Reporting Checklist?	Yes
**Availability of data and materials**<br><br>All datasets and code on which the conclusions of the paper rely must be either included in your submission or deposited in publicly available repositories (where available and ethically appropriate), referencing such data using a unique identifier in the references and in the "Availability of Data and Materials" section of your manuscript.<br><br>Have you have met the above requirement as detailed in our Minimum	Yes

Standards Reporting Checklist?	

Powered by Editorial Manager® and ProduXion Manager® from Aries Systems Corporation

Manuscript w DOI
Click here to view linked References
Click here to access/download;Manuscript;workflow paper revised upload version w DOI.docx

Comprehensive evaluation of RNA-Seq analysis pipelines in diploid and polyploid species

Miriam Payá-Milans[1], James W. Olmstead[2], Gerardo Nunez[2], Timothy A. Rinehart[3,4], Margaret Staton[1*]

Affiliations for authors:

[1] Department of Entomology and Plant Pathology, University of Tennessee

[2] Horticultural Sciences Department, University of Florida

[3] Thad Cochran Southern Horticultural Laboratory, USDA-Agricultural Research Service

[4] Crop Production and Protection, USDA-Agricultural Research Service

emails:

Miriam Payá-Milans (MPM): mmilans@utk.edu

James W. Olmstead (JO): james.olmstead@driscolls.com

Gerardo Nunez (GN): g.nunez@ufl.edu

Timothy A. Rinehart (TR): tim.rinehart@ars.usda.gov

Margaret Staton (MS): mstaton1@utk.edu

*Corresponding author:

E-mail: mstaton1@utk.edu

Abstract

Background: The usual analysis of RNA-Seq reads is based on an existing reference genome and annotated gene models. However, when a reference for the sequenced species is not available, alternatives include using a reference genome from a related species or reconstructing transcript sequences with *de novo* assembly. In addition, researchers are faced with many options for RNA-Seq data processing and limited information on how their decisions will impact the final outcome. Using both a diploid and polyploid species with a distant reference genome, we have tested the influence of different tools at various steps of a typical RNA-Seq analysis workflow on the recovery of useful processed data available for downstream analysis.

Findings: At the preprocessing step, we found error correction has a strong influence on *de novo* assembly but not on mapping results. After trimming, a greater percentage of reads were able to be used in downstream analysis by selecting gentle quality trimming performed with Skewer instead of strict quality trimming with Trimmomatic. This availability of reads correlated with size, quality and completeness of *de novo* assemblies, and number of mapped reads. When selecting a reference genome from a related species to map reads, outcome was significantly improved when using mapping software tolerant of greater sequence divergence, such as Stampy or GSNAP.

Conclusions: The selection of bioinformatic software tools for RNA-Seq data analysis can maximize quality parameters on *de novo* assemblies and availability of reads in downstream analysis.

Keywords: RNA-Seq, pipeline, polyploid, correction, trimming, assembly, clustering, reference genome, mapping

Background

Bioinformatics is a field under constant expansion with regular advances in the development of software and algorithms. This requires researchers to continuously evaluate available software tools and approaches to maximize accuracy of experimental outcomes [1]. However, the majority of the relevant studies comparing bioinformatic tools for RNA-Seq data focus on straightforward scenarios with diploid eukaryotes with an available reference genome [2-5]. The implications of data analysis decisions are less clearly understood in situations where, for example, the species of interest is a polyploid or the species of interest does not have a reference genome but a reference genome is available from a sister clade. This study aims to explore RNA-Seq data analysis from this scenario, where the main steps are read trimming, either mapping to a related species reference genome (from here on referred to as a "distant reference") or to a *de novo* transcriptome assembly, and read quantification by gene or transcript (Figure 1). Moreover, this study compares decisions along the RNA-Seq analysis steps of a workflow, examining all permutations of those decisions from the beginning to the end of the pipeline.

Figure 1. Schematic view of the RNA-Seq pipeline followed on this work.
(A) Samples were obtained from roots of the diploid *Vaccinium arboreum* (VA) and tetraploid *V. corymbosum* (VC) grown at either pH 4.5 or 6.5, and sequenced. (B) Paired-end (PE) Illumina reads were either error corrected (cor; black lines) or not (Uc), and trimmed for removal of adapters and either low-quality bases (trimm; red crosses) or not (skewer). (C) Each set of reads was subjected to two *de novo* transcriptome assembly methods (2 individual samples and merge results, or 4 combined samples) with three assemblers, followed by redundancy reduction by CD-HIT and RapClust clustering methods. Metrics were conducted on all steps. Trinity transcriptomes were further annotated, and their CD-HIT clusters used for mapping (underlined). (D) Transcripts were mapped to a diploid VC genome with gmap for mapping metrics, while short reads were mapped to either the genome or a transcriptome using multiple read aligners to obtain read counts.

From the many next generation sequencing platforms that generate RNA-Seq data, Illumina has had the greatest success, yielding high quality reads at a reasonable price and read length increasing with new generations of instruments [6]. An alternative to RNA-Seq for the study of transcriptomes is Iso-Seq, a method developed by PacBio to analyze molecules 1-6 Kb long. This method has the advantage of capturing full transcripts but is significantly more expensive per base and thus currently less commonly used than RNA-Seq [7]. From raw RNA-Seq reads, numerous informatic analysis decisions must be made to derive meaningful biological data, starting with any preprocessing of the reads. Despite the usually high accuracy of Illumina reads (0.1% error rate), error correction is a method with potential to improve the quality of read alignment and *de novo* assembly [8]. Before sequencing, adapters are incorporated to both ends of each sequence. Trimming of bases originating from these adapters is required, but the merit of aggressive versus gentle trimming of lower quality bases, which modifies the final amount of data, is still being explored [9].

After preprocessing, if a reference genome is available, RNA-Seq reads may be used to call variants or determine differentially expressed genes; on the contrary, *de novo* assembly may be used to reconstruct transcripts to do such analyses [10]. *De novo* transcriptome assembly in plants is complex due to the sequence similarity of transcripts that are isoforms, paralogs, orthologs and, in the case of polyploids, homoeologs. Moreover, in transcriptomes of plants under environmental stress, alternative splicing is even more prevalent [11]. During *de novo* assembly, this complexity is reflected in the form of bubbles or extra branches in de Bruijn graphs that may lead to imperfect assemblies, with a portion of assembled transcripts affected by artifacts such as hybrid assembly of gene families, transcript fusion (chimerism), insertions in contigs, and structural abnormalities such as incompleteness, fragmentation, and local misassembly of contigs [12, 13].

From the many assemblers developed to use with short reads, Trinity [14] is often selected and usually produces good quality assemblies at single *k*-mer [4, 15]. Trans-ABySS [16], which has good recovery of full transcripts, and SOAPdenovo-Trans [17], designed to handle difficulties of plant genes assembly, are also prevalent. A next step to refine *de novo* assemblies is often to further reduce transcript redundancy. One

popular tool is CD-HIT [18], which removes shorter redundant sequences based on sequence similarity. A more recently released clustering tool, RapClust [19], generates clusters based on the relationships exposed by multi-mapping sequencing fragments and is considerably faster than previous approaches. Several methods are available to assess the overall quality, accuracy, contiguity and completeness of a *de novo* assembled transcriptome, including basic metrics for assemblies, contig-level metrics, reference-free evaluation methods that include read support, and comparison to protein datasets from related species [10, 12, 20-22].

Read mapping is a crucial step to estimate gene expression for further analysis, but is made difficult by sequencing errors and is dependent on characteristics of the reference (quality of gene annotation, relatedness to sequenced individuals, size, repetitive regions, ploidy, etc.) [23]. Mapping transcript reads to a reference genome has the additional challenge of crossing splice junctions, some of which may not be accurately annotated [3]. Multiple metrics can be used to determine performance of read aligners. Precision and recall are the usual metrics with simulated data, while evaluations without *a priori* known outcomes utilize mapping rate, base mismatch rate, detected transcripts or correlation of gene expression estimates to quantify performance [2, 24]. These outcomes are dependent on the individual implementations of each alignment software package. Many short read aligners are based on hash tables, with quick seeding of alignment candidates and alignment extension with precise algorithms. These are more sensitive but usually slower than those based on the ultrafast FM-index (Full-text index in Minute space) and extension by dynamic programming, which are fast though less flexible with handling errors [2, 10]. When using a distant genome, sequence divergence between reads and the reference genome may compromise results; nucleotide mismatches are more likely to decrease the number of mapped reads, while indels are usually better tolerated with gapped alignments [2]. One benefit from the utilization of a distant genome is a direct comparison of gene expression results from multiple related species [25]. On the other hand, utilization of *de novo* assemblies avoids the mapping issues to a distant genome and also captures divergent and novel genes useful for species-specific discovery of new functions. Selecting between a *de novo* transcriptome or a reference genome has been shown to produce comparable gene

expression profiles at over 87% correlation in other systems but has not been examined in plants [5, 24].

Most prior papers examining the choice of informatics software for RNA-Seq data analysis worked with straightforward data sets, either performing a single type of analysis on the data or working with data from diploid organisms with well-developed reference genomes. However, much less research has been done into genomics of complex species and, especially in the case of plants, polyploids. Many polyploid crops now have available reference genomes, like strawberry [26], cotton [27], wheat [28], or sweet potato [29], while others continue to rely on genomic resources from diploid relatives, such as potato [30], kiwifruit [31], peanut [32], or blueberry [33]. Here, we have selected blueberry datasets as an example. A number of different species of blueberries are used in agricultural production and breeding, with autotetraploid *Vaccinium corymbosum* (highbush blueberry) as the most economically important [34]. A diploid accession of *V. corymbosum* was used for genome sequencing and construction of a blueberry reference genome [33, 35]. In this study we use RNA-Seq data from an autotetraploid *V. corymbosum* (section *Cyanococcus*) and a diploid species, *V. arboreum* (section *Batodendron*).

Data description

The sequencing data used in this work is 270 million Illumina paired-end reads (2*101 bp long) for diploid *V. arboreum* (VA) and 582 million reads for tetraploid *V. corymbosum* (VC), originating from 8 plants each [25] and sequenced on duplicate lanes. Libraries were prepared from RNA collected from roots of plants of similar age after eight weeks of growth in hydroponic systems under either stressful (pH 6.5) or control (pH 4.5) conditions. All sequence data is publicly available at NCBI (see details below). At the first step of data curation, our tested methods are error correction of RNA-Seq data with Rcorrector and trimming of low quality bases by one of two methods, Trimmomatic [36] or Skewer [37] (Table S1). Error correction of raw reads modified an average of 0.7% bases per library, a proportion larger than the expected 0.1% sequencing error rate in Illumina reads and suggests a possible masking of variability in the data. Next, both original and corrected reads were trimmed using either

Skewer or Trimmomatic at default settings. Gentle quality trimming with Skewer retained on average 99.6% reads at mean length 99.8 bp (Table S2). In contrast, quality trimming with Trimmomatic, which has significantly more aggressive default trimming parameters, retained 77.2% of reads at mean length 93.8 bp. Error correction had a low effect on trimming results. From the combination of corrected/uncorrected reads and trimming software used, four read sets (reads processed by Rcorrector and Trimmomatic, Rcorrector and Skewer, Trimmomatic only, and Skewer only) for each species were used in downstream analyses.

Analysis

Generation of *de novo* transcriptome assemblies

A series of *de novo* assemblies were carried out with Trinity, SOAPdenovo-Trans and Trans-ABySS software packages (Table S1). For each species, assemblies of a single control library, a single treatment library or a combination of both libraries were performed, using each of the four preprocessing techniques as input (Skewer corrected, Skewer uncorrected, Trimmomatic corrected, Trimmomatic uncorrected), to yield a total of 24 initial runs from each assembler (Figures 1 and S1). For the assembly of two individual libraries, the results were combined post-assembly (Figures 1 and S1). The possible benefit of this approach is the reconstruction of specific transcripts from control and treated samples without mixture of alternative splice variants, at the expense of including a smaller data input size that may induce fragmentation of assemblies as well as a requirement to merge the separate assemblies afterward. This approach is contrasted to the second method, which combines multiple samples in a single assembly run; this approach aims at reconstructing longer and more complete transcripts despite mixing fragments from splice variants.

Trinity, SOAPdenovo-Trans and Trans-ABySS responded differently to number of input reads and how they are pre-processing (Figure 2). Trinity and Trans-ABySS produced transcriptomes with similar number of transcripts, generally increasing with the number of input reads, and with similar N50 scores. By contrast, SOAPdenovo-Trans produced transcriptomes with 27-52% fewer transcripts (80000-290000 sequences). SOAPdenovo-Trans also demonstrated more sensitive to the trimming and

correcting methods, with the use of Trimmomatic yielding a larger number of transcripts and increased N50 statistic. For both species, the highest observed N50 was achieved by uncorrected, Trimmomatic-trimmed reads and 4 input samples assembled with SOAPdenovo-Trans. On the contrary, Skewer-trimmed reads had reduced transcript numbers and N50. The N50 from Trinity and Trans-ABySS assemblies followed a more constant pattern, with Trinity reaching a higher N50 (440-580 bp) compared to 390-465 bp from Trans-ABySS. Trinity also yielded a higher N50 in VA than VC and a slight improvement when using 4 samples. Detonate [22], a reference-free evaluation tool, was used to compare each set of transcriptomes formed from the same set of reads, where scores closer to zero indicate better assemblies. Transcriptome quality as assessed by Detonate was highest in Trinity, closely followed by Trans-ABySS; error correction and use of Trimmomatic had a positive impact on these metrics.

Figure 2. Basic statistics of *de novo* transcriptome assemblies and CD-HIT or RapClust reduced transcriptomes.

Individual assemblies are plotted with the number of input fragments along the x axis. Lines are drawn to visually associate assemblies from the same species, assembler (SOAPdenovo-Trans, Trans-ABySS or Trinity) and error correction strategy (with or without Rcorrector). Total number of transcripts, N50 value, percent of GC content and Detonate scores (rows) are shown for initial assemblies, assemblies clustered with CD-HIT and assemblies clustered with RapClust (columns). Point colors indicate species and number of samples used on assembly. Point shapes indicate use of error correction (cor) or not (Uc) and trimming software (Skewer or Trimmomatic).

GC content of final transcriptome assemblies also varied by assembly strategy. Our results (Figure 2) contained assemblies of 42.3-43.9% GC for VA and 42.1-43.3% GC for VC, with the highest variability across samples found with SOAPdenovo-Trans. GC content was generally higher and more variable when reads were preprocessed by Skewer, possibly indicating the role of residual primer sequences or low quality bases in lowering final GC content. When input reads were trimmed with Trimmomatic, assemblies generally had very similar GC content across assemblers. The assemblies for VC 4s with Trimmomatic had GC content between 42.1-42.2%, matching the 42.2% of

predicted VC gene models from the reference genome [33]; VA transcriptomes had 42.3-42.4% GC under the same conditions.

Quality assessment can also be measured as the proportion of RNA-Seq reads used to generate each assembly that map back to the transcriptome (Figure 3). Read support (percent reads mapped, top row) was best for Trinity, ranging from 66% to 74%, followed by Trans-ABySS with 60-70%, and was very variable in SOAPdenovo-Trans, 9-56%. Strict trimming with Trimmomatic and error correction had an overall positive impact on read support. All assemblers showed reduced read mapping with uncorrected reads and Skewer trimming; the trend was most pronounced for SOAPdenovo-Trans, with over 30% average reduction in mapping rate when using Skewer uncorrected than Trimmomatic corrected reads.

Figure 3. Read and annotation support of *de novo* transcriptome assemblies and CD-HIT or RapClust reduced transcriptomes.
Quality metrics for assemblies, including percent of input reads that map back to assemblies, the proportion of transcripts with a putative open reading frame (ORF), and completeness as determined by the presence of BUSCO orthologs (rows). These metrics are represented for initial assemblies, assemblies clustered with CD-HIT and assemblies clustered with RapClust (columns). Lines are drawn to visually associate assemblies from the same species, assembler (SOAPdenovo-Trans, Trans-ABySS or Trinity) and error correction strategy (with or without Rcorrector). Point colors indicate species and number of samples used for assembly; point shapes indicate use (cor) or not (Uc) of error correction and trimming software (Skewer or Trimmomatic).

In addition to assembly metrics, functional annotation of transcripts was done to assess putative biological information contained in the transcriptomes. An initial observation of putative coding regions consisted of finding complete open reading frames (ORFs) with at least 50 amino acids from start to stop codon. SOAPdenovo-Trans showed strong variations by trimming software, with Skewer transcriptomes having 7-12% of transcripts with predicted ORF versus 25-31% with Trimmomatic (Figure 3). Trinity, between 12-17%, had 2-5% higher content on ORFs than Trans-ABySS, which ranged 8-15%. Finally, assemblers were compared as function of completeness of their

assemblies, indicated by the total number of conserved orthologs (BUSCOs) present in the transcriptomes, from a total of 1440 plant BUSCOs. Trans-ABySS yielded the assemblies with highest completeness, with 792-1217 identified BUSCOs, closely followed by Trinity with an average of 40 fewer BUSCOs per transcriptome. SOAPdenovo-Trans again showed strong variation with trimming type, yielding between 237-566 BUSCOs with Skewer and 645-951 with Trimmomatic.

Overall, these results show the impacts error correction, trimming, and assembly software can have on transcriptome assembly results. Error correction contributed to transcriptomes with more transcripts, with higher completeness, and with decreased GC content; for Trinity and Trans-ABySS, error correction promoted higher N50 and ORF content while decreasing percent of reads mapping back to transcriptomes. These results are in agreement with previous reports showing improvement of assembly quality after using an error correction tool [8, 38]. Use of strict trimming, such as with Trimmomatic, generally improved transcriptome metrics and all Detonate scores, with a smaller number of total transcripts, improved N50, more consistent GC content, better rate of mapping of reads, and higher proportion of coding regions, with very little loss of completeness when using 4 samples. Use of Skewer-trimmed reads had a particularly negative effect on SOAPdenovo-Trans, including reduced number of transcripts, reduced N50, reduced Detonate score, lower percent of reads mapping, much lower number of identified ORF, and lower completeness. VA transcriptomes differed from those of VC with a generally lower number of transcripts and higher Detonate scores. Using on the Trans-Abyss and Trinity assemblies, more differences in VA versus VC can be observed, including slightly higher N50 and identified ORFs in VA assemblies, but more completeness in VC assemblies. Using 2 samples yielded fewer transcripts and a lower percent of reads mapped and lower completeness than those from 4 samples, despite their higher Detonate scores.

Clustering of *de novo* assemblies

Assemblies may contain sequences from highly similar gene isoforms, transcript isoforms of a same gene and, in the case of polyploids, homoeologous genes, that may be considered redundant and lead to reads mapping to multiple locations. In addition,

considering that plants contain 37000 proteins on average [39], the number of transcripts from all of the *Vaccinium* assemblies (Figure 2) largely surpasses this quantity. Tools aimed at the reduction of such redundancy are widely used to select non-redundant representative sequences [15, 40, 41]. We have compared the clustering capabilities from two tools with very different approaches (Table S1). CD-HIT was used to select long representative transcripts and remove smaller redundant sequences at 95% similarity cutoff. RapClust groups transcripts based on the information of multi-mapped reads, and removes transcripts with low read support. CD-HIT returns a classification of transcripts into clusters and a set of representative transcripts with reduced redundancy, while RapClust returns clustering information suited to be used for downstream differential expression analysis but does not report a reduced transcript set. For the sake of comparing results, the longest transcript from each cluster generated by RapClust was selected to form corresponding reduced assemblies. Prior to clustering, single-sample assemblies were combined into a merged assembly, with expected introduction of high redundancy. Then, transcripts from the 16 assemblies (8 per species) and three assemblers (Figures 1 and S1) were subjected to classification into clusters with either of these tools.

Clustering had a noticeable impact on assemblies (Figure 2), with RapClust producing fewer clusters in comparison to CD-HIT’s reduced transcript set in all cases. Noticeably after application of RapClust, Trinity and Trans-ABySS assemblies had a very similar number of transcripts, N50, and Detonate scores. On average, the number of clusters after CD-HIT and RapClust were 22% and 51% smaller than the initial number of transcripts, respectively, for both Trinity and Trans-ABySS, and 5% and 26% after SOAPdenovo-Trans. To a lesser extent, the degree of clustering varied by type of assembly and species. Despite the 4s assemblies having larger initial numbers of transcripts, the percent of removed or clustered transcripts was greater in 2s than 4s assemblies. Thus, after clustering a larger proportion of representative sequences was retained on 4s assemblies compared to 2s assemblies by 12%, 13% and 8.7% by CD-HIT, or 2.5%, 3.3% and 15% by RapClust, on Trinity, Trans-ABySS and SOAPdenovo-Trans, respectively. Clustering only showed small difference by species with Trinity assemblies, with 3.2% more sequences retained as clusters in VA than VC. These trends are likely due to the putative higher redundancy in 2s assemblies and the presence of

homoeolog genes due to polyploidy in VC. Clustering has a variety of impacts on N50. The N50 of Trinity assemblies was not much changed while the N50 for Trans-ABySS assemblies was increased. For SOAPdenovo-Trans, the N50 was reduced after clustering, particularly with Trimmomatic trimming, from the highest N50 of 1260 to 1180 and 1030 after CD-HIT and RapClust, respectively. Detonate scores were used to evaluate the original assembled transcripts with the three assemblers as well as the cluster representative sequences yielded by CD-HIT and the longest transcript from each RapClust cluster. Clustering with CD-HIT did not substantially modify Detonate scores, while for RapClust, Trinity scores were slightly lowered.

GC content of clustered assemblies (Figure 2) was reduced by an average 0.2% from the original assemblies in those from 2 samples and generated with Trinity or Trans-ABySS. The same reduction was observed in 2s assemblies when using RapClust on SOAPdenovo-Trans assemblies. In all cases, values were reduced closer to the putative GC percent found in the diploid VC reference genes. All changes were minor, with most assemblies from 4 samples and Trimmomatic-trimmed reads staying close to their original values after clustering. Clustering yielded a less than 5% decrease in support from RNA-Seq reads of the transcriptomes generated with Trans-ABySS and SOAPdenovo-Trans (Figure 3) or clustered with CD-HIT. Trinity assemblies had an average of 7% loss of read support under clustering with RapClust, close to Trans-ABySS values but still having the highest support. Differences in ORF content between Trinity and Trans-ABySS decreased with clustering as Trans-ABySS modified ORF content from 8-15% to 8-12% after CD-HIT and 12-15% after RapClust, while Trinity changed from 12-17% to 11-15% and 13-15% after CD-HIT and RapClust, respectively. Lower values of SOAPdenovo-Trans remained at 7% after clustering, but the highest ORF content, originally at 31%, changed to 32% and 27% after CD-HIT and RapClust, respectively. The variation of the proportion of transcripts containing a coding sequence was not mirrored by the degree of completeness. Clustering with CD-HIT did not modify the overall completeness of assemblies, while RapClust slightly decreased them by 14, 43 and 24 in Trans-ABySS, Trinity and SOAPdenovo-Trans, respectively.

Clustering with CD-HIT was effective to reduce the redundancy of transcriptome assemblies in Trinity and Trans-ABySS, without substantial modification of quality

metrics. This reduction affected especially 2s assemblies compared to 4s, concomitant with the expected higher artificial redundancy induced in 2s assemblies after the merging of single assemblies. SOAPdenovo-Trans assemblies displayed little modification from CD-HIT clustering, suggesting a lower number of isoforms or less fragmentation in the output transcriptomes. By contrast, RapClust reduced the number of transcripts from all three assemblers, with different effects. SOAPdenovo-Trans assemblies had a lower N50 and ORF content, but similar read support, Detonate scores and completeness after RapClust clustering and selection of the longest transcript as a representative. For Trans-ABySS assemblies, there was similar read support, Detonate scores and completeness after RapClust, but higher N50 and ORF content suggests a reduction of smaller and non-coding transcripts. For Trinity assemblies, the similar N50 and ORF content, but lower read support, Detonate scores and completeness suggests a reduction of transcripts of all sizes by RapClust.

Biological consistency of clustering methods

The general evaluation of *de novo* transcriptome assemblers revealed that Trinity assemblies have balanced metrics across options, with high support of RNA-Seq reads, medium N50 and proportion of coding transcripts, and high completeness. Trans-ABySS was competitive on completeness and balanced on GC content, but had lower read support, N50 and ORFs. SOAPdenovo-Trans was very sensitive to the input read trimming, showing good metrics with Trimmomatic, but had an overall low read support and completeness compared with the other methods. Thus, from here on, Trinity assemblies are selected to explore in more detail assembly metrics and mapping of RNA-Seq reads.

To further explore the effect of clustering, we utilized the published reference genome from the diploid *Vaccinium corymbosum* [33]. We presented two scenarios, one with a distant diploid species and other with the same species but different ploidy level. To explore the portion of transcripts with sequence homology that each species shares with the reference genome, we mapped the clustered transcriptomes to it. Transcripts were classified as uniquely mapping, mapping to multiple loci, translocated (parts of the transcripts were mapped to different locations on the genome) or not mapping. These

results were combined with coding sequence (cds) predictions from Transdecoder and blast homology results. Overall, transcripts generated for the diploid VA mapped to the reference genome at a larger proportion than the tetraploid VC, and the 2-sample merged assemblies (2s) mapped at a higher rate than the 4-sample ones (4s) (Figure 4). Specifically, average mapping rate of transcripts was 66% and 57% in VA 2s and 4s, and 57 and 43% in VC 2s and 4s. Thus, the use of multiple samples leads to a higher proportion of transcripts not resembling the genome, representing species-specific transcripts and possibly artifacts. While VA has higher mapping rates than VC, discrimination between a true higher similarity or an effect due to the read input cannot be made. The proportion of multiple mapping and translocated transcripts had little variation across transcriptomes in both species, being 5-7% and 4% respectively. Multi-mapping rate reflects highly similar regions of the genome, and translocations could indicate either true genome rearrangements or assembly artifacts such as transcript fusions (chimeras). Clustering with CD-HIT or RapClust (using a single representative sequence for each cluster), despite affecting the total number of transcripts, maintained similar proportion of transcripts in each mapping category; on average, RapClust increased 2.2% unique and decreased by 0.5% multiple and translocated mapping transcripts compared to CD-HIT. Trimming also influenced mapping; assemblies from reads trimmed with Trimmomatic showed an average 2% higher unique mapping rate than their counterparts with Skewer, suggesting better accuracy with stricter trimming. No effect was observed from error correction.

Figure 4. Mapping of *de novo* assembly transcriptomes to *Vaccinium corymbosum* reference genome and annotation of transcripts. Transcripts mapped either uniquely to the genome (uniq), to multiple locations (mult), with translocations (transloc) or did not map (out). Annotation from prediction of coding sequences (cds) using homology results from blast is divided as "No Functional Annotation" (map), "CDS Only" (cds) and "CDS with Blast Hit" (blast). Transcriptomes for *V. arboreum* (VA) or *V. corymbosum* (VC) produced from two (2s) or four (4s) samples were clustered with either CD-HIT (C) or RapClust (R). The last two letters indicate trimming with Trimmomatic (T) or Skewer (S), and use (C) or not (U) of error correction of RNA-Seq reads.

Prediction of a coding sequence and the extent to which they may be coding for proteins was used as an indicator of biological information contained in transcripts. Transdecoder finds all ORFs and selects the most likely putative cds using homology search results from blast. 51-59% of transcripts contained a predicted cds for all assemblies (Table S3). Compared to the length of original transcripts, the average length of cds decreased by 13% and 20% on 2s and 4s assemblies, respectively. Transcripts within each category (unique, multiple, translocated and not mapping) had different likelihoods of having a predicted coding sequence and additionally of cds showing homology to known proteins. On average, 49.2%, 51.8%, 54.8% and 64.5% of the transcripts in the categories unique, multiple, translocated and not mapping, contained a predicted coding sequence (Figure 4, Table S3). In addition, 54.0%, 42.4%, 55.2% and 20.1% of the cds on those categories, respectively, had a blast hit. Thus, a relatively large proportion of cds do not map to the genome, particularly in VC with 4 samples (72%). These transcripts also show low similarity to known proteins, leaving unclear whether they belong to true novel transcripts or they are assembly artifacts. For transcripts that mapped to the genome, VA exhibited greater proportion of annotation than VC. Nonetheless, comparing absolute number of transcripts, VC has a larger set of mapping transcripts with cds but also an even larger number of transcripts not matching the reference than VA. Influence from the other analysis options on annotation distribution were less drastic. Clustering with RapClust had a positive effect on the proportion of cds and blast results of unique and translocated transcripts, especially in 2s assemblies, in the range of 0.5-5.5%. Changes due to read trimming or correction were lower than 2%.

Specific variations on Trinity transcriptome completeness throughout the sequential stages of processing (i.e. assembly, clustering and cds prediction), used the BUSCO tool to report, for each of the 1440 near-universal conserved orthologs searched, whether it is present in the assembly as complete and single-copy, complete and duplicated, fragmented, or missing. Examining the impact on BUSCO results by read processing, assemblies from soft trimmed reads with Skewer presented higher completeness (Figure 5A). Interestingly, error correction improved the formation of complete BUSCOs on 2s assemblies, while it did not have a significant effect on 4s assemblies. However, the major options influencing completeness were blueberry species and number of samples

used. Thus, assembly of complete genes was improved in VC compared to VA, and in assemblies of four rather than two samples (Figure 5A). Overall, completeness of CD-HIT clusters was very similar to those of *de novo* assemblies, while RapClust clusters contained fewer total BUSCOs. Selection of cds further decreased completeness, either decreasing complete genes or also increasing fragmented genes, mostly in 4s assemblies. In addition, the distribution of complete vs fragmented BUSCOs shows a trend where a reduction in total BUSCOs is followed by an increase in fragmented BUSCOs (Figure 5A). Following this trend, the rate of fragmented BUSCOs was not significantly modified by read processing nor by clustering with CD-HIT, while RapClust increased it except in VA 2s, where fragmented BUSCOs were reduced.

Figure 5. Evaluation of assembly and clustering methods for Trinity transcriptomes. (A, B) Completeness assessment with BUSCO tool subdivided into complete versus fragmented BUSCOs (A) or single-copy versus duplicated complete BUSCOs (B). Dotted lines represent isolines of BUSCO numbers from a total search space of 1440 orthologs. Dot colors indicate assembly stage and areas assembly type. Stages of the assembly are divided into initial *de novo* assembly (asmb), clustered with either CD-HIT or RapClust, or predicted coding regions (cds). Assembly type indicates the combination of blueberry species (V. *arboreum*, VA; *V. corymbosum*, VC) and the use of two independent assemblies merged (2s) or assembly of four samples (4s). Shapes represent read pre-processing options, with (cor) or without (Uc) error correction, and the use of Skewer or Trimmomatic (trimm) trimming tools. (C) Distribution of mean Jaccard scores on CD-HIT and RapClust clusters of transcriptome assemblies. Scores range between ~0 (low clustering of co-annotated transcripts) and 1 (perfect clustering of co-annotated transcripts). (D) Distribution of genome versus assembly base coverage on multiple *de novo* assemblies mapped to *Vaccinium corymbosum* reference genome after redundancy reduction with either CD-HIT (larger points) or RapClust (smaller points). Shapes indicate read processing, with (cor) or without (Uc) error correction, and trimmed with either Trimmomatic (trimm) or Skewer.

While some gene families may have undergone expansion or contraction since the *Vaccinium* common ancestor, we expect the majority of transcripts to provide one-to-

one orthologs for the VA gene set and two-to-one orthologs for the tetraploid VC gene set. Coincident with their ploidy, duplicated vs single-copy ratio in unclustered VA *de novo* assemblies was half that of VC (0.50 in 2s and 0.58 in 4s). Also, the duplication ratio in 2s vs 4s unclustered assemblies was 1.25 in VA and 1.45 in VC, supporting higher redundancy in 2s assemblies. These ratios are independent from the size of transcriptomes. Clustering was efficient to remove redundant genes, as shown by the reduction of duplicates. RapClust drastically removed most duplicated BUSCOs, leaving 20-30 duplicated BUSCOs for all assemblies, while CD-HIT performed a reduction proportional to the assembly length of 62% on 2s and 44% on 4s assemblies. While the clustering did remove many duplicated BUSCOs, most became single copy BUSCOs and were not lost from the assembly altogether. Only in the 4s assemblies, comparing the original assembly to RapClust cluster transcripts, there was a significant decrease in the number of complete BUSCOs (Figure 5B). Ideally, clustering would reduce splice isoforms and partially assembled transcripts, however the reduction in completeness suggests possible removal of gene isoforms in both species, and collapse of homoeologs in the tetraploid VC, especially by RapClust.

BUSCO results were not only used to assess completeness, but also to measure the success of the clustering methods using an adaptation of the Jaccard similarity method. Taking advantage of BUSCO consensus sequences, transcript co-annotation was calculated as the number of transcripts with the same BUSCO annotation within a cluster (set intersection) divided by the total number of transcripts with that BUSCO annotation or in the cluster (set union). The result is a value in the range 0 to 1, from low to perfect shared annotation of transcripts within a cluster. This method not only indicates the degree of co-annotation depicted by each clustering algorithm but also compares the putative biological relevance of clusters. On this respect, RapClust consistently outperforms CD-HIT on clustering of co-annotated BUSCO genes (Figure 5C). Clusters from the diploid VA were markedly better co-annotated from those of VC. Generally, RapClust performance was enhanced on larger transcriptomes, while CD-HIT performed better on smaller ones. In relation to read processing, Trimmomatic and uncorrected reads generally achieved higher scores.

To explore the percent of the blueberry genome captured by the *de novo* assemblies, base coverage was calculated for transcripts that mapped uniquely to the diploid reference genome (Figures 4 and 5D). Assembly base coverage is the proportion of bases of each transcript assembly that were mapped to the reference genome, and genome base coverage is the proportion of the reference genome covered by the transcripts. In general, both metrics showed inverse correlation. Thus, genome coverage was enhanced with the use of Skewer, four samples and CD-HIT, while decreasing assembly coverage. Thus, genome coverage is concordantly improved by those options that also increase transcriptome size, where a larger number of transcripts is able to better represent genomic sequences. This is true for both blueberry species, with the distinction that VC exhibits both better genome and assembly coverage than VA, consistent with phylogenetic proximity to the reference genome species. On the other hand, trimming with Trimmomatic, two-sample assemblies and clustering with RapClust had better assembly coverage, but lower genome coverage. This suggests that transcripts generated from more restrictive options are more likely to be real genes that can be found in the genome, but the more restrictive options do exclude some genes. Error correction did not follow this trend, and generally decreased assembly coverage while not affecting genome coverage.

Read mapping to reference genome

As an alternative to *de novo* assembly, RNA-Seq analysis for these two species could utilize a mapping approach with the publicly available genome of diploid VC. With this approach, an entirely different set of software options become available. In this case, mapping to a genomic reference that is evolutionarily diverged from the sequenced species may make accurate read mapping more difficult. For the diploid VA, mapping to homolog genes is expected, while for the tetraploid VC, reference genes may be mapped by reads originating from both homolog and homoeolog sequences. To account for sequence divergence, we compared results from five representative mapping software programs, run with either default settings or increasing mismatch tolerance (Figure 6A, Table S1). Overall, aligners behave similarly on both blueberry species. The programs that yield the most mapped reads are Stampy and GSNAP, both of which were designed to tolerate more sequence divergence during mapping, although only Stampy surpassed 5% mismatch rate (Figure 6B). Bowtie2 and HISAT2 yielded the

lowest mapping rates. The addition of relaxed conditions, despite modifying the percent of mismatches tolerated on alignments, did not have a significant effect on mapping results of GSNAP, Stampy and STAR; it lowered the mapping rate for Bowtie2 and increased for HISAT2, especially in VA. The effect of trimming was correlated with the number of available reads to be mapped; thus, Skewer improved mapping rates by 5-11% compared to Trimmomatic (Table S4). Finally, corrected reads, though not significant, promoted an increase in mapping rate for all options, with 0.7 and 0.5% average increase in VA and VC, and up to 2.5% in HISAT2 in VA.

It is desirable to utilize the maximum number of reads as possible in differential gene expression analysis, as increased depth of read counts leads to more sensitivity in statistical analysis. For example, more depth would increasingly allow detection of differences in lowly expressed genes or genes with small log fold changes in expression between treatments. To use this as a quality metric, we examined the successful conversion of raw reads to countable reads for each gene model using the software HTSeq. Starting from all mapping results, a read may not be converted to a countable read due to low quality mapping, multiple alignments or mapping to a genomic region without an annotation. The influence of each factor varies by mapping tool (Figure S2). The main cause of failed read conversion into counts was low quality of read alignment, found in Bowtie2, HISAT2, Stampy and GSNAP, by decreasing magnitude. The second major factor that prevented counting was mapping within an intergenic region, which accounted for 5-13% of mapped reads (Figures S2 and S3). Mapping to exonic features showed even larger variability, ranging from 57% displayed by Stampy, to 80% by HISAT2, varying by mapping tool (Figure S3). In relation with mapping rate, these values indicate that both programs have similar mapping rates to exons but Stampy is mapping more reads to non-exonic regions that may present higher sequence divergence. After collecting useful read counts, count rates to gene models were smaller than mapping rates by 14.2%, 10.9%, 7.5%, 15.7% and 3.3% for Bowtie2, GSNAP, HISAT2, Stampy and STAR, representing a loss up to 45% of mapped reads for Bowtie2 and below 15% for STAR (Figure 6A, right panels). Globally, modification of mismatch tolerance increased this loss in Bowtie2 and Stampy, and reduced it in HISAT2. Read loss using Skewer compared to Trimmomatic was larger on GSNAP and Stampy, and smaller on HISAT2 and Bowtie2. Interestingly, the rate of mapped reads

not turned into counts in STAR was constant under the pre-processing and software options tested. After counting, count rates (Figure 6A, lower values) displayed similar response to read processing as mapping rates discussed above, with GSNAP and Stampy showing equally high count rates.

Figure 6. Read mapping to *V. corymbosum* reference genome. (A, left panels) Proportion of total reads mapping to reference (grey boxes or higher values), converted to counts (white boxes or lower values) and (A, right panels) percentage of the difference, and (B) mismatch rate depicted by each software option. Five mapping software programs were compared at default and modified settings to increase mismatch tolerance. Reads used (cor) or not (Uc) error correction, and Trimmomatic (trimm) or Skewer trimming software. Results are distribution of 8 samples.

An important issue in science is reproducibility of results, that in the case of mapping results can be reflected as similarity of gene count profiles, which ultimately determine genes that are differentially expressed. Correlation of counts was calculated across all blueberry samples comparing the 20 combinations of read processing and mapping software with default options (Figure 7). Concomitant with their similarity on mapping results to the reference genome, VA and VC shared major correlation patterns between software programs, where two major groups are formed. This grouping is consistent with the alogrithmic similarities of the software, i.e. one group is composed by Bowtie2 and HISAT2, which utilize a FM-index, and the second group includes GSNAP, Stampy and STAR, which use a combination of suffix array / hash table. Correlation was usually influenced by the trimming option, so that Skewer significantly improved correlation on GSNAP and STAR, Trimmomatic on Bowtie2 and Stampy, and HISAT2 was ligthly affected by trimming. Interestingly, only Bowtie2 and HISAT2 responded to read correction, suggesting higher sensitivity to errors by the FM-index.

Figure 7. Correlation of gene count profiles after mapping to *Vaccinium corymbosum* genome. Values are mean of 8 samples in either *V. arboreum* (VA, upper triangle) or V. *corymbousm* (VC, lower triangle). Each row/column corresponds to a unique combination of mapping software, trimming software and error correction.

Read mapping to *de novo* assemblies

The previous section focused on the effects of read correction, trimming and alignment software on read mapping to a reference genome. Here, a similar analysis is performed though using *de novo* assemblies from Trinity clustered with CD-HIT. To simplify the analysis, reads that underwent certain correction and trimming processing (e.g. samples with corrected reads trimmed with Skewer), were only mapped to the assemblies produced by reads with the same pre-processing. This method of *de novo* assembly then alignment is common for RNA-Seq analysis when no reference genome is available, and has advantages, including that mapping to transcript assemblies is usually contiguous, instead of spliced, and that assemblies are species specific, unlike a distant reference genome. All the aligners previously used for the genome alignment may also be used with transcriptomes. In addition, we incorporated the Salmon tool for transcript quantification, which is built solely for alignment of reads to a transcriptome.

Using *de novo* assemblies as the reference, mapping performance of the five aligners showed lower variability by condition (trimming and type of assembly) compared to mapping to the genome, with Stampy and GSNAP again as best performers (Figure 8). The mapping profile was similar for both species, with higher mapping rates for VC than VA by 1.4% using Skewer and 2.5% using Trimmomatic, except for Salmon. Also, 4s assemblies had consistently better mapping rates than 2s, with improvements for Skewer/Trimmomatic of 3.7/3.0% in VA and 3.8/3.4% in VC. Examining only the effect of trimming, yield is likewise correlated with the number of reads available for mapping, so that Skewer had on average 12.5% more reads mapped than Trimmomatic. Finally, error correction of reads did not have a significant effect on read mapping. Examining conversion of raw reads to countable reads, 30-45% and 22-30% of mapped reads in 2s and 4s assemblies were not able to be turned into counts, with higher values on 2s assemblies than 4s ones (Figure 8, right panels). For Bowtie2 and Stampy, the major cause of read loss was low quality alignments, while for GSNAP, HISAT2 and STAR most of the dropped reads were multi-mapped (Figure S4). Read counts further reduced variability across programs, and intensified the difference between mapping to 4s compared to 2s assemblies, increasing by 9.1/6.1% in VA and 9.8/7.9% in VC for Skewer/Trimmomatic, respectively. The difference between using Skewer or

Trimmomatic was reduced to an average of 9%. The different results yielded by Salmon reflects its different algorithm, which performs pseudo-mapping to estimate abundance, but does not report mapping results in a format suitable to do quality assessment of alignments. The consequence is that Salmon has an artificially higher estimated count rate than reads mapped, and since no reads are filtered out for quality score, Salmon has higher count rates than other approaches.

Figure 8. Read mapping to CD-HIT clustered *de novo* assemblies. Proportion of total mapped reads (left panels, grey boxes), converted to counts (left panels, white boxes) and percentage of the difference (right panels). Six mapping software programs were compared at default settings on assemblies made from four samples, produced either by two sets of 2 samples independently assembled (2s) and later merged or from the four samples assembled together (4s). Reads used (cor) or not (Uc) error correction, and Trimmomatic (trimm) or Skewer trimming software.

In the case of mapping to a *de novo* assembly, to calculate a correlation of mapping results is not directly due to each assembly having their own set of transcripts. Hence, rather than program-to-program correlation, which is showed on the previous section, reference-to-assembly count profiles were compared (Figure 9). To do so, the reference gene model gene space was used for such comparison. New count profiles for assembly mapping results were obtained from adding counts of all transcripts mapped to each single reference gene model. Then, they were compared to results with the reference genome by same read pre-processing and mapping software. Utilization of the reference genome from diploid VC, though useful for a shared gene set to compare, has the inconvenience of not representing species-specific transcripts (blue bars in Figure 4). VA is a sister species but is also a diploid, so one-to-one homology may be expected. However, tetraploid VC assemblies not only contain a larger proportion of transcripts that do not match the genome, but also splice isoforms and lowly-diverged homoeolog sequences are expected to map to same gene models. Likewise, balancing this effect, reads originated from transcripts sharing sequence similarity are expected to map to the same gene model on the reference genome.

The highest assembly-to-genome correlation values are obtained on the diploid VA, which reach 75% on all programs (Figure 9). However, the best performing program differs by species: GSNAP and Stampy for VA, and Bowtie2 and HISAT2 for VC. For both species, results with the larger 4s assemblies are better correlated to the genome than the 2s assemblies. Overall, the preference for trimming software, if any, is opposite by species; Skewer and Trimmomatic improves 2s and 4s assemblies on VA, respectively, and Skewer improves 4s assemblies in VC. These differences caused by read processing are more prominent on 4s assemblies, while on 2s assemblies they induce significant changes on VA with Bowtie2, HISAT2 and STAR. This suggests that stricter trimming in the distant VA may help mapping accuracy on the diploid VC genome, especially with Bowtie2 and HISAT2 4s, while gentle trimming in the tetraploid VC may help by either better assembly of transcripts or read mapping. Salmon results correlate well with the different aligners in VA, especially GSNAP and Stampy (Figure 9, bar colors), while the tetraploid VC has overall poorly-comparable results. This suggests that Salmon transcript quantification may be better suited for less complex genomes.

Figure 9. Correlation of gene count profiles obtained with *de novo* assemblies and the reference genome. Counts of transcripts aligned to a same reference gene model were added and re-annotated as that gene model. Correlation was calculated on the common set of gene models with non-zero counts on both reference and assemblies, by mapping software and read pre-processing (error correction and trimming). Uc stands for uncorrected, cor for corrected, trimm for Trimmomatic. Color indicates mean correlation of reference counts with Salmon, a transcript-specific quantification tool. Values are mean ± sd of 8 samples.

Discussion

RNA-Seq is an affordable and versatile tool to analyze transcriptomes of any species. Depending on the available resources, it can be guided by a reference genome or by building custom assemblies that will reflect the transcripts present in the samples.

However, many confounders make the analysis less straight-forward than simply trimming adapters, assembling reads as needed and mapping to a reference. Some of these confounders are common for any RNA-Seq data analysis, such as sequencing errors, repetitive sequences, natural heterozygosity and variants, while the analysis of a species other than the reference has additional sequence variation and, in the case of a polyploid, gene redundancy. Thus, we explored the repercussions of various informatic choices on the final gene expression profiles.

Illumina short read sequencing, though very accurate, is not exempt of sequencing errors. One strategy to deal with low quality nucleotides aims to correct reads, usually by replacing poorly represented *k*-mers with similar ones of higher frequency patterns [38]. Effectivity of error correction on RNA-Seq data is lower than on genomic data due to differences in expression level and splicing and is less dependent on the organism of study [8]. Despite sequencing errors of Illumina technology occurring at a reported average rate of only 0.1% bases [6], Rcorrector modified 0.7% bases in both species. While error correction tools can reduce sequencing errors, they can also introduce new errors at a variable rate, especially for complex datasets [38]. For a complex gene family or when examining a polyploid, this could be a significant problem with some reads converted to the sequence of a close homolog, leading to incorrect mapping and/or misassembly. However, in this study read correction did not reflect significant variation in overall mapping success. It induced a small amount of variation only on those aligners that use an FM-index, Bowtie2 and HISAT2, and thus require perfect matching for seeding an alignment. Read correction was more important for assemblies, which exhibited larger changes depending on correction state, such as larger number of transcripts, higher Detonate scores or higher completeness when using corrected reads in most cases, especially with SOAPdenovo-Trans. Previous research also demonstrated that error correction impacts genome assembly [38].

Trimming is required to, at the least, remove sequencing adapters, and often also addresses short reads and low quality bases. The broadly-used tool Trimmomatic implements strict trimming based on sequencing base quality, where trimming removes low quality bases that could lead to complex or incorrect de Bruijn graphs, but also reduces read length, which may have a negative impact on coverage bias [38]. Skewer

takes a much less stringent trimming approach. The extent to which trimming of low quality bases is beneficial for downstream analyses was explored for DNA-Seq [42], suggesting a positive effect on genome assembly despite increased fragmentation, and a tradeoff between accuracy and recall of assemblies. In our experiments, similar effects derived from trimming were shown on both the diploid or tetraploid species, especially with Trans-ABySS or Trinity. We found that Skewer (soft trimming) usually led to more complete assemblies at the expense of a larger amount of non-coding transcripts, while Trimmomatic (i.e. strict quality trimming) improved support from input reads and consistency of GC content across assemblers; in Trinity clusters, Trimmomatic also reduced fragmentation of assemblies and enhanced biological consistency of clustering. In mapping experiments, higher quality reads are mapped at a larger relative proportion, however, this is at the expense of losing many reads at the trimming stage, many of which may have been successfully mapped downstream. Nonetheless, both options can lead to comparable expression profiles, mostly if mapping tools can deal with bases of lower quality [42].

There are cases where transcriptome assemblies are required, such as absence of a suitable reference genome, or discovery of novel isoforms. For transcriptome assembly with samples derived from various conditions, two approaches are common; one in which the samples are pooled into a single run [40, 41] and one in which samples are assembled independently [43-45]. The major interest is to obtain transcripts that are specific to each sample, and combination of reads is a potential source for mis-assembly or formation of chimeras. In this respect, we found that transcripts from separate samples had significantly higher assembly base coverage (transcript bases mapped to the reference genome), although the combined samples had better genome base coverage (reference genome bases covered by transcripts). However, merging individual assemblies generates high redundancy. This effect was studied in wheat, reporting that redundant merged assemblies showed improved read mappability with Trinity but lower with Trans-ABySS, but also had less continuity than assemblies from pooled samples, and their quality decreased after clustering [43]. We found improved read support on merged assemblies for the three assemblers, but lower mean transcript size and completeness. A strong reverse correlation between fragmentation of genes and assembled reads was also found, supporting that sequencing depth is beneficial to the

recovery of full-length transcripts [13, 15, 20]. General conclusions apply to both the diploid and the tetraploid species, although the polyploid had proportional increased duplication rate and exhibited a larger species-specific proportion of transcripts. On the other hand, proper clustering in polyploids is difficult, not unexpectedly, as it must handle isoforms of genes as well as homoeologs. This is reflected by the outcomes of the clustering methods utilized, where aggressive reduction of redundancy also leads to loss of completeness, though to a lesser extent than sequencing depth.

Scientists examining organisms without a specific reference face the decision of whether to use the reference genome of a close organism or to build a custom *de novo* assembly. Mapping to a distant reference has disadvantages, including sequence divergence at the nucleotide level, and also larger structural divergence, where genes may be missing or duplicated between the species. From our species studied, it would be expected for the distant diploid VA to have undergone greater sequence divergence than the tetraploid relative of the reference diploid VC, in which divergence would be driven by diversifying subgenomes. Mapping results to the reference genome reflect this issue, where mapping tools that have greater sensitivity to align divergent sequences, such as Stampy, GSNAP and STAR, improve mapping results of VA compared to VC, while HISAT2 and Bowtie2, which require an exact match to seed, perform better in VC than VA. Regardless of the species, we found GSNAP and Stampy to yield the highest performances on the reference genome, probably due to their ability to align divergent sequences even at default settings. On the second mapping strategy, utilizing specific assemblies allowed much higher mapping rates compared to the reference, concordant with the high proportion of transcripts not represented on the genome that are now available to be mapped. Both species displayed comparable results when mapping to an assembly, slightly better on the tetraploid VC than on the diploid VA except with Salmon, probably due to the better completeness of the VC transcriptomes. In addition of higher mapping rates, specific biological information may be present on transcripts not represented in the genome, from which 64.5% had a predicted cds, gaining insight in the processes under study. Nonetheless, besides the divergence with the reference genome, using assemblies can give similar results at 75% correlation; awareness of mismatches also played here a role, improving correlations of VA with GSNAP and Stampy, and of VC with HISAT2.

In conclusion, using a reference genome with either a distant diploid species or a polyploid relative can give reliable results, simplifying the RNA-Seq analysis by skipping *de novo* assembly and associated steps. In the present work, we expanded many possibilities from read processing to gene counting, providing a complete overview on how each of the tested options impacts gene expression profiles. On both species studied, the pipeline that yielded high outcome with comparable results using either a reference genome or a transcriptome assembly used trimming with Skewer, a combination of multiple samples for improved assembly quality, and Stampy or GSNAP for short-read mapping. This pipeline was oriented to maximize the recovery of information from RNA-Seq reads, working with the specific case where samples and reference genome are not from the same organism. While we suggest that this strategy can be extrapolated to other systems, our study also highlights the many downstream impacts software analysis decisions can have on results. For scientists faced with complex RNA-Seq analysis projects, testing of different software packages to examine and optimize results can be beneficial.

Methods

The following methods include a brief summary of the tools that were used in this work. For detailed descriptions of the algorithms, original publications or websites are referred.

Sequencing of RNA-Seq reads of blueberry roots

Preparation of RNA-Seq libraries from root tissue of diploid *Vaccinium arboreum* cultivar FL148 and tetraploid *V. corymbosum* ‘Emerald’ blueberry species are previously described [25] and available in NCBI as bioproject PRJNA353989. Briefly, eight plants per species were acclimated to growth in hydroponic systems at either pH 4.5 or pH 6.5 for 8 weeks, after which roots were collected and flash frozen. RNA was extracted and prepared for sequencing of 100 base-pair (bp) paired-end reads on a HiSeq 2000 system (Illumina, CA, USA).

Error correction and trimming of RNA-Seq reads

Rcorrector (*RNA-Seq error CORRECTOR*) [8] is a *k*mer-based error correction method that uses a De Bruijn graph to represent trusted *k*-mers, a method similar to that used on *de novo* assembly. Rcorrector v1.0.2 was applied to raw reads with default parameters. Then, sets of corrected and uncorrected reads were trimmed for removal of Illumina adapter sequences using either Trimmomatic v0.35 [36], specifying parameters ‘SLIDINGWINDOW:4:15’ and minimum read length of 30 bp, or Skewer v0.2.2 [37], with same minimum length cutoff. Trimmomatic searches adapters by finding an approximate match and aligning using a *seed and extend* approach [46], both for regular and ‘adapter read-through’ scenarios. Illumina quality scores of bases are used to determine cut points, discarding the 3’ end of the read. Skewer uses a novel *bit-masked k-difference matching* dynamic programming algorithm, which uses a variation of the *Smith-Waterman* [47] algorithm to search substrings and solve the *k-difference problem* and an extended *bit-vector algorithm* [48] to handle base-call quality values. Skewer can remove low quality bases on both 5’ and 3’ read ends, and is considerably faster than Trimmomatic. FastQC v0.11.4 [49] was used for quality assessment of reads. From each original read file (VA control, VA treatment, VC control, VC treatment), the combination of error correction and trimming generated four new sets of trimmed reads to be utilized in downstream processes: reads processed by Rcorrector and Trimmomatic, reads processed by Rcorrector and Skewer, reads processed by Trimmomatic only and reads processed by Skewer only.

de novo transcriptome assembly and redundancy reduction

Each of the four processed read sets was used for transcriptome *de novo* assembly, independently for each blueberry species, using Trinity 2.2.0 [14], Trans-ABySS v1.5.5 [16] and SOAPdenovo-Trans v1.03 [17], with *k*-mer = 25 and filtering for a minimum contig length of 200 bp. Environmental stress is expected to alter the transcripts present in the cells as well as transcript splicing patterns. To include this source of variability, two commonly used approaches were considered: (i) assemble control and treated samples independently and concatenate results after assembly, and (ii) combine two control and two treated samples in the same assembly run. Altogether, 12 Trinity assemblies for each species were generated (Figure S1). The next step consisted of

removing redundant transcripts from assemblies using either CD-HIT v4.6.6 [18] at 95% identity or RapClust [50]. CD-HIT sorts all transcripts by length and attempts to consecutively cluster smaller sequences to longer representative ones, getting classified as redundant or representative based on sequence similarity; the result included a reduced transcript set consisted of one sequence per cluster. On the other hand, RapClust was developed to group assemblies using information from multi-mapper paired-ended reads, thus requiring input from Salmon [51] aligner. From the clustering information after RapClust, reduced transcriptomes were obtained after selection of the longest transcript per cluster. This step generated 16 clustered assemblies for each species (Figure S1).

Quality assessment and functional annotation of assemblies

Transcriptome *de novo* and clustered assemblies were assessed for quality with DETONATE 1.11 [22] to calculate a score weighed with the reads used to generate each assembly, Transrate 1.0.3 [12] to get basic metrics, and BUSCO v2.0 [21] for completeness assessment. To compare the Trinity *de novo* assemblies to the genome, reduced assemblies were mapped to the diploid blueberry reference genome [35] with gmap version 2017-05-08 [52]. Base coverage was calculated on uniquely mapping transcripts using coverageBed from the BEDTools suite version 2.26 [53].

Biological consistency of clustering results was evaluated with a custom Jaccard similarity score based on the method described in [54] using the BUSCO annotation results on Trinity assemblies. Each cluster received an individual score calculated as the number of transcripts with the same BUSCO annotation within the cluster divided by the total number of transcripts with that BUSCO annotation plus the number of transcripts in the cluster that did not share that annotation. The statistic is based on amount of the intersection divided by amount of union where the two sets are (i) all the transcripts sharing a BUSCO annotation and (ii) all the transcripts in a cluster. If multiple annotations were present in a cluster, the maximum score was selected for that cluster. The result is a value between 0, indicating low co-annotation of transcripts, and 1, indicating perfect clustering of co-annotated transcripts. Clusters with a single transcript were omitted.

Putative open reading frames (ORFs) were predicted for each Trinity clustered assembly with TransDecoder v3.0.0 [55], software that incorporates results from blast [56] and Pfam [57] homology searches to select best ORF candidates. First, candidate cds encoding at least 50 amino-acid-long peptides were extracted from transcripts. Then, these were searched with blast against the plant TrEMBL protein database (evalue < 10e-5) and with HMMER 3.1b2 [58] against Pfam. Finally, a single putative ORF was selected for each transcript when possible.

Read mapping

The four sets of processed RNA-Seq reads from VA and VC were mapped to either the draft reference genome for diploid VC or Trinity *de novo* assemblies clustered with CD-HIT, using STAR 2.5.0, Stampy v1.0.28, GSNAP 2016-11-07, Bowtie2 2.2.8 and HISAT2 2.0.4. Software options were modified or not when mapping to the reference genome to increase mismatch tolerance. Salmon v0.7.2 [51], that uses quasi-mapping with a two-phase inference procedure, was specifically used on transcriptomes. Mapping metrics were collected using picard tools v2.1.0 [59] and RNA-SeQC v1.1.8 [60]. Finally, counts were obtained using HTSeq-count Version 0.6.1p1 [61].

Short read aligners can be classified by algorithmic approach as not splice-aware (Bowtie2, Stampy) or splice-aware (HISAT2, STAR, GSNAP), or by their use of an uncompressed index, such as hash table, or compressed indexes, like suffix arrays, Burrows-Wheeler transform (BWT) methods and Full-text index in Minute space (FM-index). Bowtie2 [62] uses an algorithm based on the BWT and the FM-index, which extracts seed substrings from reads, finds exact alignments with the FM index and extends with gapped dynamic algorithms like *Needleman-Wunsch* (global alignment) or *Smith-Waterman* (local alignment). Stampy [63] uses a hash table with locations of 15-mers in the genome used to search every overlapping 15-mer in the reads. Those that pass neighborhood similarity filtering are extended with *Needleman-Wunsch*. GSNAP (*Genomic Short-read Nucleotide Alignment Program*) [52] combines a set of algorithms to improve accuracy of alignment, using either hash tables or enhanced suffix arrays (ESA). Sequentially after failure of previous methods, GSNAP searches for a single continuous match, applies segment combination procedures, or employs its complete set analysis to allow for larger mismatch proportion. STAR (*Spliced Transcripts Alignment*

to a Reference) software [64] is based on an algorithm that uses "sequential maximum mappable seed search in uncompressed suffix arrays followed by seed clustering and stitching procedure". After stitching of seeds, the unmapped portions of the reads can be extended with *Needleman-Wunsch* algorithm. HISAT2 (*Hierarchical Indexing for Spliced Alignment of Transcripts*) [65] is based on the BWT and the FM-index, with operation methods adapted from Bowtie2. In addition to the global FM index, the genome is divided into a large set of small FM indexes. Read strings are first mapped to the global FM index to find candidate locations and the remaining bases are aligned with a local index, combining extension by direct comparison of sequences and further local index search of unaligned fragments.

Availability of supporting data

The RNA-Seq data was deposited in the SRA database from the publicly available repository NCBI, https://www.ncbi.nlm.nih.gov/sra/?term=SRA496374. Further supporting data are available in the *GigaScience* repository, GigaDB [66].

Declarations

List of abbreviations

BUSCO benchmarking universal single-copy orthologs

cds coding DNA sequence

cor Use of error corrected reads by Rcorrector

FM-index Full-text index in Minute space

ORF Open Reading Frame

skwr Skewer-trimmed reads

trimm Trimmomatic-trimmed reads

Uc Use of not corrected (or uncorrected) reads

VA *Vaccinium arboreum*

VC *Vaccinium corymbosum*

Competing interests

The authors declare no competing financial interests.

Funding

This research was supported by the National Institute of Food and Agriculture, U.S. Department of Agriculture, under award number 2009–02533 and the Thad Cochran Southern Horticultural Laboratory, U. S. Department of Agriculture Agricultural Research Service, under NACA agreement number 58–6062–5-004.

Author Contributions

GN, JO and TR prepared the biological material and collected sequencing data. MS and MPM conceived and designed the analysis workflow. MPM performed computational analysis of the data. MPM and MS analyzed the results and prepared figures. MPM and MS contributed to the writing of the manuscript. All authors read and approved the final manuscript.

Acknowledgements

We thank R.L. Darnell and V. Jones for their guidance and support in development of hydroponic experiments, H.P. Rodriguez-Armenta and W.R. Collante for their skillful support in RNA extraction and preparing libraries for sequencing, and Sun Xiaocun for support in statistics.

References

1. Conesa A, Madrigal P, Tarazona S, Gomez-Cabrero D, Cervera A, McPherson A, et al. A survey of best practices for RNA-seq data analysis. Genome Biol. 2016;17:13. doi:10.1186/s13059-016-0881-8.
2. Lindner R and Friedel CC. A comprehensive evaluation of alignment algorithms in the context of RNA-seq. PLoS One. 2012;7 12:e52403. doi:10.1371/journal.pone.0052403.
3. Engstrom PG, Steijger T, Sipos B, Grant GR, Kahles A, Ratsch G, et al. Systematic evaluation of spliced alignment programs for RNA-seq data. Nat Methods. 2013;10 12:1185-91. doi:10.1038/nmeth.2722.
4. Wang S and Gribskov M. Comprehensive evaluation of de novo transcriptome assembly programs and their effects on differential gene expression analysis. Bioinformatics. 2017;33 3:327-33. doi:10.1093/bioinformatics/btw625.

5. Nookaew I, Papini M, Pornputtapong N, Scalcinati G, Fagerberg L, Uhlen M, et al. A comprehensive comparison of RNA-Seq-based transcriptome analysis from reads to differential gene expression and cross-comparison with microarrays: a case study in Saccharomyces cerevisiae. Nucleic Acids Res. 2012;40 20:10084-97. doi:10.1093/nar/gks804.
6. Goodwin S, McPherson JD and McCombie WR. Coming of age: ten years of next-generation sequencing technologies. Nat Rev Genet. 2016;17 6:333-51. doi:10.1038/nrg.2016.49.
7. Gonzalez-Garay ML. Introduction to Isoform Sequencing Using Pacific Biosciences Technology (Iso-Seq). Transl Bioinform. 2016;9:141-60. doi:10.1007/978-94-017-7450-5_6.
8. Song L and Florea L. Rcorrector: efficient and accurate error correction for Illumina RNA-seq reads. Gigascience. 2015;4:48. doi:10.1186/s13742-015-0089-y.
9. Macmanes MD. On the optimal trimming of high-throughput mRNA sequence data. Front Genet. 2014;5:13. doi:10.3389/fgene.2014.00013.
10. da Fonseca RR, Albrechtsen A, Themudo GE, Ramos-Madrigal J, Sibbesen JA, Maretty L, et al. Next-generation biology: Sequencing and data analysis approaches for non-model organisms. Mar Genomics. 2016;30:3-13. doi:10.1016/j.margen.2016.04.012.
11. Staiger D and Brown JWS. Alternative Splicing at the Intersection of Biological Timing, Development, and Stress Responses. Plant Cell. 2013;25 10:3640-56. doi:10.1105/tpc.113.113803.
12. Smith-Unna R, Boursnell C, Patro R, Hibberd JM and Kelly S. TransRate: reference-free quality assessment of de novo transcriptome assemblies. Genome Res. 2016;26 8:1134-44. doi:10.1101/gr.196469.115.
13. Martin JA and Wang Z. Next-generation transcriptome assembly. Nat Rev Genet. 2011;12 10:671-82. doi:10.1038/nrg3068.
14. Haas BJ, Papanicolaou A, Yassour M, Grabherr M, Blood PD, Bowden J, et al. De novo transcript sequence reconstruction from RNA-seq using the Trinity platform for reference generation and analysis. Nat Protoc. 2013;8 8:1494-512. doi:10.1038/nprot.2013.084.
15. Zhao QY, Wang Y, Kong YM, Luo D, Li X and Hao P. Optimizing de novo transcriptome assembly from short-read RNA-Seq data: a comparative study. BMC Bioinformatics. 2011;12 Suppl 14:S2. doi:10.1186/1471-2105-12-S14-S2.
16. Robertson G, Schein J, Chiu R, Corbett R, Field M, Jackman SD, et al. De novo assembly and analysis of RNA-seq data. Nat Methods. 2010;7 11:909-U62. doi:10.1038/nmeth.1517.
17. Xie YL, Wu GX, Tang JB, Luo RB, Patterson J, Liu SL, et al. SOAPdenovo-Trans: de novo transcriptome assembly with short RNA-Seq reads. Bioinformatics. 2014;30 12:1660-6. doi:10.1093/bioinformatics/btu077.
18. Fu L, Niu B, Zhu Z, Wu S and Li W. CD-HIT: accelerated for clustering the next-generation sequencing data. Bioinformatics. 2012;28 23:3150-2. doi:10.1093/bioinformatics/bts565.
19. Srivastava A, Sarkar H, Malik L and Patro R. Accurate, Fast and Lightweight Clustering of de novo Transcriptomes using Fragment Equivalence Classes. arXiv preprint arXiv:160403250. 2016.
20. O'Neil ST and Emrich SJ. Assessing De Novo transcriptome assembly metrics for consistency and utility. Bmc Genomics. 2013;14 doi:Artn 465

10.1186/1471-2164-14-465.

21. Simao FA, Waterhouse RM, Ioannidis P, Kriventseva EV and Zdobnov EM. BUSCO: assessing genome assembly and annotation completeness with single-copy orthologs. Bioinformatics. 2015;31 19:3210-2. doi:10.1093/bioinformatics/btv351.

22. Li B, Fillmore N, Bai Y, Collins M, Thomson JA, Stewart R, et al. Evaluation of de novo transcriptome assemblies from RNA-Seq data. Genome Biol. 2014;15 12:553. doi:10.1186/s13059-014-0553-5.

23. Thankaswamy-Kosalai S, Sen P and Nookaew I. Evaluation and assessment of read-mapping by multiple next-generation sequencing aligners based on genome-wide characteristics. Genomics. 2017;109 3-4:186-91. doi:10.1016/j.ygeno.2017.03.001.

24. Benjamin AM, Nichols M, Burke TW, Ginsburg GS and Lucas JE. Comparing reference-based RNA-Seq mapping methods for non-human primate data. BMC Genomics. 2014;15:570. doi:10.1186/1471-2164-15-570.

25. Paya-Milans M, Nunez GH, Olmstead JW, Rinehart TA and Staton M. Regulation of gene expression in roots of the pH-sensitive Vaccinium corymbosum and the pH-tolerant Vaccinium arboreum in response to near neutral pH stress using RNA-Seq. Bmc Genomics. 2017;18 doi:ARTN 580

10.1186/s12864-017-3967-0.

26. Hirakawa H, Shirasawa K, Kosugi S, Tashiro K, Nakayama S, Yamada M, et al. Dissection of the octoploid strawberry genome by deep sequencing of the genomes of Fragaria species. DNA research : an international journal for rapid publication of reports on genes and genomes. 2014;21 2:169-81. doi:10.1093/dnares/dst049.

27. Li F, Fan G, Lu C, Xiao G, Zou C, Kohel RJ, et al. Genome sequence of cultivated Upland cotton (Gossypium hirsutum TM-1) provides insights into genome evolution. Nature biotechnology. 2015;33 5:524-30. doi:10.1038/nbt.3208.

28. International Wheat Genome Sequencing C. A chromosome-based draft sequence of the hexaploid bread wheat (Triticum aestivum) genome. Science. 2014;345 6194:1251788. doi:10.1126/science.1251788.

29. Yang J, Moeinzadeh MH, Kuhl H, Helmuth J, Xiao P, Haas S, et al. Haplotype-resolved sweet potato genome traces back its hexaploidization history. Nature plants. 2017;3 9:696-703. doi:10.1038/s41477-017-0002-z.

30. Genome sequence and analysis of the tuber crop potato. Nature. 2011;475 7355:189-95. doi:http://www.nature.com/nature/journal/v475/n7355/abs/nature10158-f1.2.html - supplementary-information.

31. Huang S, Ding J, Deng D, Tang W, Sun H, Liu D, et al. Draft genome of the kiwifruit Actinidia chinensis. Nature communications. 2013;4:2640. doi:10.1038/ncomms3640.

32. Bertioli DJ, Cannon SB, Froenicke L, Huang G, Farmer AD, Cannon EK, et al. The genome sequences of Arachis duranensis and Arachis ipaensis, the diploid ancestors of cultivated peanut. Nature genetics. 2016;48 4:438-46. doi:10.1038/ng.3517.

33. Gupta V, Estrada AD, Blakley I, Reid R, Patel K, Meyer MD, et al. RNA-Seq analysis and annotation of a draft blueberry genome assembly identifies candidate genes involved in fruit ripening, biosynthesis of bioactive compounds,

and stage-specific alternative splicing. Gigascience. 2015;4:5. doi:10.1186/s13742-015-0046-9.

34. Hancock JF, Lyrene P, Finn CE, Vorsa N and Lobos GA. Blueberries and cranberries. Temperate fruit crop breeding. Springer; 2008. p. 115-50.

35. Bian Y, Ballington J, Raja A, Brouwer C, Reid R, Burke M, et al. Patterns of simple sequence repeats in cultivated blueberries (Vaccinium section Cyanococcus spp.) and their use in revealing genetic diversity and population structure. Molecular Breeding. 2014;34 2:675-89. doi:10.1007/s11032-014-0066-7.

36. Bolger AM, Lohse M and Usadel B. Trimmomatic: a flexible trimmer for Illumina sequence data. Bioinformatics. 2014;30 15:2114-20. doi:10.1093/bioinformatics/btu170.

37. Jiang H, Lei R, Ding SW and Zhu S. Skewer: a fast and accurate adapter trimmer for next-generation sequencing paired-end reads. BMC Bioinformatics. 2014;15:182. doi:10.1186/1471-2105-15-182.

38. Heydari M, Miclotte G, Demeester P, Van de Peer Y and Fostier J. Evaluation of the impact of Illumina error correction tools on de novo genome assembly. BMC Bioinformatics. 2017;18 1:374. doi:10.1186/s12859-017-1784-8.

39. Ramirez-Sanchez O, Perez-Rodriguez P, Delaye L and Tiessen A. Plant Proteins Are Smaller Because They Are Encoded by Fewer Exons than Animal Proteins. Genomics Proteomics Bioinformatics. 2016;14 6:357-70. doi:10.1016/j.gpb.2016.06.003.

40. Hoang NV, Furtado A, Mason PJ, Marquardt A, Kasirajan L, Thirugnanasambandam PP, et al. A survey of the complex transcriptome from the highly polyploid sugarcane genome using full-length isoform sequencing and de novo assembly from short read sequencing. BMC Genomics. 2017;18 1:395. doi:10.1186/s12864-017-3757-8.

41. Visser EA, Wegrzyn JL, Steenkmap ET, Myburg AA and Naidoo S. Combined de novo and genome guided assembly and annotation of the Pinus patula juvenile shoot transcriptome. BMC Genomics. 2015;16:1057. doi:10.1186/s12864-015-2277-7.

42. Del Fabbro C, Scalabrin S, Morgante M and Giorgi FM. An extensive evaluation of read trimming effects on Illumina NGS data analysis. PLoS One. 2013;8 12:e85024. doi:10.1371/journal.pone.0085024.

43. Duan J, Xia C, Zhao G, Jia J and Kong X. Optimizing de novo common wheat transcriptome assembly using short-read RNA-Seq data. BMC Genomics. 2012;13:392. doi:10.1186/1471-2164-13-392.

44. Chandra S, Singh D, Pathak J, Kumari S, Kumar M, Poddar R, et al. De Novo Assembled Wheat Transcriptomes Delineate Differentially Expressed Host Genes in Response to Leaf Rust Infection. PLoS One. 2016;11 2:e0148453. doi:10.1371/journal.pone.0148453.

45. Chow KS, Ghazali AK, Hoh CC and Mohd-Zainuddin Z. RNA sequencing read depth requirement for optimal transcriptome coverage in Hevea brasiliensis. BMC Res Notes. 2014;7:69. doi:10.1186/1756-0500-7-69.

46. Li H and Homer N. A survey of sequence alignment algorithms for next-generation sequencing. Briefings in Bioinformatics. 2010;11 5:473-83. doi:10.1093/bib/bbq015.

47. Smith TF and Waterman MS. Identification of common molecular subsequences. J Mol Biol. 1981;147 1:195-7.

48. Myers G. A fast bit-vector algorithm for approximate string matching based on dynamic programming. Journal of the Acm. 1999;46 3:395-415. doi:Doi 10.1145/316542.316550.
49. Andrews S: FastQC. http://www.bioinformatics.babraham.ac.uk/projects/fastqc/.
50. Trapnell C, Hendrickson DG, Sauvageau M, Goff L, Rinn JL and Pachter L. Differential analysis of gene regulation at transcript resolution with RNA-seq. Nat Biotechnol. 2013;31 1:46-53. doi:10.1038/nbt.2450.
51. Patro R, Duggal G, Love MI, Irizarry RA and Kingsford C. Salmon provides fast and bias-aware quantification of transcript expression. Nat Methods. 2017;14 4:417-9. doi:10.1038/nmeth.4197.
52. Wu TD, Reeder J, Lawrence M, Becker G and Brauer MJ. GMAP and GSNAP for Genomic Sequence Alignment: Enhancements to Speed, Accuracy, and Functionality. Methods Mol Biol. 2016;1418:283-334. doi:10.1007/978-1-4939-3578-9_15.
53. Quinlan AR and Hall IM. BEDTools: a flexible suite of utilities for comparing genomic features. Bioinformatics. 2010;26 6:841-2. doi:10.1093/bioinformatics/btq033.
54. Jay JJ, Eblen JD, Zhang Y, Benson M, Perkins AD, Saxton AM, et al. A systematic comparison of genome-scale clustering algorithms. BMC Bioinformatics. 2012;13 Suppl 10:S7. doi:10.1186/1471-2105-13-S10-S7.
55. Haas B and Papanicolaou A: TransDecoder (Find Coding Regions Within Transcripts). https://transdecoder.github.io/.
56. Altschul SF, Gish W, Miller W, Myers EW and Lipman DJ. Basic local alignment search tool. J Mol Biol. 1990;215 3:403-10. doi:10.1016/S0022-2836(05)80360-2.
57. Finn RD, Coggill P, Eberhardt RY, Eddy SR, Mistry J, Mitchell AL, et al. The Pfam protein families database: towards a more sustainable future. Nucleic Acids Research. 2016;44 D1:D279-D85. doi:10.1093/nar/gkv1344.
58. HMMER 3.1b2. http://hmmer.org/.
59. BroadInstitute: Picard Tools. https://github.com/broadinstitute/picard (2017).
60. DeLuca DS, Levin JZ, Sivachenko A, Fennell T, Nazaire MD, Williams C, et al. RNA-SeQC: RNA-seq metrics for quality control and process optimization. Bioinformatics. 2012;28 11:1530-2. doi:10.1093/bioinformatics/bts196.
61. Anders S, Pyl PT and Huber W. HTSeq-a Python framework to work with high-throughput sequencing data. Bioinformatics. 2015;31 2:166-9. doi:10.1093/bioinformatics/btu638.
62. Langmead B and Salzberg SL. Fast gapped-read alignment with Bowtie 2. Nature Methods. 2012;9 4:357-U54. doi:10.1038/Nmeth.1923.
63. Lunter G and Goodson M. Stampy: A statistical algorithm for sensitive and fast mapping of Illumina sequence reads. Genome Research. 2011;21 6:936-9. doi:10.1101/gr.111120.110.
64. Dobin A, Davis CA, Schlesinger F, Drenkow J, Zaleski C, Jha S, et al. STAR: ultrafast universal RNA-seq aligner. Bioinformatics. 2013;29 1:15-21. doi:10.1093/bioinformatics/bts635.
65. Kim D, Langmead B and Salzberg SL. HISAT: a fast spliced aligner with low memory requirements. Nat Methods. 2015;12 4:357-60. doi:10.1038/nmeth.3317.

66. Payá-Milans M; Olmstead JW; Nunez G; Rinehart TA; Staton M: Supporting data for "Comprehensive evaluation of RNA-Seq analysis pipelines in diploid and polyploid species" GigaScience Database. 2018. http://dx.doi.org/10.5524/100517

Supplementary data

Figure S1

.jpg

Diagram representing the *de novo* assembly strategies, run independently for each *Vaccinium* species. The set of control and treatment reads produced by different correction and trimming strategies were used as input. The control read files were assembled (A) independently as were the treatment read files (B). From here, each set of control sample transcripts was combined with the treatment sample transcripts (i.e. the Skewer corrected control transcripts were merged with the Skewer corrected treatment transcripts, the Trimmomatic uncorrected control transcripts were merged with the Trimmomatic uncorrected treatment transcripts, etc.) (C). These merged transcript sets were then clustered with either CD-HIT (D) or RapClust (E). This results in eight clustered assemblies. A second assembly strategy merged the control and treatment reads prior to assembly (F). These sets of transcripts were also clustered with either CD-HIT (G) or RapClust (H), also resulting in another set of eight clustered assemblies.

Figure S2

.tiff

Subdivision in categories of reads mapped to the reference genome performed by HTSeq. Except in the case of STAR, which does not report not mapped reads, height of bars up to red resembles the number of trimmed reads. Options are ordered by correction state, mismatch tolerance options and trimming software.

Figure S3

.tiff

Mapping results to the reference genome categorized by overlapping gene feature.

Figure S4

.pdf

Subdivision in categories of reads mapped to *de novo* assemblies performed by HTSeq. In specific cases with HISAT2 and STAR, multiple aligned reads are counted multiple times, overestimating the total number of reads. Options are ordered by correction state, trimming software and type of assembly.

Table S1

.xlsx

Description of main algorithms used on this work.

Brief algorithmic explanations, software claims and major findings are included for programs tested at (A) pre-processing of RNA-Seq reads, (B) *de novo* assembly of transcriptomes and redundancy reduction by clustering, and (C) mapping of short reads to both blueberry reference genome and Trinity assemblies clustered with CD-HIT. BWT, Burrows-Wheeler Transform; FM-index, Full-text index in Minute space.

Table S2

.xlsx

Variation in number and length of reads after pre-processing.

Number of reads before and after trimming with either Skewer or Trimmomatic and using (cor) or not (Uc) error correction. Last column indicate average length of reads after trimming the 101-bp raw reads. Values are mean ± sd of 8 samples.

Table S3

.txt

Mapping and annotation metrics of Trinity clustered assemblies to *V. corymbosum* reference genome.

Transcripts mapped either uniquely to the genome (uniq), to multiple locations (mult), with translocations (transloc) or did not map (out). Subdivision based on annotation includes “All mapping transcripts” (map), “Mapping transcripts with CDS” (cds) and “CDS with blast hit” (blast).

Table S4

.xslx

Read mapping rates.

Proportion of reads mapped from each combination of error correction, trimming software, mismatch tolerance or assembly samples, when appropriate, to either the reference genome or *de novo* assemblies after clustering with CD-HIT.

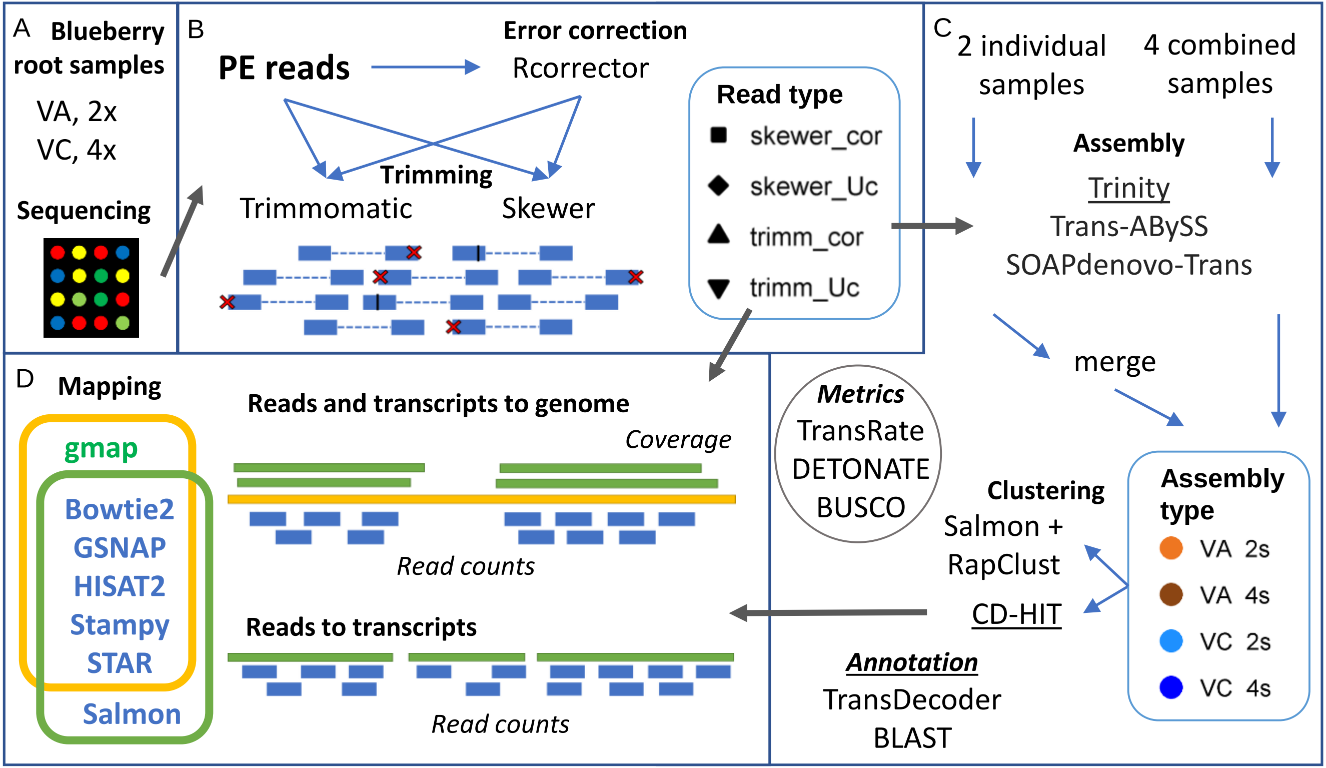
A
Blueberry root samples
VA, 2x
VC, 4x
Sequencing
B
PE reads
Error correction
Rcorrector
Trimming
Trimmomatic
Skewer
Read type
skewer_cor
skewer_Uc
trimm_cor
trimm_Uc
C
2 individual samples
4 combined samples
Assembly
Trinity
Trans-ABySS
SOAPdenovo-Trans
merge
Metrics
TransRate
DETONATE
BUSCO
Clustering
Salmon +
RapClust
CD-HIT
Assembly type
VA 2s
VA 4s
VC 2s
VC 4s
Annotation
TransDecoder
BLAST
D
Mapping
gmap
Bowtie2
GSNAP
HISAT2
Stampy
STAR
Salmon
Reads and transcripts to genome
Coverage
Read counts
Reads to transcripts
Read counts

Click here to access/download;Figure;Fig 2.tiff

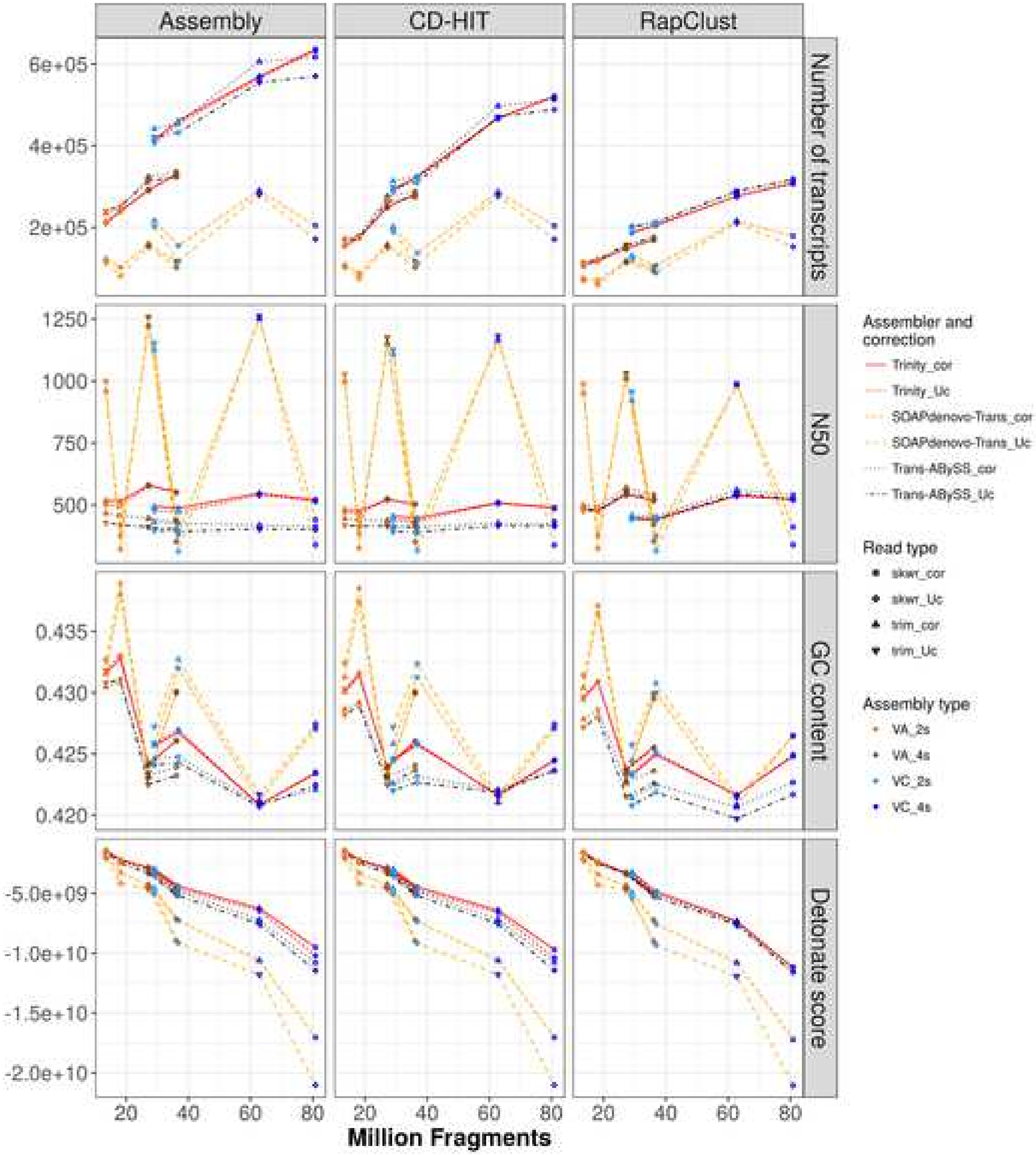

Click here to access/download;Figure;Fig 3.tiff

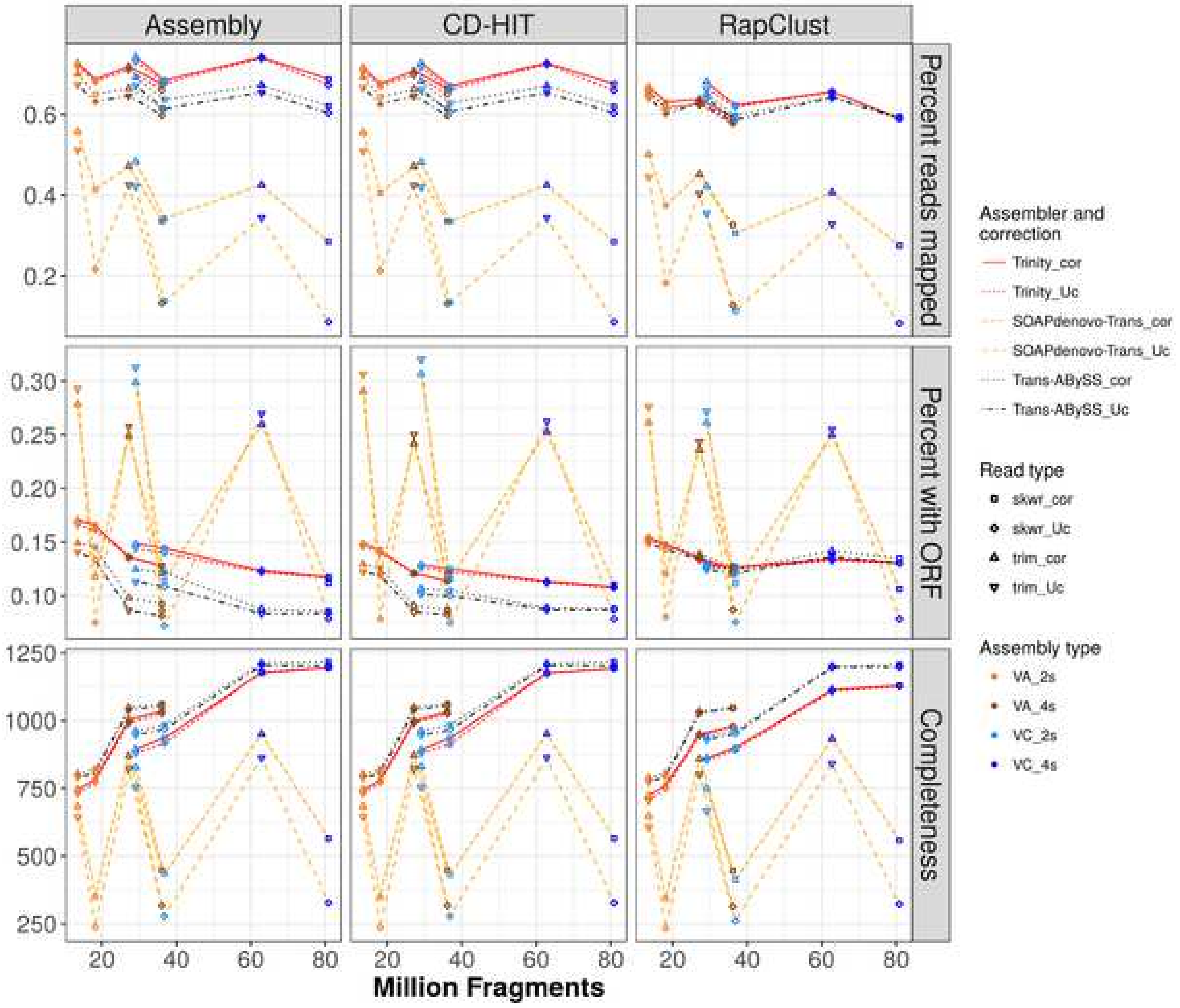

Click here to access/download;Figure;Fig 4.tiff

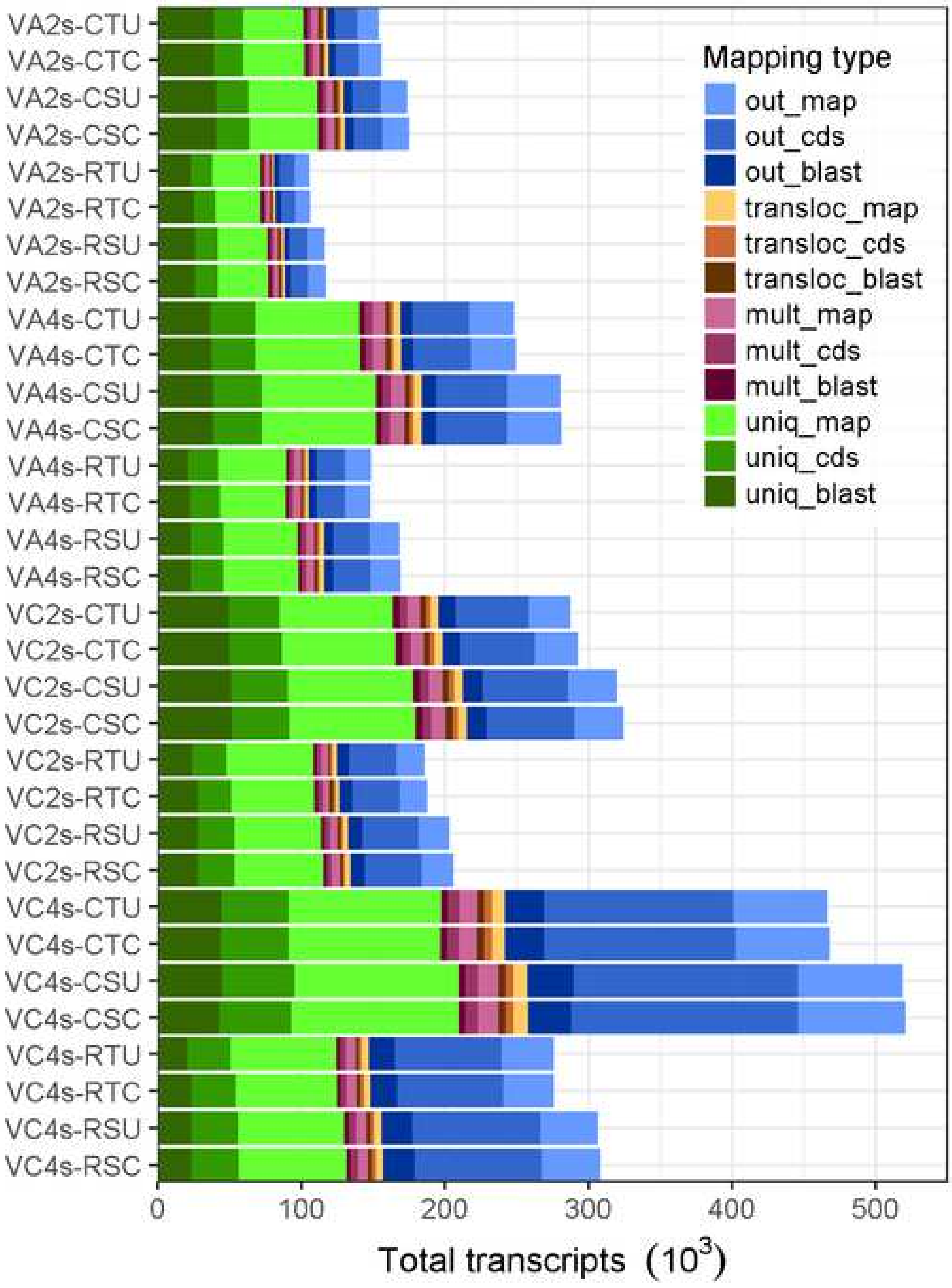

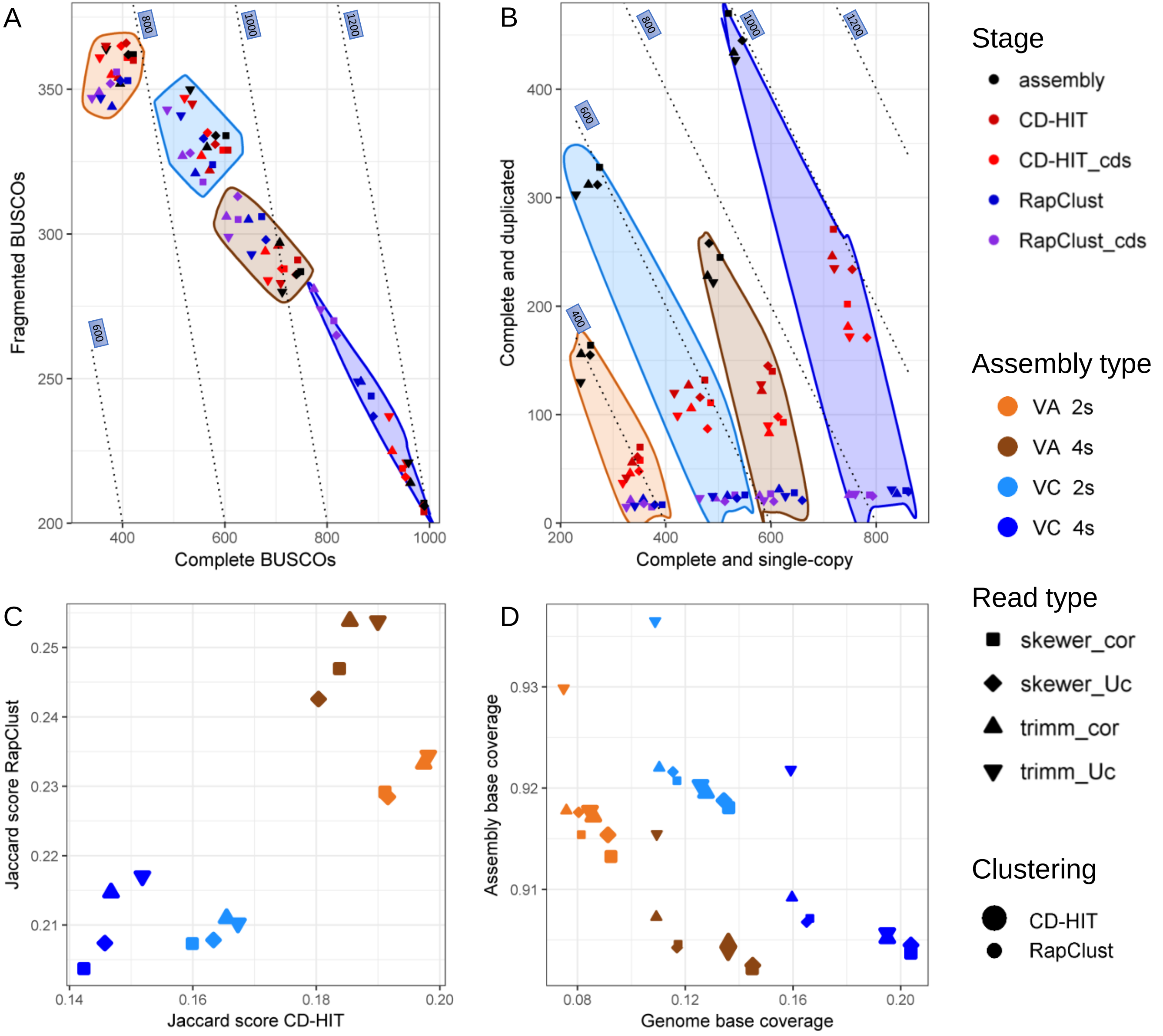
A
Fragmented BUSCOs
Complete BUSCOs
B
Complete and duplicated
Complete and single-copy
C
Jaccard score RapClust
Jaccard score CD-HIT
D
Assembly base coverage
Genome base coverage
Stage
assembly
CD-HIT
CD-HIT_cds
RapClust
RapClust_cds
Assembly type
VA 2s
VA 4s
VC 2s
VC 4s
Read type
skewer_cor
skewer_Uc
trimm_cor
trimm_Uc
Clustering
CD-HIT
RapClust

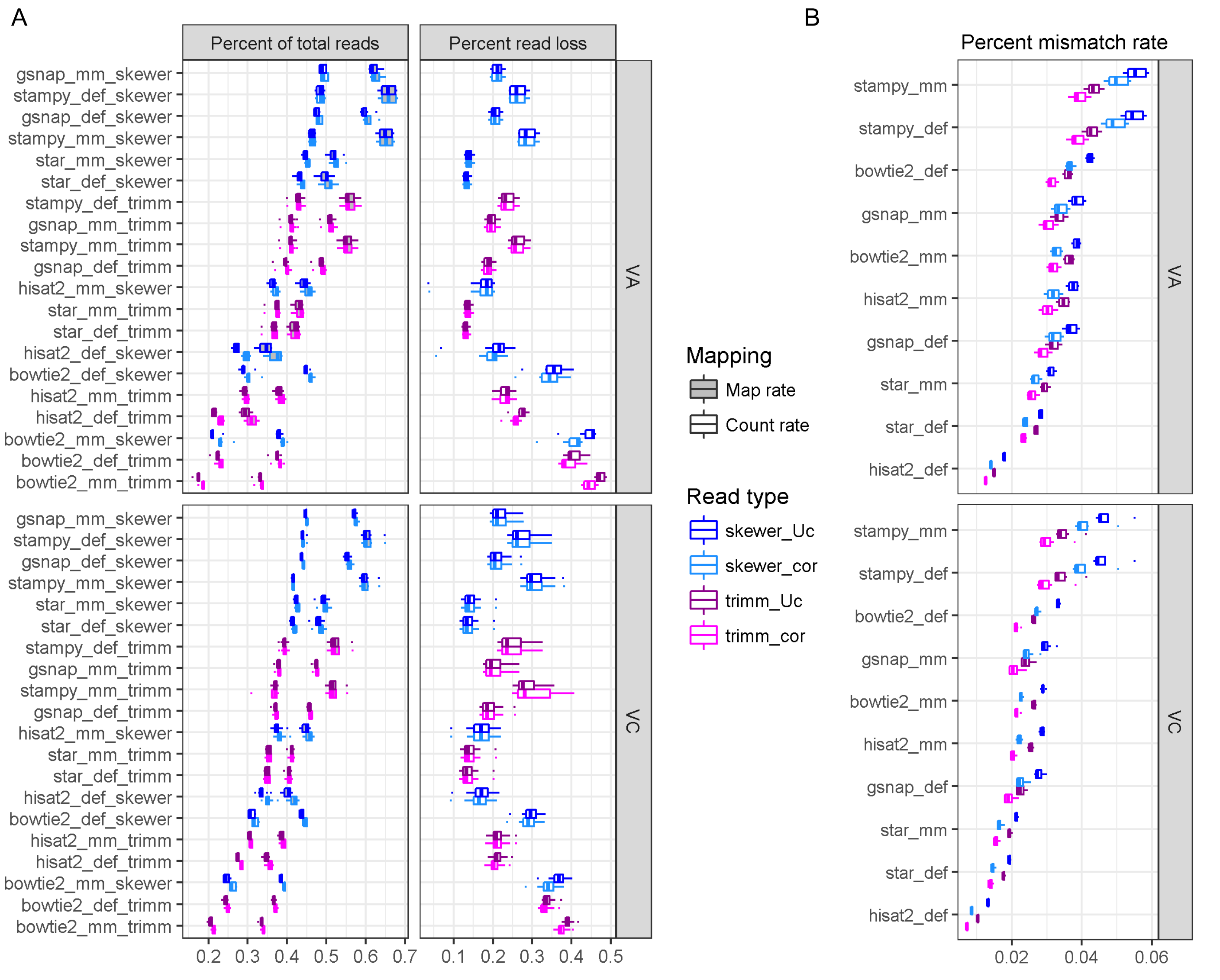
A
B
Percent of total reads
Percent read loss
Percent mismatch rate
gsnap_mm_skewer
stampy_def_skewer
gsnap_def_skewer
stampy_mm_skewer
star_mm_skewer
star_def_skewer
stampy_def_trimm
gsnap_mm_trimm
stampy_mm_trimm
gsnap_def_trimm
hisat2_mm_skewer
star_mm_trimm
star_def_trimm
hisat2_def_skewer
bowtie2_def_skewer
hisat2_mm_trimm
hisat2_def_trimm
bowtie2_mm_skewer
bowtie2_def_trimm
bowtie2_mm_trimm
VA
VC
0.2 0.3 0.4 0.5 0.6 0.7
0.1 0.2 0.3 0.4 0.5
stampy_mm
stampy_def
bowtie2_def
gsnap_mm
bowtie2_mm
hisat2_mm
gsnap_def
star_mm
star_def
hisat2_def
0.02 0.04 0.06
Mapping
Map rate
Count rate
Read type
skewer_Uc
skewer_cor
trimm_Uc
trimm_cor

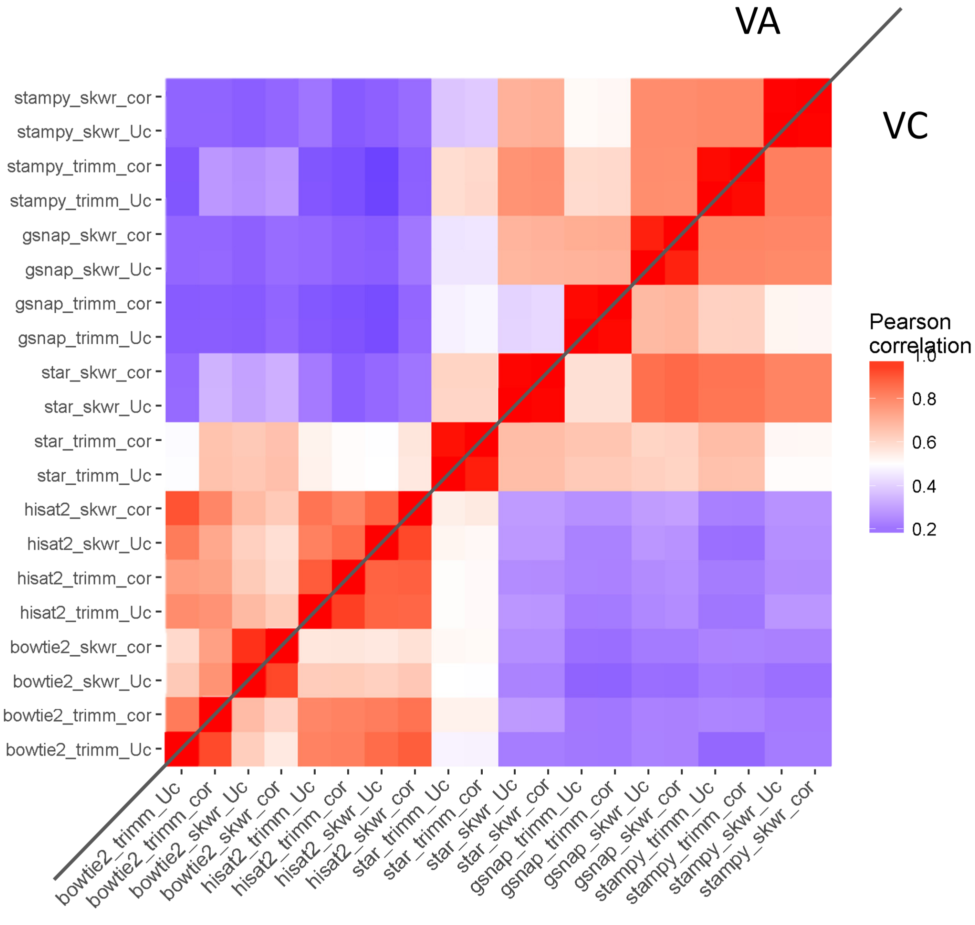
VA
VC
stampy_skwr_cor
stampy_skwr_Uc
stampy_trimm_cor
stampy_trimm_Uc
gsnap_skwr_cor
gsnap_skwr_Uc
gsnap_trimm_cor
gsnap_trimm_Uc
star_skwr_cor
star_skwr_Uc
star_trimm_cor
star_trimm_Uc
hisat2_skwr_cor
hisat2_skwr_Uc
hisat2_trimm_cor
hisat2_trimm_Uc
bowtie2_skwr_cor
bowtie2_skwr_Uc
bowtie2_trimm_cor
bowtie2_trimm_Uc
Pearson correlation
1.0
0.8
0.6
0.4
0.2

Click here to access/download;Figure;Fig 8.tiff

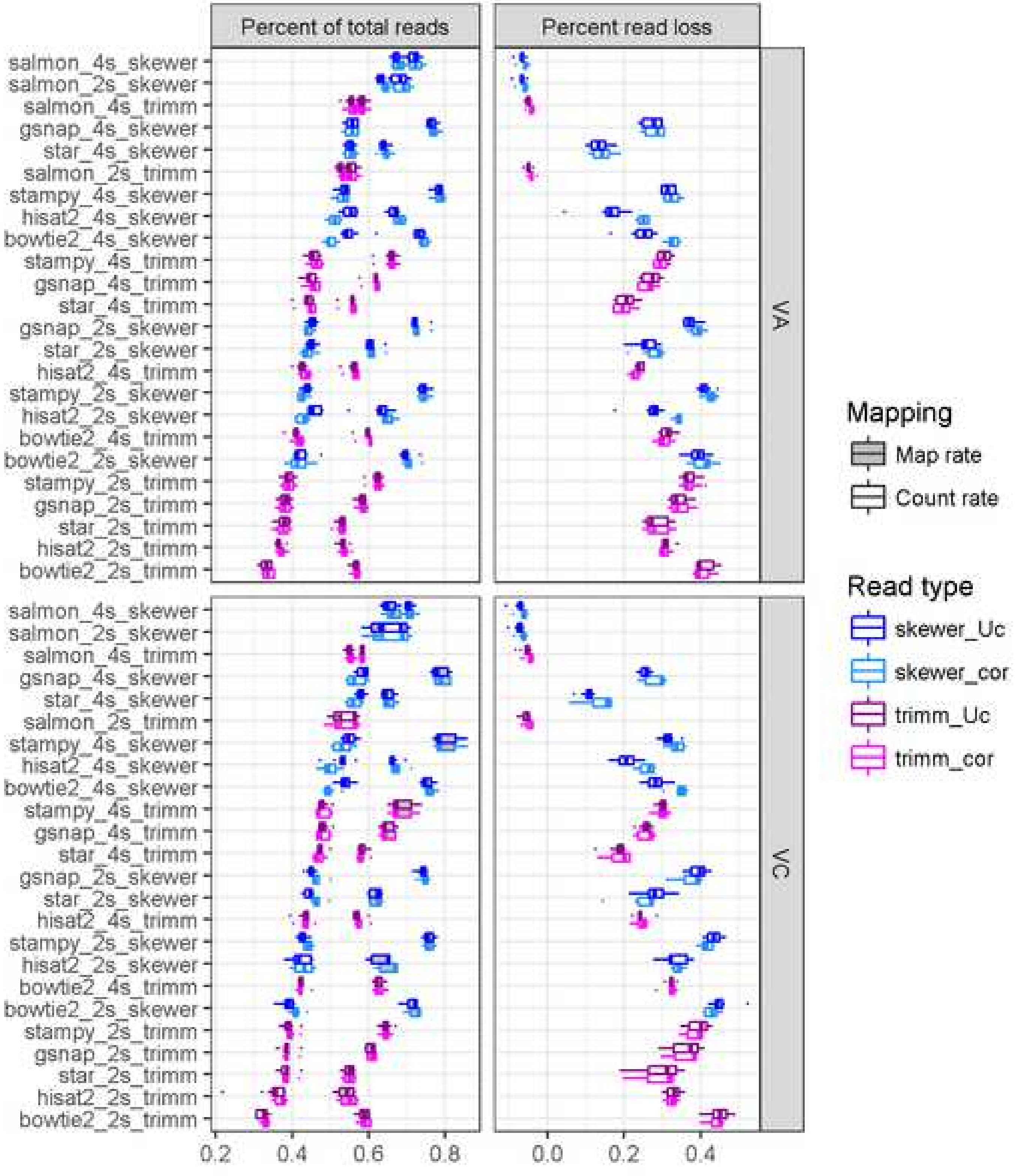

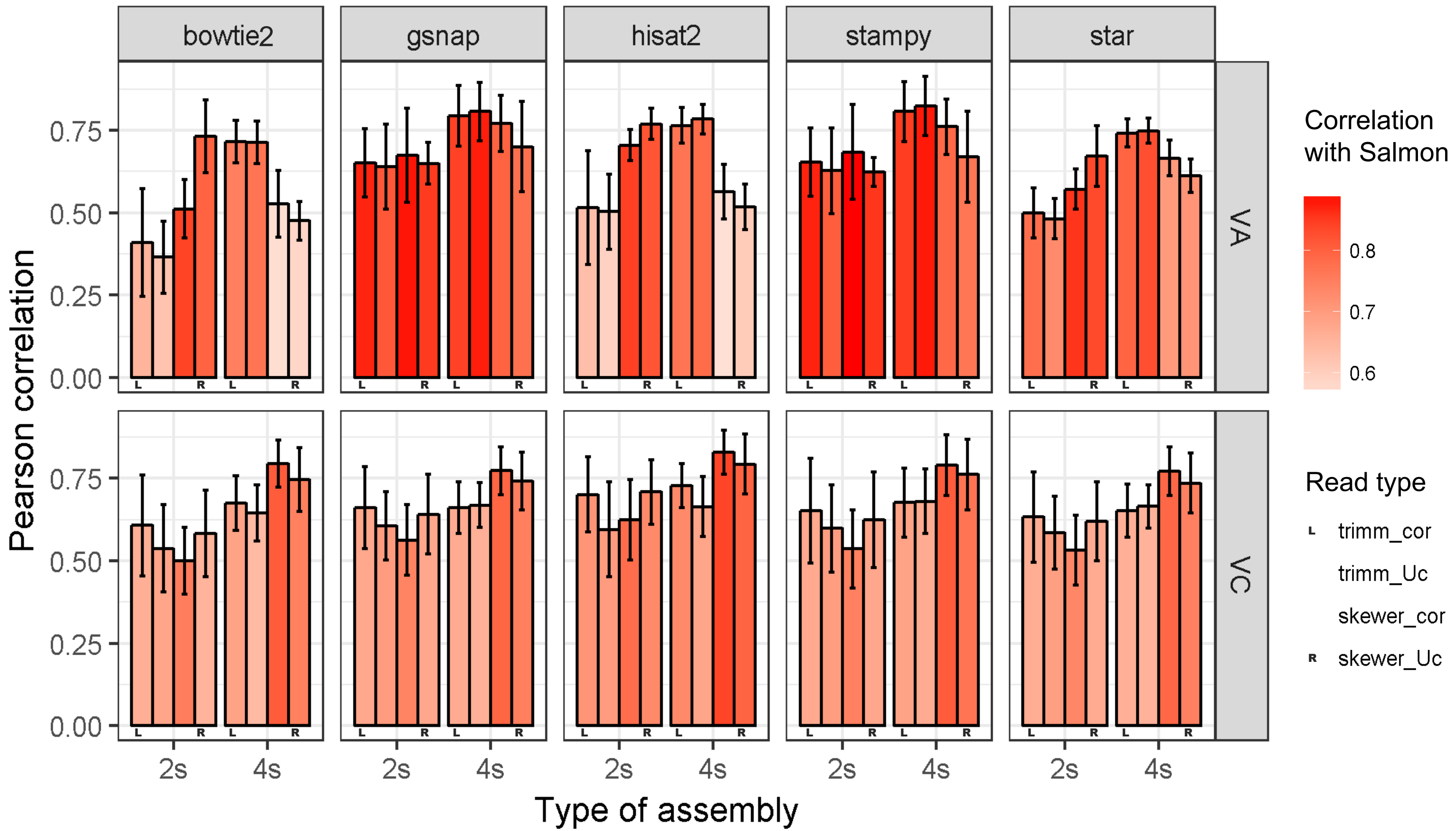
bowtie2
gsnap
hisat2
stampy
star
VA
VC
Pearson correlation
0.00
0.25
0.50
0.75
2s
4s
Type of assembly
Correlation with Salmon
0.6
0.7
0.8
Read type
L trimm_cor
trimm_Uc
skewer_cor
R skewer_Uc

Click here to access/download
Supplementary Material
Fig S1.jpg

Click here to access/download
Supplementary Material
Fig S2.tiff

Click here to access/download
Supplementary Material
Fig S3.tiff

Click here to access/download
Supplementary Material
Fig S4.tiff

Click here to access/download
Supplementary Material
Table S1.xlsx

Click here to access/download
Supplementary Material
Table S2.xlsx

Click here to access/download
Supplementary Material
Table S3.txt

Click here to access/download
Supplementary Material
Table S4.xlsx
