# Supplementary figures and images for: Comprehensive evaluation of RNA-seq analysis pipelines in diploid and polyploid species
Source: Gigascience. 2018 Nov 10;7(12):giy132. doi: 10.1093/gigascience/giy132 (PMC6275443; doi:10.1093/gigascience/giy132)

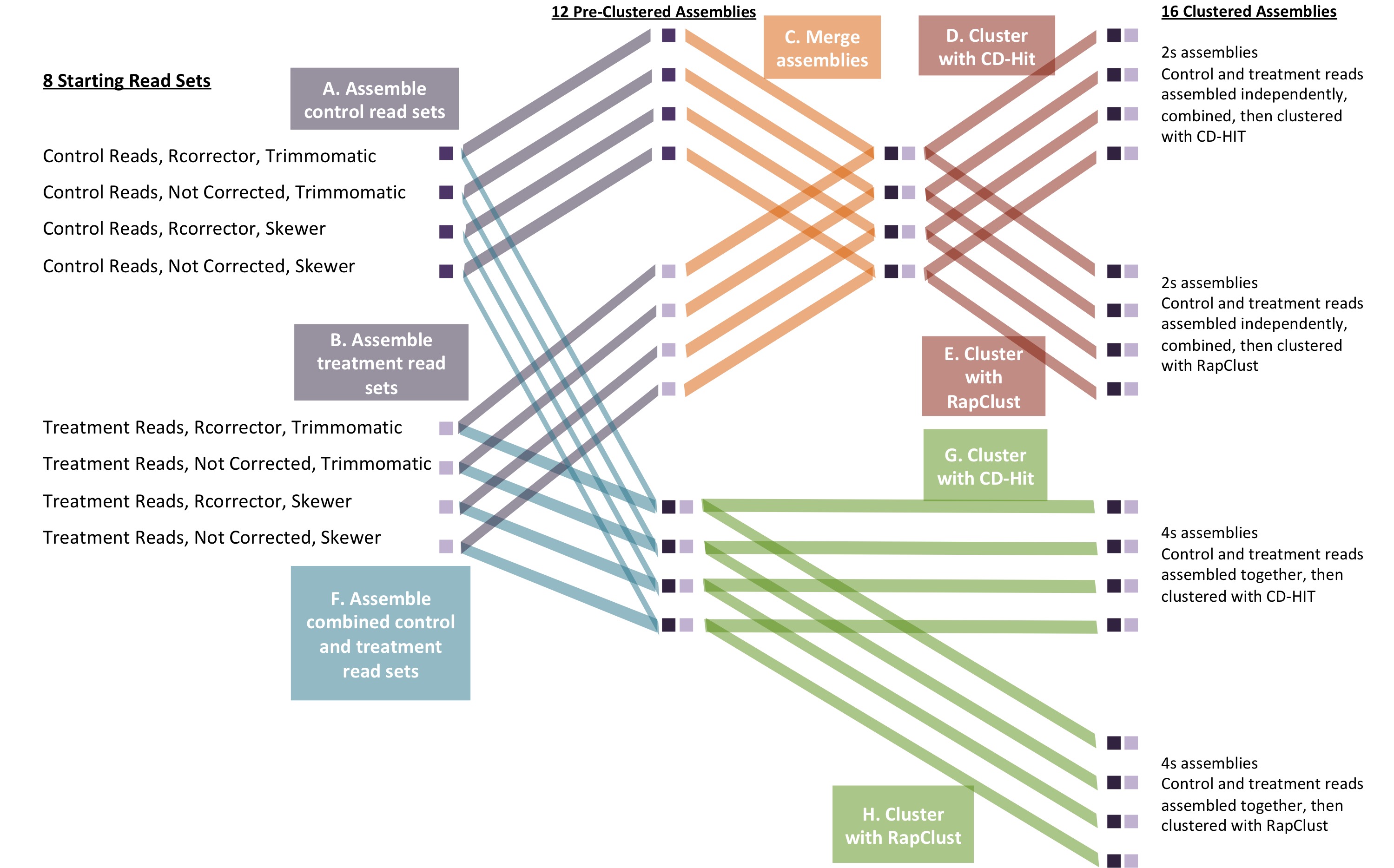

Supplement: Supplement Files [file giy132_supplement_files.zip › Fig S1.jpg]

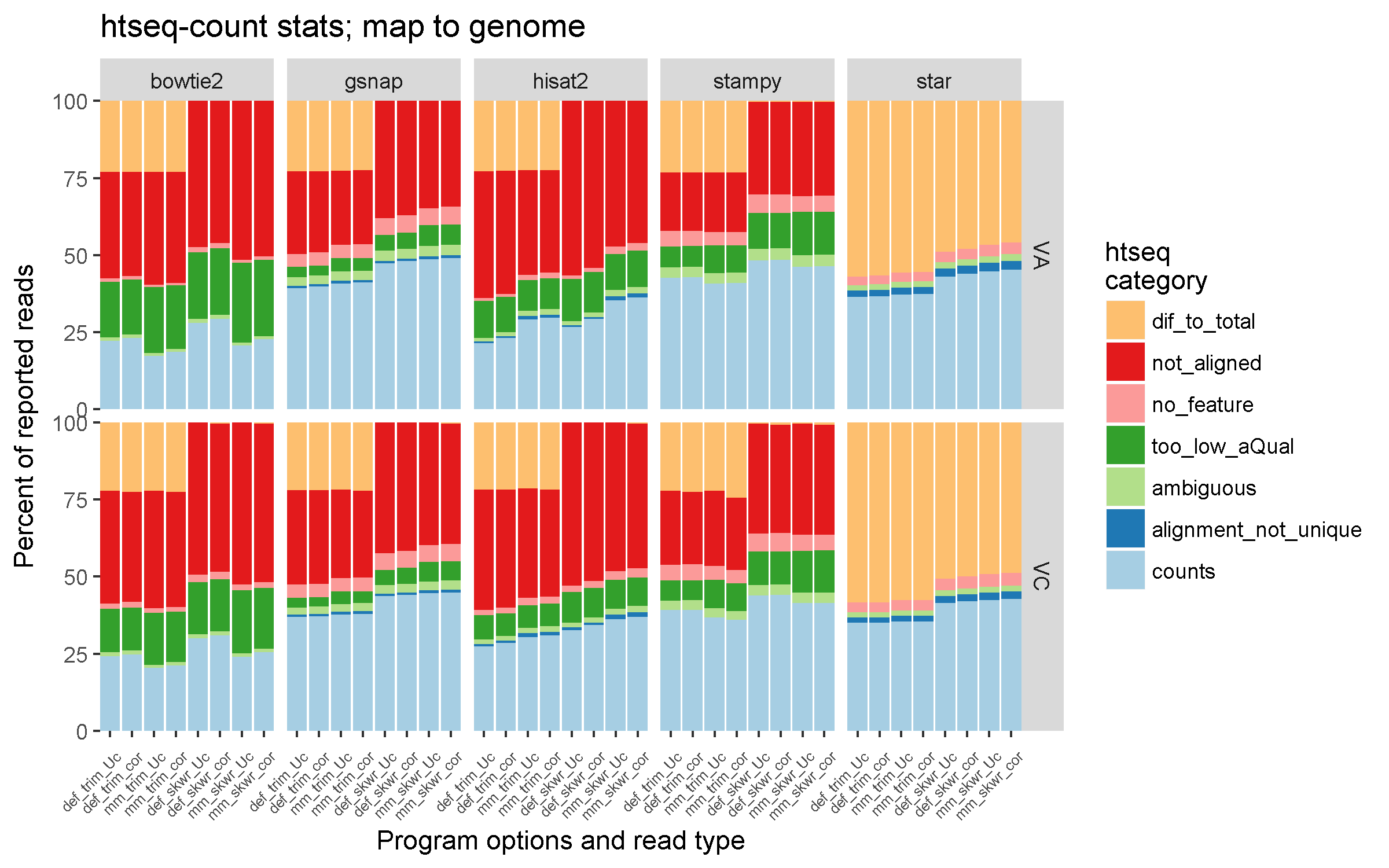

Supplement: Supplement Files [file giy132_supplement_files.zip › Fig S2.tiff]

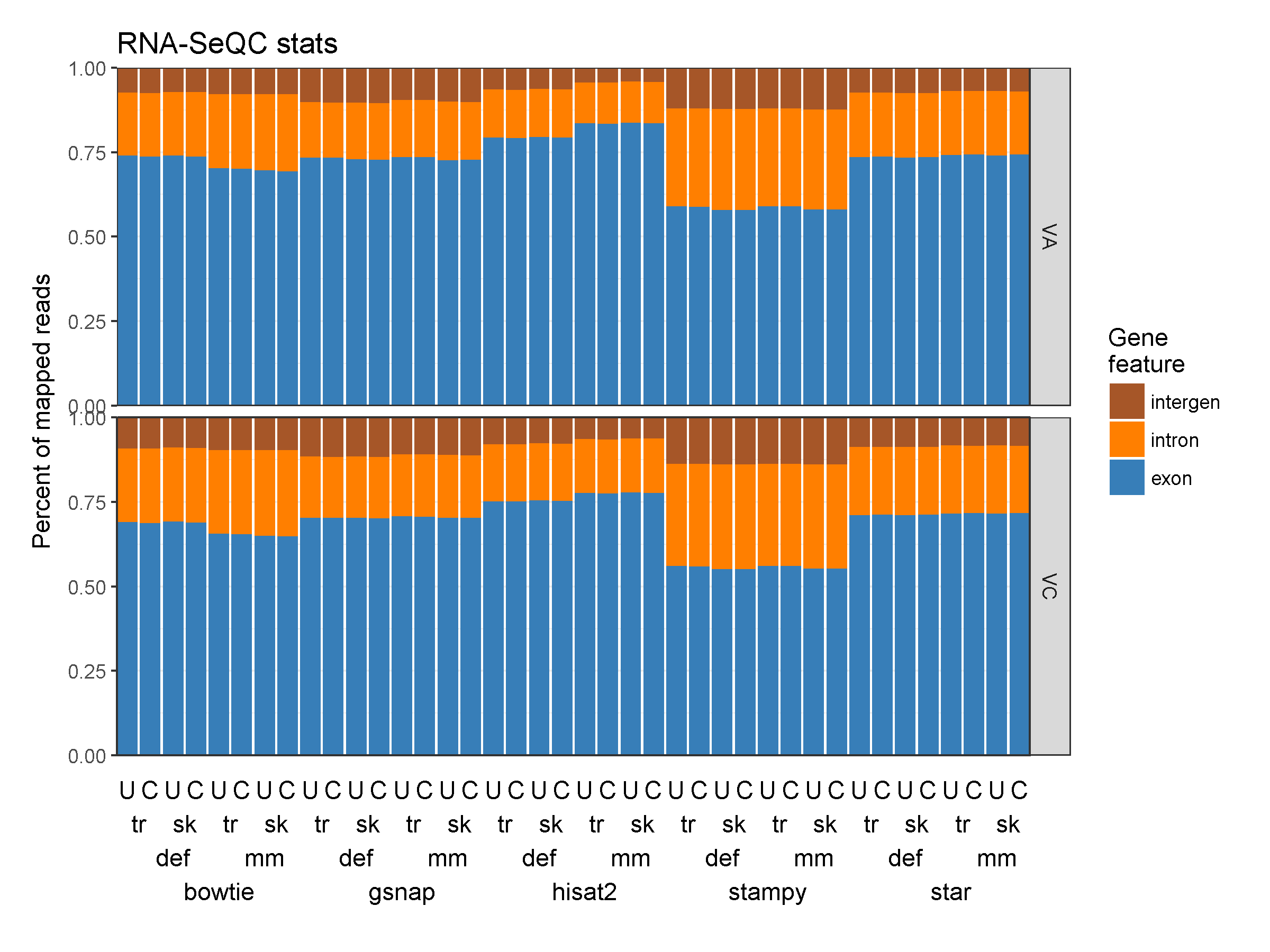

Supplement: Supplement Files [file giy132_supplement_files.zip › Fig S3.tiff]

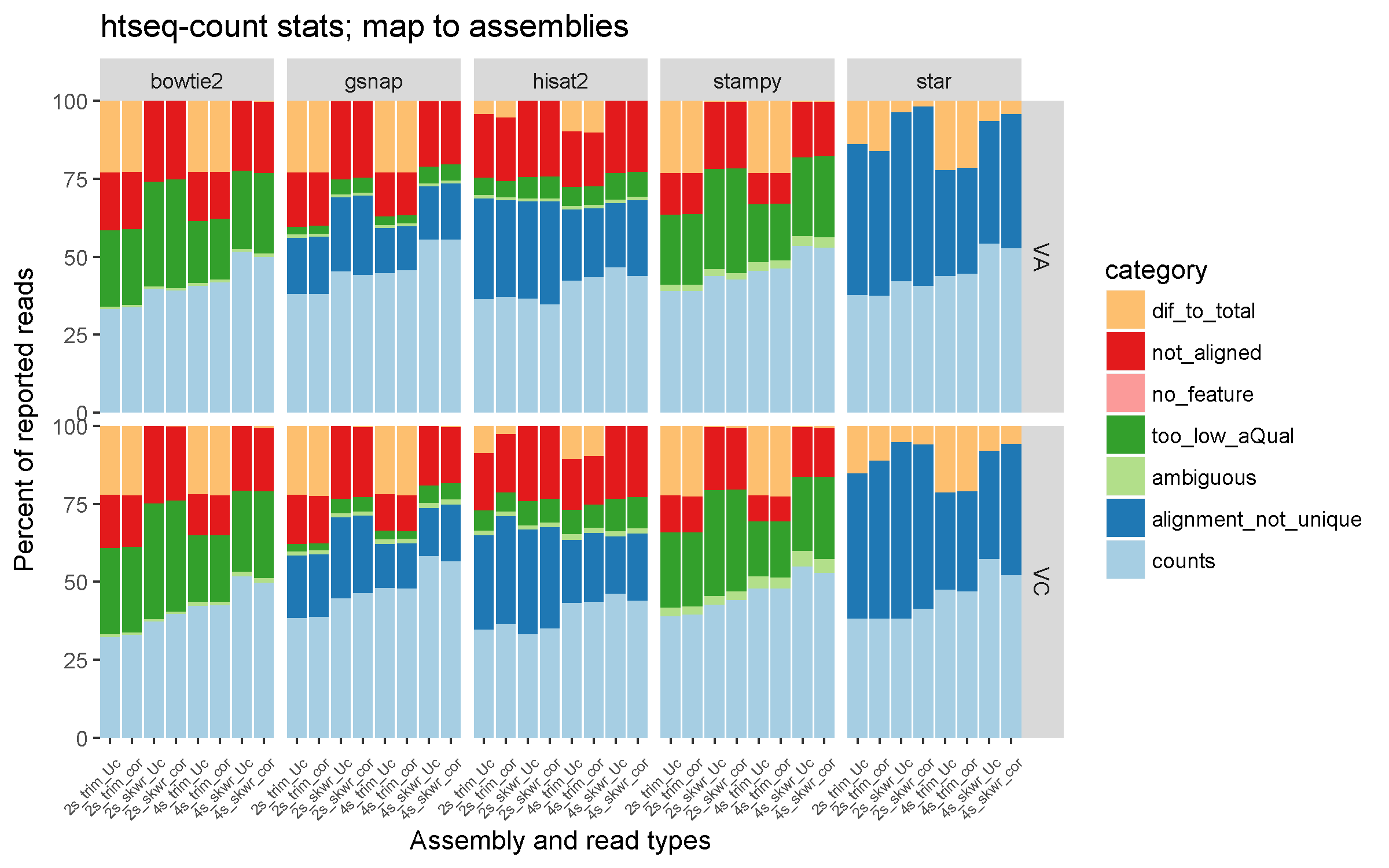

Supplement: Supplement Files [file giy132_supplement_files.zip › Fig S4.tiff]
