# Supplementary material for: Comprehensive evaluation of RNA-seq analysis pipelines in diploid and polyploid species
Source: Gigascience. 2018 Nov 10;7(12):giy132. doi: 10.1093/gigascience/giy132 (PMC6275443; doi:10.1093/gigascience/giy132)
Supplement: Response_to_Reviewer_Comments_Original_Submission.pdf [file giy132_response_to_reviewer_comments_original_submission.pdf]

Author's Response To Reviewer Comments

Close

Reviewer reports:

>Reviewer #1: General Comments.

>The idea of comparing different assembly and mapping strategies is compelling. It is true, that there are few resources about the effects of polyplody on tools designed mostly for diploids. Since the mappings are already done, you could explore in more detail how multiple homoeologues may be mapping to the same "unigene", or you could try to figure out if the homoeologues are removed/merged into single unigenes. If that is the case, you may be mapping the tetraploid to a reference closer to a diploid. If the duplication event is recent, you can expect almost double of the genes in the tetraploid transcriptome, compared to the diploid.

We attempted a comparison between transcripts from the tetraploid and diploid gene models, but results were difficult to interpret. To date, there is no tetraploid Vaccinium genome to use for the sequences for homoeolog genes to distinguish between isoforms and homoeologues. Thus, we used the BUSCO tool (benchmark universal single copy orthologs) to explore the relative duplication of transcriptomes, considering that similar homoeologues may be hits to the same BUSCO protein, and also we discuss how clustering reduces duplication; however, whether these duplicates are homoeologues or isoforms remains uncertain. In relation to when the duplication event took place, although cytogenetic studies have been done to assess blueberry ploidy (Sakhanokho 2018), we couldn’t find any information on specific timing (recent or not) in the literature. Figure 5 A&B, Lines 517-520.

>The idea behind figure 1, that shows all the tools is nice. However, it can be improved to make the order of the pipeline more explicit.

Figure 1 is modified and now contains arrows to help follow the pipeline.

>Also, the kind of algorithms, drawbacks, advantages, etc of each program used is scatter all over the place. It would be nice to have a table with all that information summarized, including one column with a short description of the final effect in each step of the analysis. A row could look like (with more rows, one for each step in the pipeline)
>Tool: Trimmomatic
>De Novo Assembly: Improves in 5% on VC (or whatever you find)
>Mapping to genome: Limited effect.

As suggested, a new supplementary table including pre-processing tools, assemblers, clustering methods and aligners has been added. (Table S1)

The figures require a lot of work to make them look consistent (same colours for same variables across the paper, for example).

>Colors have been made consistent among figures.

>Specific comments.

>Figure 1.
>General: It is confusing what are characteristics of the analysis (like individual/combined), programs (Is Rcorrector a program? A typo?). Some colour/font style change could help to distinguish them. The legend requires a lot of work, as it is not very descriptive of the elements represented. Also, the colours could be improved to reduce confusion. Yellow seems to represent "cor trim" and reference genome. Grey is for Cor skewer, but it is also used for Clustering.
>Panel A: Cor skewer is not present in the diagram. Also, there is no explanation of what the crosses mean. Rcorrector is not defined in legend. The figure seems to suggest that Rcorrector and Trimmomatic/Skewer are two different pipelines, where in the text it is described as Rcorrector+Trimmmatic or Rcorrector+Skewer
>Panel B: The boxes don't need to be colour coded, as the colours are not used elsewhere to link, and adds confusion as green are blue are used to represent transcripts and reads elsewhere in the figure.
>Panel C: It is not clear that the top and the bottom diagrams are different things (De Novo vs reference guided).

Considering the comments of the reviewer, Figure 1 has been modified and the legend is now fully descriptive.

>Line 72. Illumina may still be cheaper, but it may be worth mentioning Iso-Seq, from PacBio that are already able to retrieve full transcripts. I understand it is beyond the scope of the paper, but it is worth mentioning.

A line commenting on Iso-Seq for transcriptome studies is added (Line 85-88).

>Line 89. A supplementary figure showing how the different errors affect the assembly could help the unexperienced reader to understand why the errors happen.

A short description and an additional citation are included to help readers with this (Line 104-105).

>Line 93. It is commonly selected, agreed. But how do you define good performance? Having used it before, the pipeline writes several temporary files, which computationally is not very performant. If it refers to the quality of the assembly, no other options are discussed in this paper, are there any other RNA-Seq assemblers?

Our original goal for good performance was referring to high scores in metrics such as mapped-back reads,fewer chimeras, or good recovery of transcripts, where Trinity performs well. The review makes a good point that performance may be related to computational efficiency rather than or in addition to biological accuracy, “good performance” is changed to “good quality”. (Line 112). Also, a pair of extra assemblers are added to the analysis as requested by another reviewer, please see below.

>Line 112. FM-Index is not defined. Hash tables are considered fast in computer science. You can argue that it depends on details of the implementations and how the different software compensate for the drawbacks (like doing a "proper " alignment once the region

where the read maps is identified).

The description has been added, and also the sentence was modified to indicate array and algorithm on the comparison. (Line 135-138)

>Line 136: Is Vaccinium corymbosum derived from a duplication of V. arboreum? if so, it may be worth to mention. It would also be nice to have a comparison of how distant they are.

V. corymbosum is considered autotetraploid, derived from a duplication of a diploid V. corymbosum (not a hybridization). A diploid V. corymbosum individual was used for genome sequencing; this is now indicated right after the informtion that VC is autotetraploid. (Line 158-162) V. arboreum is a different species in a different section of Vaccinium, now specified in the text. (Line 164-165). While some limited phylogenetics analysis of Vaccinium spp. has been completed, none include both the species we used for this study.

>Table S1. Add more detailed columns, so besides the column with the name, you have a description. So, VC_trimm_Uc can have a three extra columns explaining VC, trim and Uc. May seem redundant, but it will allow to interpret the table on its own.

As suggested, the extra columns are added.

>Line 157. How do you decide if it is significant?

A statistician was consulted during the interpretation of results, but because the statistical report did not contain all possible options, we decided not to include it. Significant is changed to low. (Line 182)

>Figure S1. You can coordinate the colours of the samples with the legend on Figure 1, to make everything consistent.

Colors have been made more consistent between the figures and to the rest of the figures in the paper.

>Line 196/Table 1. I would suggest to move this table to supplementals and show a boxplot with the size of the assemblies for each donation.

This table has been moved to the supplemental materials.

>Line 240. Detonate has not been described in the introduction, where other tools had been mentioned and how they work.

The tools mentioned in the introduction are all used in head-to-head comparisons. Tools used only to calculate metrics were not mentioned. However, it is a good idea to explain more about Detonate in the results. A sentence about it and the reference are now added to the Analysis section. (Line 217-222)

>Lile 272: Be consistent with the nomenclature. In the figure it is marked as "VC_4" and on text as "VC 4". You can rename the columns on your tables before plotting with something

like: gsub("_"," ", table$Assmbly_type) if you are using R.

The underscores on assembly type in figures are removed.

>Figure 3. The "transloc" and mult "bands" are hard to read, probably have this a supplemental table. I would also normalize the plot in percentages and have an extra panel with the number of transcripts that are used.

Mapping results are now provided as a table. Leaving total number of transcripts in the figure instead of using percents is intentional to visualize the global variations, and also, its not clear if it would be more informative to look at percents of total reads mapped or of total reads sequenced. However, to provide readers with either option, we have added the percentages in the supplemental file. This figure is now updated to improve compactness and visualization.

>Paragraph starting on Line 337: So from this paragraph, we may conclude that it is more important the number and volume of reads than the data processing? Maybe it would be worth to consider if the cost of sequencing more is cheaper than having more steps in the analysis? Or full transcript sequencing?

From these results, the suggestion is that if you have sequenced multiple samples, combining them may perform better than using them separately. Also, soft trimming has a positive effect. We find it to be impossible to estimate if the cost of analysis, which largely depends on the type of bioinformatics support available for each research group. Full transcript sequencing (IsoSeq) may help assembly, although this type of sequencing has higher error and requires error correction. Without testing we prefer not to make further suggestions about this method. Instead, we mention IsoSeq as an alternative method in the introduction (line 85-89).

>Paragraph starting on line 486: Did you evaluate how homoeologue genes affect the mapping? I'm wondering if during the clustering step you could be collapsing homoeologues in a single representation.

Current genomic resources in blueberry, like in most polyploids, do not include precise information on homoeolog sequences. As such, transcripts produced from homoeologues with less than 5% sequence variation, would be collapsed by CD-HIT, which affected 22% of sequences with very little effect on quality metrics. Considering the soft clustering method applied and high similarity of putative collapsed homoeologues, the global effect on read mapping is expected to be low. A sentence mentioning this is added at the beginning of the section (Lines 548-550). Specific to assembly clustering, possible collapse of homoeologs by clustering is mentioned as well (Line 517-520).

>Methods.

>Are the scripts/exact commands used for the analysis deposited somewhere? You could have a GitHub repository with your scripts or add them as supplemental (or both!)

Most of the work consisted of running external software on the command line. Basic instructions on how to run these are included in the manuscript. For some specific functions written by the authors, including the calculations of Jaccard scores and coverage, a package

of scripts was submitted to Gigascience and will be available as part of the publication through an ftp link. This should also be provided to the reviewers.

>List of abbreviations: Include all the abbreviations used, like "cor", "trim", etc.

Following the suggestion, the list has been updated.

>Reviewer #2: Major Concern:
>The authors benchmarked Control Reads against Treatment Reads, Single Sample against Multiple Samples as input, CD-HIT against RapClust for clustering, and five mappers including bowtie2, gsnap, stampy, star and hisat2 for mapping reads. But for assembly, the authors benchmarked only one transcriptome assembler, Trinity.

We now included three assemblers, see below.

>The authors claimed, "Trinity is commonly selected and has good performance" in line 94 and cited two papers. One paper titled "Optimizing de novo transcriptome assembly …" was published 2011, which is a bit outdated and doesn't include the benchmark of latest short-read transcriptome assemblers. The other paper "Comprehensive evaluation of de novo …" is new (2017) but doesn't support the authors claim and concluded in its abstract, quote: "SOAPdenovo-Trans performed best in base coverage, while Trans-ABySS performed best in gene coverage and number of recovered full-length transcripts. In terms of chimeric sequences, BinPacker and Oases-Velvet were the worst, while IDBA-tran, SOAPdenovo-Trans, Trans-ABySS and Trinity produced fewer chimeras across all single k-mer assemblies."

The claim of "good performance" is modified to "usually good quality", which is not contradicted with the references considering that in both of them, Trinity was best or second best at some quality metrics.

>As we know, transcriptome assemblers perform differently on genomes of different characteristics - Trinity usually performs better on mammals and vertebrates, SOAPdenovo-Trans on plants and Trans-ABySS on metagenomics. As the authors are targeting a "Comprehensive evaluation of RNA-Seq analysis pipelines", it is necessary to include another one or two leading transcriptome assemblers.

A comparison including assemblies from SOAPdenovo-Trans (due to the indicated usual better performance on plants, which we were not aware of), Trans-AbySS (which had also good performance in the references), and Trinity has been added.

>Minor Concerns:
>Cite Detonate score paper in line 240.

Citation was added.

Close
