# Supplementary material for: Comprehensive evaluation of RNA-seq analysis pipelines in diploid and polyploid species
Source: Gigascience. 2018 Nov 10;7(12):giy132. doi: 10.1093/gigascience/giy132 (PMC6275443; doi:10.1093/gigascience/giy132)
Supplement: Reviewer_1_Report_(Original_Submission) -- Ricardo Ramirez-Gonzalez — 5/22/2018 Reviewed [file giy132_reviewer_1_report_(original_submission).pdf]

Reviewer Report

Title: Comprehensive evaluation of RNA-Seq analysis pipelines in diploid and polyploid species

Version: Original Submission **Date:** 5/22/2018

Reviewer name: Ricardo Ramirez-Gonzalez

Reviewer Comments to Author:

General Comments.

The idea of comparing different assembly and mapping strategies is compelling. It is true, that there are few resources about the effects of polyplody on tools designed mostly for diploids. Since the mappings are already done, you could explore in more detail how multiple homoeologues may be mapping to the same "unigene", or you could try to figure out if the homoeologues are removed/merged into single unigenes. If that is the case, you may be mapping the tetraploid to a reference closer to a diploid. If the duplication event is recent, you can expect almost double of the genes in the tetraploid transcriptome, compared to the diploid.

The idea behind figure 1, that shows all the tools is nice. However, it can be improved to make the order of the pipeline more explicit. Also, the kind of algorithms, drawbacks, advantages, etc of each program used is scatter all over the place. It would be nice to have a table with all that information summarized, including one column with a short description of the final effect in each step of the analysisis analysis. A row could look like (with more rows, one for each step in the pipeline)

Tool: Trimomatic
De Novo Assembly: Improves in 5% on VC (or whatever you find)
Mapping to geneome: Limited effect.

The figures require a lot of work to make them look consistent (same colorus for same variables across the paper, for example).

Specific comments.

Figure 1.
General: It is confusing what are characteristics of the analysis (like individual/combined), programs (Is Rcorrector a program? A typo?). Some colour/font style change could help to distinguish them. The legend requires a lot of work, as it is not very descriptive of the elements represented. Also, the colours could be improved to reduce confusion. Yellow seems to represent "cor trim" and reference genome. Grey is for Cor skewer, but it is also used for Clustering.

Panel A: Cor skewer is not present in the diagram. Also, there is no explanation of what the crosses mean. Rcorrector is not defined in legend. The figure seems to suggest that Rcorrector and Trimmomatic/Skewer are two different pipelines, where in the text it is described as Rcorrector+Trimmmatic or Rcorrector+Skewer

Panel B: The boxes don't need to be colour coded, as the colours are not used elsewhere to link, and adds confusion as green are blue are used to represent transcripts and reads elsewhere in the figure.

Panel C: It is not clear that the top and the bottom diagrams are different things (De Novo vs reference guided).

Line 72. Illumina may still be cheaper, but it may be worth mentioning Iso-Seq, from PacBio that are already able to retrieve full transcripts. I understand it is beyond the scope of the paper, but it is worth mentioning.

Line 89. A supplementary figure showing how the different errors affect the assembly could help the unexperienced reader to understand why the errors happen.

Line 93. It is commonly selected, agreed. But how do you define good performance? Having used it before, the pipeline writes several temporary files, which computationally is not very performant. If it refers to the quality of the assembly, no other options are discussed in this paper, are there any other RNA-Seq assemblers?

Line 112. FM-Index is not defined. Hash tables are considered fast in computer science. You can argue that it depends on details of the implementations and how the different software compensate for the drawbacks (like doing a "proper " alignment once the region where the read maps is identified).

Line 136: Is Vaccinium corymbosum derived from a duplication of V. arborerum? if so, it may be worth to mention. It would also be nice to have a comparison of how distant they are.

Table S1. Add more detailed columns, so besides the column with the name, you have a description. So, VC_trimm_Uc can have a three extra columns explaining VC, trim and Uc. May seem redundant, but it will allow to interpret the table on its own.

Line 157. How do you decide if it is significant?

Figure S1. You can coordinate the colours of the samples with the legend on Figure 1, to make everything consistent.

Line 196/Table 1. I would suggest to move this table to supplementals and show a boxplot with the size of the assemblies for each donation.

Line 240. Detonate has not been described in the introduction, where other tools had been mentioned and how they work.

Lile 272: Be consistent with the nomenclature. In the figure it is marced as "VC_4" and on text as "VC 4". You can rename the columns on your tables before plotting with something like: gsub("_"," ", table$Assmbly_type) if you are using R.

Figure 3. The "transloc" and mult "bands" are hard to read, probably have this a supplemental table. I would also normalize the plot in percentages and have an extra panel with the number of transcripts that are used.

Paragraph starting on Line 337: So from this paragraph, we may conclude that it is more important the number and volume of reads than the data processing? Maybe it would be worth to consider if the cost of sequencing more is cheaper than having more steps in the analysis? Or full transcript sequencing?

Paragraph starting on line 486: Did you evaluate how homoeologue genes affect the mapping? I'm wondering if during the clustering step you could be collapsing homoeologues in a single representation.

Methods.

Are the scripts/excact commands used for the analysis deposited somewhere? You could have a github repository with your scripts or add them as supplemental (or both!)

List of abbreviations: Include all the abbreviations used, like "cor", "trim", etc.

Methods

Are the methods appropriate to the aims of the study, are they well described, and are necessary controls included? Choose an item.

Conclusions

Are the conclusions adequately supported by the data shown? Choose an item.

Reporting Standards

Does the manuscript adhere to the journal's guidelines on [minimum standards of reporting?] Choose an item.

Choose an item.

Statistics

Are you able to assess all statistics in the manuscript, including the appropriateness of statistical tests used? Choose an item.

Quality of Written English

Please indicate the quality of language in the manuscript: Choose an item.

Declaration of Competing Interests

Please complete a declaration of competing interests, considering the following questions:

- Have you in the past five years received reimbursements, fees, funding, or salary from an organisation that may in any way gain or lose financially from the publication of this manuscript, either now or in the future?
- Do you hold any stocks or shares in an organisation that may in any way gain or lose financially from the publication of this manuscript, either now or in the future?
- Do you hold or are you currently applying for any patents relating to the content of the manuscript?
- Have you received reimbursements, fees, funding, or salary from an organization that holds or has applied for patents relating to the content of the manuscript?

- Do you have any other financial competing interests?
- Do you have any non-financial competing interests in relation to this paper?

If you can answer no to all of the above, write 'I declare that I have no competing interests' below. If your reply is yes to any, please give details below.

I declare that I have no competing interests

I agree to the open peer review policy of the journal. I understand that my name will be included on my report to the authors and, if the manuscript is accepted for publication, my named report including any attachments I upload will be posted on the website along with the authors' responses. I agree for my report to be made available under an Open Access Creative Commons CC-BY license (http://creativecommons.org/licenses/by/4.0/). I understand that any comments which I do not wish to be included in my named report can be included as confidential comments to the editors, which will not be published.

I agree to the open peer review policy of the journal

To further support our reviewers, we have joined with Publons, where you can gain additional credit to further highlight your hard work (see: https://publons.com/journal/530/gigascience). On publication of this paper, your review will be automatically added to Publons, you can then choose whether or not to claim your Publons credit. I understand this statement.

Yes Choose an item.
