# Supplementary material for: Comprehensive evaluation of RNA-seq analysis pipelines in diploid and polyploid species
Source: Gigascience. 2018 Nov 10;7(12):giy132. doi: 10.1093/gigascience/giy132 (PMC6275443; doi:10.1093/gigascience/giy132)
Supplement: Reviewer_2_Report_(Original_Submission) -- Ruibang Luo — 2/6/2018 Reviewed [file giy132_reviewer_2_report_(original_submission).pdf]

Reviewer Report

Title: Comprehensive evaluation of RNA-Seq analysis pipelines in diploid and polyploid species

Version: Original Submission Date: 6/2/2018

Reviewer name: Ruibang Luo

Reviewer Comments to Author:

Major Concern:
The authors benchmarked Control Reads against Treatment Reads, Single Sample against Multiple Samples as input, CD-HIT against RapClust for clustering, and five mappers including bowtie2, gsnap, stampy, star and hisat2 for mapping reads. But for assembly, the authors benchmarked only one transcriptome assembler, Trinity. The authors claimed, "Trinity is commonly selected and has good performance" in line 94 and cited two papers. One paper titled "Optimizing de novo transcriptome assembly …" was published 2011, which is a bit outdated and doesn't include the benchmark of latest short-read transcriptome assemblers. The other paper "Comprehensive evaluation of de novo …" is new (2017) but doesn't support the authors claim and concluded in its abstract, quote: "SOAPdenovo-Trans performed best in base coverage, while Trans-ABySS performed best in gene coverage and number of recovered full-length transcripts. In terms of chimeric sequences, BinPacker and Oases-Velvet were the worst, while IDBA-tran, SOAPdenovo-Trans, Trans-ABySS and Trinity produced fewer chimeras across all single k-mer assemblies." As we know, transcriptome assemblers perform differently on genomes of different characteristics - Trinity usually performs better on mammals and vertebrates, SOAPdenovo-Trans on plants and Trans-ABySS on metagenomics. As the authors are targeting a "Comprehensive evaluation of RNA-Seq analysis pipelines", it is necessary to include another one or two leading transcriptome assemblers.

Minor Concerns:
Cite Detonate score paper in line 240.

Methods

Are the methods appropriate to the aims of the study, are they well described, and are necessary controls included? Choose an item.

Conclusions

Are the conclusions adequately supported by the data shown? Choose an item.

Reporting Standards

Does the manuscript adhere to the journal's guidelines on [minimum standards of reporting?]() Choose an item.

Choose an item.

Statistics

Are you able to assess all statistics in the manuscript, including the appropriateness of statistical tests used? Choose an item.

Quality of Written English

Please indicate the quality of language in the manuscript: Choose an item.

Declaration of Competing Interests

Please complete a declaration of competing interests, considering the following questions:

- Have you in the past five years received reimbursements, fees, funding, or salary from an organisation that may in any way gain or lose financially from the publication of this manuscript, either now or in the future?
- Do you hold any stocks or shares in an organisation that may in any way gain or lose financially from the publication of this manuscript, either now or in the future?
- Do you hold or are you currently applying for any patents relating to the content of the manuscript?
- Have you received reimbursements, fees, funding, or salary from an organization that holds or has applied for patents relating to the content of the manuscript?
- Do you have any other financial competing interests?
- Do you have any non-financial competing interests in relation to this paper?

If you can answer no to all of the above, write 'I declare that I have no competing interests' below. If your reply is yes to any, please give details below.

I declare that I have no competing interests

I agree to the open peer review policy of the journal. I understand that my name will be included on my report to the authors and, if the manuscript is accepted for publication, my named report including any attachments I upload will be posted on the website along with the authors' responses. I agree for my report to be made available under an Open Access Creative Commons CC-BY license (http://creativecommons.org/licenses/by/4.0/). I understand that any comments which I do not wish to be included in my named report can be included as confidential comments to the editors, which will not be published.

I agree to the open peer review policy of the journal

To further support our reviewers, we have joined with Publons, where you can gain additional credit to further highlight your hard work (see: https://publons.com/journal/530/gigascience). On publication of this paper, your review will be automatically added to Publons, you can then choose whether or not to claim your Publons credit. I understand this statement.

Yes Choose an item.
